# GRAF2, WDR44, and MICAL1 mediate Rab8/10/11–dependent export of E-cadherin, MMP14, and CFTR ΔF508

**DOI:** 10.1083/jcb.201811014

**Published:** 2020-04-28

**Authors:** Safa Lucken-Ardjomande Häsler, Yvonne Vallis, Mathias Pasche, Harvey T. McMahon

**Affiliations:** Medical Research Council Laboratory of Molecular Biology, Cambridge, UK

## Abstract

In addition to the classical pathway of secretion, some transmembrane proteins reach the plasma membrane through alternative routes. Several proteins transit through endosomes and are exported in a Rab8-, Rab10-, and/or Rab11-dependent manner. GRAFs are membrane-binding proteins associated with tubules and vesicles. We found extensive colocalization of GRAF1b/2 with Rab8a/b and partial with Rab10. We identified MICAL1 and WDR44 as direct GRAF-binding partners. MICAL1 links GRAF1b/2 to Rab8a/b and Rab10, and WDR44 binds Rab11. Endogenous WDR44 labels a subset of tubular endosomes, which are closely aligned with the ER via binding to VAPA/B. With its BAR domain, GRAF2 can tubulate membranes, and in its absence WDR44 tubules are not observed. We show that GRAF2 and WDR44 are essential for the export of neosynthesized E-cadherin, MMP14, and CFTR ΔF508, three proteins whose exocytosis is sensitive to ER stress. Overexpression of dominant negative mutants of GRAF1/2, WDR44, and MICAL1 also interferes with it, facilitating future studies of Rab8/10/11–dependent exocytic pathways of central importance in biology.

## Teaser

Integral proteins can reach the plasma membrane via different routes. Here, Lucken-Ardjomande Häsler et al. identify three proteins that are associated with dynamic intracellular tubules, closely aligned with the ER, and involved in the transport of specific cargos whose export is particularly sensitive to ER stress.

## Introduction

In eukaryotic cells, the ER is the birthplace of the majority of membrane proteins, secreted proteins, and lipids. Despite the canonical belief that they follow the same route from the ER through the ER-Golgi intermediate compartment (ERGIC), Golgi, and TGN to reach the plasma membrane, differences between individual cargos exist. Lipids can be transferred between membranes at contact sites (Nishimura and Stefan, 2020). Some proteins transit via tubular endosomes (Desclozeaux et al., 2008; Henry and Sheff, 2008; Ang et al., 2004; Monis et al., 2017), others such as MMP14 (also called MT1-MMP) and GLUT4 are stored in vesicles for timed or targeted release (Bravo-Cordero et al., 2007; Watson et al., 2004), and a few, such as CFTR and interleukin-1β, can enter a route opened by ER stress (Dupont et al., 2011; Gee et al., 2011). These differences might arise from binding to different adapters or partitioning in membrane domains, which could lead to proteins exiting the classical pathway of secretion at any step (Marie et al., 2009; Chen et al., 2017; Hoffmeister et al., 2011; Stephens and Pepperkok, 2004). Under certain conditions, some integral proteins and lipids are still exported when cells are incubated with Brefeldin A (BFA), which among other things leads to dissolution of the Golgi into the ER (Fujiwara et al., 1988). These cargos have been proposed to bypass the Golgi and are said to follow an unconventional pathway of secretion. Among the cargos that have been reported to reach the plasma membrane in the presence of BFA are E-cadherin (Low et al., 1992), MMP14 (Deryugina et al., 2004), CFTR (Rennolds et al., 2008; Gee et al., 2011), and the ciliary protein Polycystin-1 (Gilder et al., 2018). Whether these cargos actually bypass the Golgi and follow the same route out of the ER is unclear, but what they also have in common is that their export depends on a small group of Rabs.

Rabs are regulators of intracellular transport whose GTP-GDP cycle drives membrane trafficking processes forward. Rab8 controls the export of MMP14 (Bravo-Cordero et al., 2007; Wiesner et al., 2013), Rab11 mediates the export of E-cadherin (Lock and Stow, 2005; Desclozeaux et al., 2008), a Rab11-Rab8 cascade regulates the apical transport of CFTR (Vogel et al., 2015), and Rab8, Rab10, and Rab11 cooperate in the export of neosynthesized proteins to the primary cilium (Knödler et al., 2010; Sato et al., 2014). In the case of E-cadherin, CFTR, and primary cilia proteins, subsets of recycling endosomes are traversed en route to the cell surface (Monis et al., 2017; Desclozeaux et al., 2008; Vogel et al., 2017). Indeed, Rab11 and to a lesser extent Rab8 and Rab10 are also involved in the recycling of several endocytosed plasma membrane proteins, such as Integrin-β1 (Powelka et al., 2004; Sharma et al., 2009; Hülsbusch et al., 2015) or the Transferrin receptor (Ullrich et al., 1996; Roland et al., 2011; Babbey et al., 2006). While Rab8-, Rab10-, and Rab11-binding partners have been identified (Peränen, 2011; Welz et al., 2014; Chua and Tang, 2018), we still do not understand how Rab-dependent protein export happens at a molecular level.

Of particular interest for trafficking pathways connected to recycling endosomes, recent data indicate that membrane tubulating proteins of the GRAF family (GRAF1, GRAF2, GRAF3, and Oligophrenin 1 [OPHN1]) can participate both in endocytic and exocytic routes. On the endocytic side, OPHN1 regulates clathrin- and Endophilin-dependent endocytosis in neuronal cells (Khelfaoui et al., 2009; Nakano-Kobayashi et al., 2009), while GRAF1 was proposed to mediate clathrin-independent endocytosis of soluble dextran and of the cholera toxin CTxB in HeLa cells (Lundmark et al., 2008), of CD44 in MDA-MB-231 cells (Bendris et al., 2016), and of the EGF receptor (EGFR) in *Drosophila* plasmatocytes (Kim et al., 2017). Conversely, OPHN1 controls exocytosis at pre- and postsynaptic sites (Powell et al., 2012; Nadif Kasri et al., 2009) and in chromaffin cells (Houy et al., 2015); GRAF1c was proposed to participate in Integrin-β1 recycling (Cai et al., 2014); GRAF1 and GRAF2 mediate recycling of fusogenic Ferlins in differentiated C2C12 myoblasts (Doherty et al., 2011b; Lenhart et al., 2014); and GRAF1 was reported to regulate autophagy-dependent secretion of TGFB1 (Nüchel et al., 2018).

Little is known about the molecular mechanisms governing GRAF1/2–mediated pathways as among the direct binding partners identified (FAK, PYK2, PKNβ, Dynamin, GIT1, Cdc42, ILK, FGD6, and EGFR; Hildebrand et al., 1996; Ren et al., 2001; Shibata et al., 2001; Francis et al., 2015; Doherty et al., 2011a; Lundmark et al., 2008; Nüchel et al., 2018; Steenblock et al., 2014; Kim et al., 2017); only Cdc42 may directly regulate GRAF-dependent trafficking, and apart from Dynamin, none is a bona fide membrane trafficking protein. After observing colocalization between GRAF1b/2 and Rab8 and Rab10, we identified MICAL1 and WDR44 (also called Rabphilin-11) as direct GRAF1b/2–binding partners. MICAL1 and WDR44 are also associated with dynamic tubules and bind to Rab8/10 and Rab11, respectively. Endogenous WDR44 tubules can be described as a subset of tubular endosomes that are closely aligned with the ER via binding to VAPA and VAPB. Although GRAF2 and WDR44 are not involved in Transferrin or Integrin-β1 recycling, they participate in the export of neosynthesized E-cadherin, MMP14, and CFTR ΔF508.

## Results

### GRAF1b and GRAF2 colocalize with Rab8a/b and Rab10

**Figure 1.**
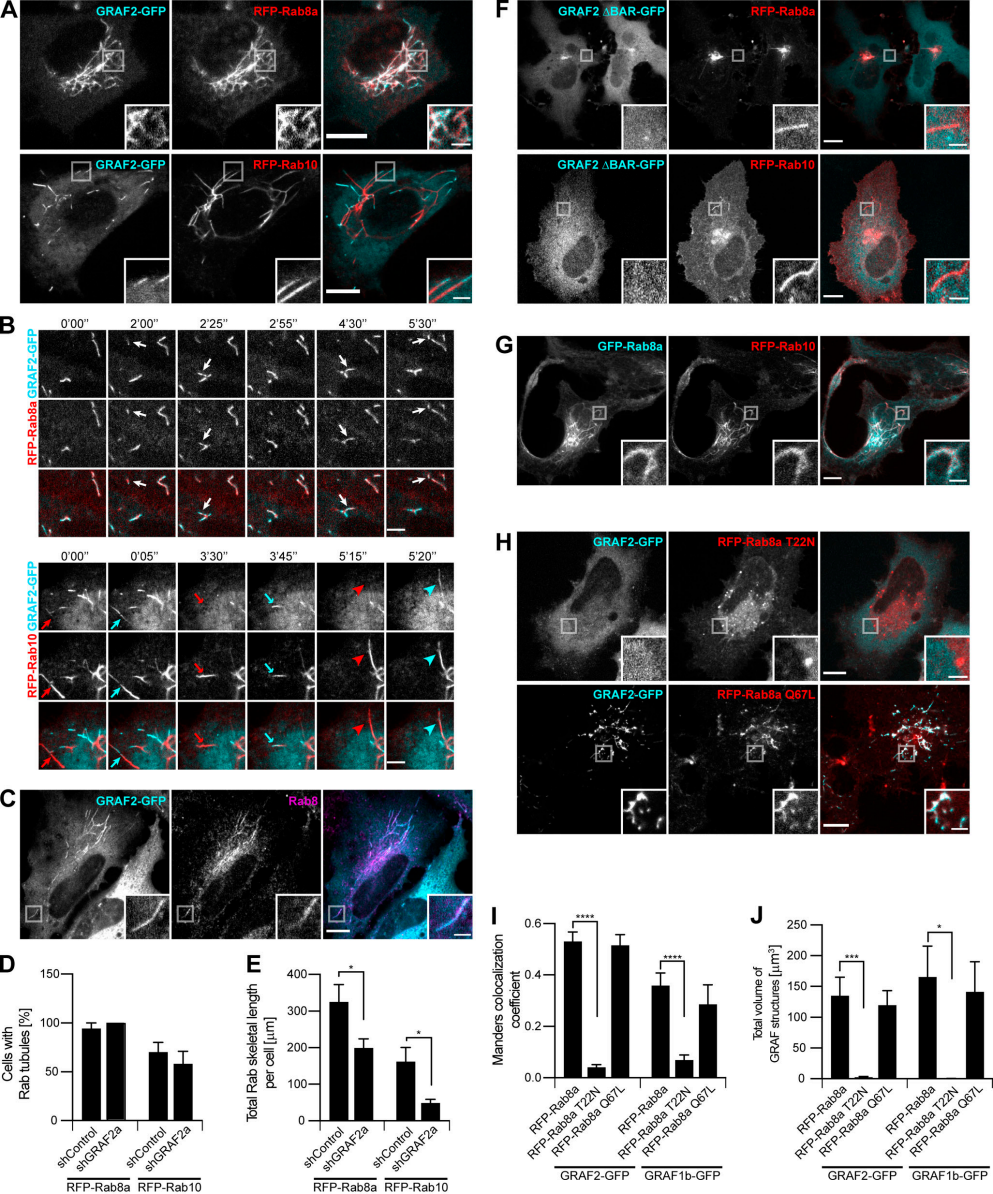
**GRAF1b/2 colocalize with Rab8a/b and Rab10 in HeLa cells. (A and B)** Confocal images of transfected live cells showing overlapping (RFP-Rab8a) or contiguous (RFP-Rab10) colocalization with GRAF2-GFP on intracellular tubules. **(B)** Snapshots were taken every 5 s. Regions with dynamic tubules are shown at relevant time points. Rab8a and GRAF2 appeared simultaneously (white arrows), but Rab10 (red arrows/arrowheads) preceded GRAF2 (cyan arrows/arrowheads). Scale bars: 5 µm. **(C)** Confocal images of transfected cells stained with α-Rab8 showing Rab8 on GRAF2-GFP tubules. **(D and E)** shControl or shGRAF2a-expressing cells were transfected with RFP-Rab8a or Rab10. **(D)** Proportion of cells with Rab8a/10–positive tubules. *n* = 3. **(E)** Total skeletal length of RFP-Rab8a/10 structures per cell. *n* = 24–40 cells. **(F–H)** Confocal images of transfected cells. **(F)** GRAF2 ΔBAR-GFP was not found on RFP-Rab8a/10 tubules. **(G)** GFP-Rab8a and RFP-Rab10 colocalized. **(H)** GRAF2-GFP was diffuse when expressed with RFP–Rab8a T22N but colocalized with RFP–Rab8a Q67L. **(I and J)** Cells were cotransfected with GRAF1b/2–GFP and RFP-Rab8a, Rab8a T22N, or Rab8a Q67L. **(I)** Manders colocalization coefficients for GRAF1b/2 on Rab8a-positive structures. **(J)** Total volume of GRAF1b/2-GFP–positive structures per cell. **(I and J)** *n* = 15–45 cells. **(D, E, I, and J) **Data are means ± SEM; *, P < 0.05; ***, P < 0.001; and ****, P < 0.0001. **(A, C, and F–H)** Insets show magnifications of the boxed areas. Scale bars: 10 µm; scale bars of insets: 2 µm.

**Figure S1.**
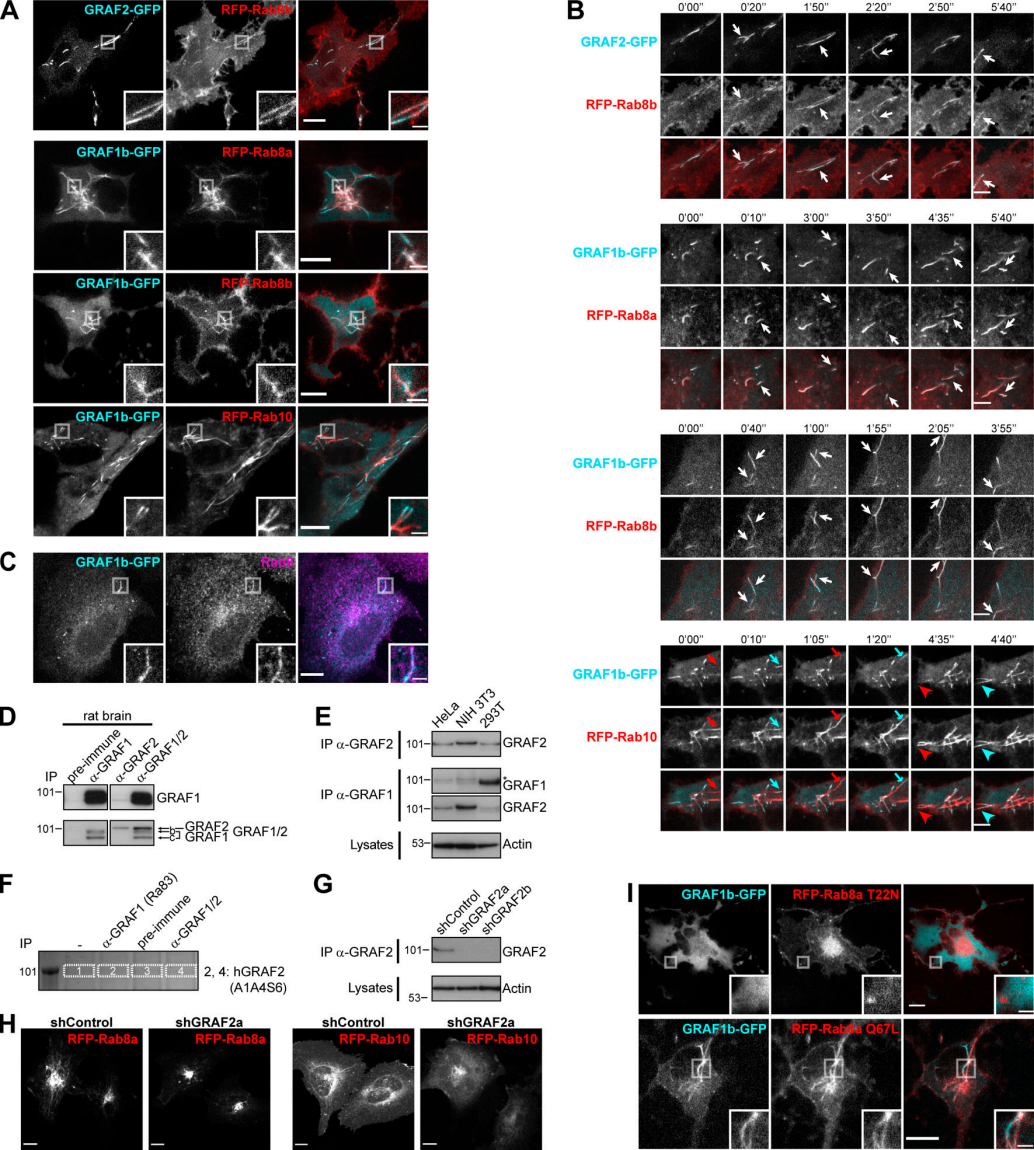
**GRAF1b/2 colocalize with Rab8a/b and Rab10 in HeLa cells. (A and B)** Confocal images of transfected live cells showing overlapping (RFP–Rab8a/b) or contiguous (RFP-Rab10) colocalization with GRAF1b/2–GFP on tubules. **(B)** Snapshots were taken every 5 s. Regions with dynamic tubules are shown at relevant time points. Rab8a/b and GRAF1b/2 appeared simultaneously (white arrows), but Rab10 (red arrows/arrowheads) preceded GRAF1b (cyan arrows/arrowheads). Scale bars: 5 µm. **(C)** Confocal images of cells transfected with GRAF1b-GFP and stained with α-Rab8 showing colocalization. **(D)** Immunoprecipitation (IP) of GRAF proteins from an equal amount of rat brain lysate using a purified antibody against GRAF1 (α-GRAF1), a purified antibody against GRAF2 (α-GRAF2), or unpurified serum detecting both GRAF1 and GRAF2 (α-GRAF1/2). Pre-immune serum was used as negative control. Top and bottom membranes are replicates. GRAF2 migrates at a higher molecular weight than all GRAF1 isoforms. **(E)** GRAF proteins were immunoprecipitated from an equal protein amount of HeLa, NIH 3T3, and 293T cell lysates using α-GRAF2 (top) or α-GRAF1 (middle). Actin was used as loading control on the corresponding lysates (bottom). IP with α-GRAF2 showed GRAF2 expression in the three cell lines. IP with α-GRAF1 showed GRAF1 expression in 293T cells. On this membrane, the faint band seen in HeLa and NIH 3T3 samples (*) corresponded to traces of GRAF2 protein, as it was at a higher molecular weight than the GRAF1 band and its intensity was increased after stripping and reprobing of the membrane with α-GRAF2. This suggests that in lysates containing low amounts of GRAF1, α-GRAF1 can cross-react with GRAF2. **(F)** Equal amounts of a HeLa cell lysate were immunoprecipitated with an α-GRAF1 antibody described in earlier studies (Ra83), pre-immune serum, or α-GRAF1/2 serum. Proteins bound to beads were separated by electrophoresis and stained with Coomassie. The region around the mol wt for GRAFs was cut and analyzed by LC-MS/MS. Lanes 2 and 4 contained GRAF2 (Uniprot accession no. A1A4S6), but no GRAF1 peptides were identified. **(G)** IP****of endogenous GRAF2 from an equal amount of shRNA-transfected HeLa cell lysates. Beads and lysates were analyzed in parallel, showing knockdown of GRAF2 in shGRAF2a- and shGRAF2b-transfected cells. Actin was used as loading control. **(H)** Confocal stacks of HeLa cells expressing shControl or shGRAF2a and transfected with RFP-Rab8a or RFP-Rab10. **(I)** Confocal images of transfected live cells. GRAF1b-GFP was diffuse when expressed with RFP-Rab8a T22N but colocalized with RFP–Rab8a Q67L. **(A, C, H, and I)** Insets show magnifications of the boxed areas. Scale bars: 10 µm; scale bars of insets: 2 µm.

In HeLa cells, a prototypical epithelial cell line with well-characterized membrane trafficking pathways, GRAF1b and GRAF2 are associated with dynamic tubules and vesicles (Lucken-Ardjomande Häsler et al., 2014). To identify the Rabs associated with GRAF-mediated trafficking, we screened live cells for colocalization with GRAF1b and GRAF2. GRAF1b/2 colocalized with Rab8a/b and displayed a striking contiguous distribution with Rab10 on the same tubules (Fig. 1 A and Fig. S1 A). GRAF1b/2 colocalized with Rab8a/b throughout the lifetime of the tubules, but GRAF1b/2 were recruited to preexisting Rab10 tubules (Fig. 1 B and Fig. S1 B). In fixed cells, GRAF1b/2 also colocalized with endogenous Rab8 (Fig. 1 C and Fig. S1 C). Down-regulation of GRAF2 expression (the main GRAF of HeLa cells; Fig. S1, D–F) with transfection of an shRNA-encoding plasmid specific for GRAF2 (shGRAF2a; Fig. S1 G) did not change the percentage of cells with RFP-Rab8 or Rab10 tubules (Fig. 1 D). It led however to a decrease in the total skeletal length of the tubules (Fig. 1 E and Fig. S1 H). The BAR domain of GRAF2 was essential, as GRAF2 ΔBAR was cytosolic and failed to colocalize with Rab8a/10 tubules (Fig. 1 F).

Rab8 and Rab10 regulate the same intracellular trafficking pathways (Sato et al., 2014; Sano et al., 2008; Sun et al., 2010; Homma and Fukuda, 2016). In agreement, Rab8a and Rab10 labeled the same network of intracellular membranes (Fig. 1 G). Our data suggest that Rab8 may promote the recruitment of GRAF1b/2 to Rab10 tubules. Indeed, upon coexpression of the dominant negative mutant Rab8a T22N (Peränen et al., 1996), GRAF1b/2 were cytosolic (Fig. 1, H–J; and Fig. S1 I). Reciprocally, the constitutively active mutant Rab8a Q67L colocalized with GRAF1b/2 (Fig. 1, H–J; and Fig. S1 I). These observations show that GRAF1b/2 are associated with the same intracellular transport intermediates as Rab8 and Rab10 and demonstrate a requirement of Rab8 activation for GRAF-positive compartments to form.

### GRAF1b and GRAF2 bind to MICAL1 and WDR44

**Figure S2.**
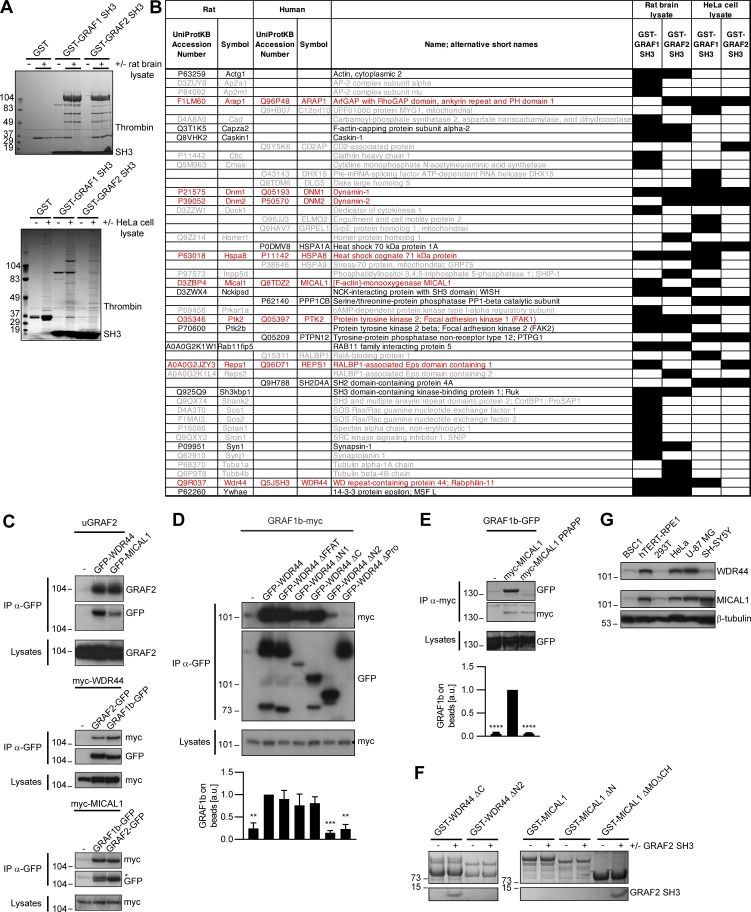
**GRAF1b/2 bind to MICAL1 and WDR44. (A)** Purified GST, GST–GRAF1 SH3, or GST–GRAF2 SH3 was incubated with or without rat brain or HeLa cell lysates and pulled down on glutathione sepharose beads. Bound proteins were eluted by Thrombin digestion and visualized by electrophoresis and Coomassie staining. **(B)** Table summarizing proteins identified in (A) by LC-MS/MS. Cells filled in black indicate samples where the proteins were found. Proteins only identified in one pull-down are in gray (26/47 hits); those found both in GST–GRAF1 SH3 and in GST–GRAF2 SH3 pull-downs but only in one lysate are in black (13/47 hits); and those found both in rat brain and in HeLa cell lysates are in red (8/47 hits). Since GRAF1 and GRAF2 have a 67.9% identity and a 91.1% similarity in their SH3 domains, we focused our attention on these eight robust interacting partners. **(C–E)** Immunoprecipitation (IP) of transfected 293T cells with α-GFP (C and D) or α-myc–coated beads (E). **(C)** uGRAF2 was coimmunoprecipitated by GFP-tagged WDR44 and MICAL1. myc-tagged WDR44 and MICAL1 were coimmunoprecipitated by GFP-tagged GRAF1b and GRAF2. In the beads, GRAF2-GFP and GRAF1b-GFP overlapped with the remaining myc-MICAL1 band (*). **(D and E)** The efficiency of GRAF1b binding was quantified using coimmunoprecipitation with GFP-WDR44 (D) or myc-MICAL1 (E) as reference. *n* = 3–5. Data are means ± SEM; **, P < 0.01; ***, P < 0.001; and ****, P < 0.0001. Binding was abolished by removal (D) or mutation (E) of a proline-rich region. **(D)** Overexpressed WDR44 underwent proteolysis. **(F)** Pull-down test of GRAF2 SH3 by GST–WDR44 ΔC, GST–WDR44 ΔN2, GST-MICAL1, GST–MICAL1 ΔN, and GST–MICAL1 ΔMOΔCH. Beads were analyzed by electrophoresis and Coomassie staining showing direct binding of GRAF2 SH3 to WDR44 ΔC and to MICAL1 ΔMOΔCH. GRAF2 SH3 did not bind MICAL1 or MICAL1 ΔN. This suggests that the PPKPP motif of MICAL1 is not accessible in the isolated full-length protein and that in order to be exposed, MICAL1 has to undergo a conformational change, such as the one induced by Rab-binding (Schmidt et al., 2008). **(G)** Western blot analysis of an equal amount of BSC1, hTERT-RPE1, 293T, HeLa, U-87 MG, and SH-SY5Y cell lysates loaded on two replicate gels. One membrane was probed with α-WDR44, the other with α-MICAL1. β-tubulin was used as loading control.

**Figure 2.**
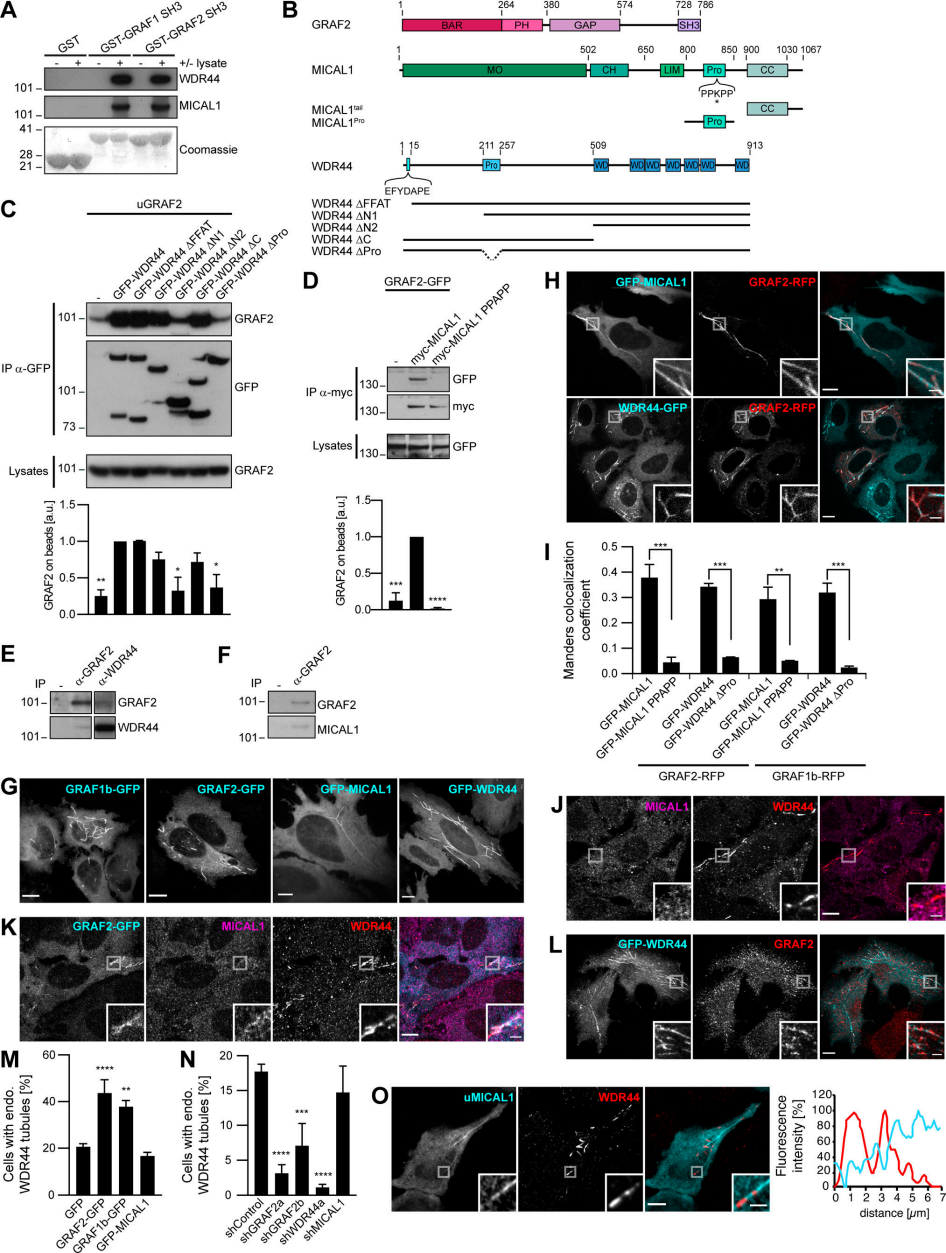
**GRAF1b and GRAF2 bind to MICAL1 and WDR44. (A)** Pull-down of HeLa cell proteins by GST, GST–GRAF1 SH3, or GST–GRAF2 SH3, analyzed on two replicate membranes. One was probed with α-WDR44, the other with α-MICAL1. They were then stained with Coomassie, revealing GST-tagged proteins. **(B)** Schematic representation of human GRAF2, MICAL1, and WDR44 and of mutants used. GRAF2 has two membrane-binding domains, BAR and PH, a GTPase Activating Protein (GAP) domain, and an SH3 domain. MICAL1 has an NADPH-binding MO domain, CH and LIM domains, a proline-rich region with a PPKPP motif, and a C-terminal coiled coil (CC). * indicates K832 mutated in MICAL1 PPAPP. WDR44 has an N-terminal FFAT motif, a proline-rich region, and seven WD repeats. **(C and D)** Immunoprecipitation (IP) of transfected 293T cells with α-GFP (C) or using α-myc–coated beads (D). The efficiency of GRAF2 binding (untagged GRAF2 [uGRAF2]; C) or GRAF2-GFP (D) was quantified, using coimmunoprecipitation with GFP-WDR44 (C) or myc-MICAL1 (D) as reference. *n* = 3 or 4. Binding was decreased by removal (C) or mutation (D) of proline-rich regions. **(C)** Note that GFP-WDR44 underwent proteolysis. **(E)** Endogenous WDR44 and GRAF2 were coimmunoprecipitated from a HeLa cell lysate using α-GRAF2 and α-WDR44, but not without antibody (−). The membrane was probed with α-GRAF2, followed by α-WDR44. The left and right parts of the membrane were treated identically. **(F)** Endogenous MICAL1 was coimmunoprecipitated from an SH-SY5Y cell lysate using α-GRAF2, but not without antibody (−). The membrane was probed with α-GRAF2, followed by α-MICAL1. **(G)** Confocal stacks of transfected HeLa cells. GFP-tagged GRAF1b, GRAF2, MICAL1, and WDR44 were cytosolic and associated with intracellular tubules. **(H)** Confocal images of transfected HeLa cells. GRAF2-RFP colocalized with GFP-tagged MICAL1 and WDR44. **(I)** Manders colocalization coefficients for GFP-tagged MICAL1 and WDR44 proteins on GRAF1b/2-RFP–associated structures. *n* = 15–30 cells. **(J–L)** Confocal images of HeLa cells stained with α-MICAL1 and α-WDR44 (J and K) or α-GRAF2 (L). **(J)** Untransfected cells had distinct WDR44 tubules but showed a diffuse distribution of MICAL1. **(K)** Endogenous WDR44 and to a lesser extent endogenous MICAL1 colocalized with GRAF2-GFP. **(L)** Endogenous GRAF2 was found on GFP-WDR44 tubules. **(M and N)** Percentage of transfected HeLa cells with endogenous (endo.) WDR44 tubules. **(M)** *n* = 4–8. **(N)** *n* = 3–8. **(O)** Confocal images of transfected HeLa cells stained with α-WDR44 and fluorescence intensity profiles along the tubule enlarged in the boxed area showing complementary distribution of untagged MICAL1 (uMICAL1, cyan line) and endogenous WDR44 (red line) on the same tubule. **(C, D, I, M, and N)** Data are means ± SEM; *, P < 0.05; **, P < 0.01; ***, P < 0.001; and ****, P < 0.0001. **(G, H, J–L, and O)** Insets show magnifications of the boxed areas. Scale bars: 10 µm; scale bars of insets: 2 µm.

We sought to determine how GRAF1b/2 might be connected to Rab8 and Rab10, since there was no direct interaction. We performed pull-downs from rat brain and HeLa cell lysates using GRAF SH3 domains as baits, as they are their only protein–protein interaction domain and SH3 domains are enriched in proteins associated with small GTPase signaling (Xin et al., 2013; Fig. S2 A). 47 proteins were identified, of which 8 were pulled down from both lysates (Fig. S2 B). Among these eight, two—MICAL1 and WDR44—are Rab-binding proteins. We confirmed pull-down of endogenous MICAL1 and WDR44 by GRAF1/2 SH3 (Fig. 2 A) and coimmunoprecipitation of the overexpressed proteins in 293T cells (Fig. S2 C). 293T cells were chosen for biochemical assays because of their high efficiency of transfection and protein expression. In both cases, GRAF1b/2 bound a proline-rich motif, as binding was abolished by deletion of the proline-rich region of WDR44 (Fig. 2, B and C; and Fig. S2 D) and by substitution of a single aa (K832A) within the PPKPP motif of MICAL1 (Fig. 2, B and D; and Fig. S2 E). Binding was direct, since GRAF2 SH3 bound to WDR44 ΔC and MICAL1 ΔMOΔCH in vitro (Fig. S2 F). Western blot analysis showed that WDR44 and MICAL1 were expressed in a variety of cell lines (Fig. S2 G). Endogenous GRAF2 coimmunoprecipitated WDR44 from lysates of HeLa cells (Fig. 2 E) and MICAL1 from lysates of SH-SY5Y cells (Fig. 2 F), which have a higher MICAL1/WDR44 expression ratio (Fig. S2 G). WDR44 and MICAL1 are thus two novel direct GRAF1b/2–binding proteins.

**Figure S3.**
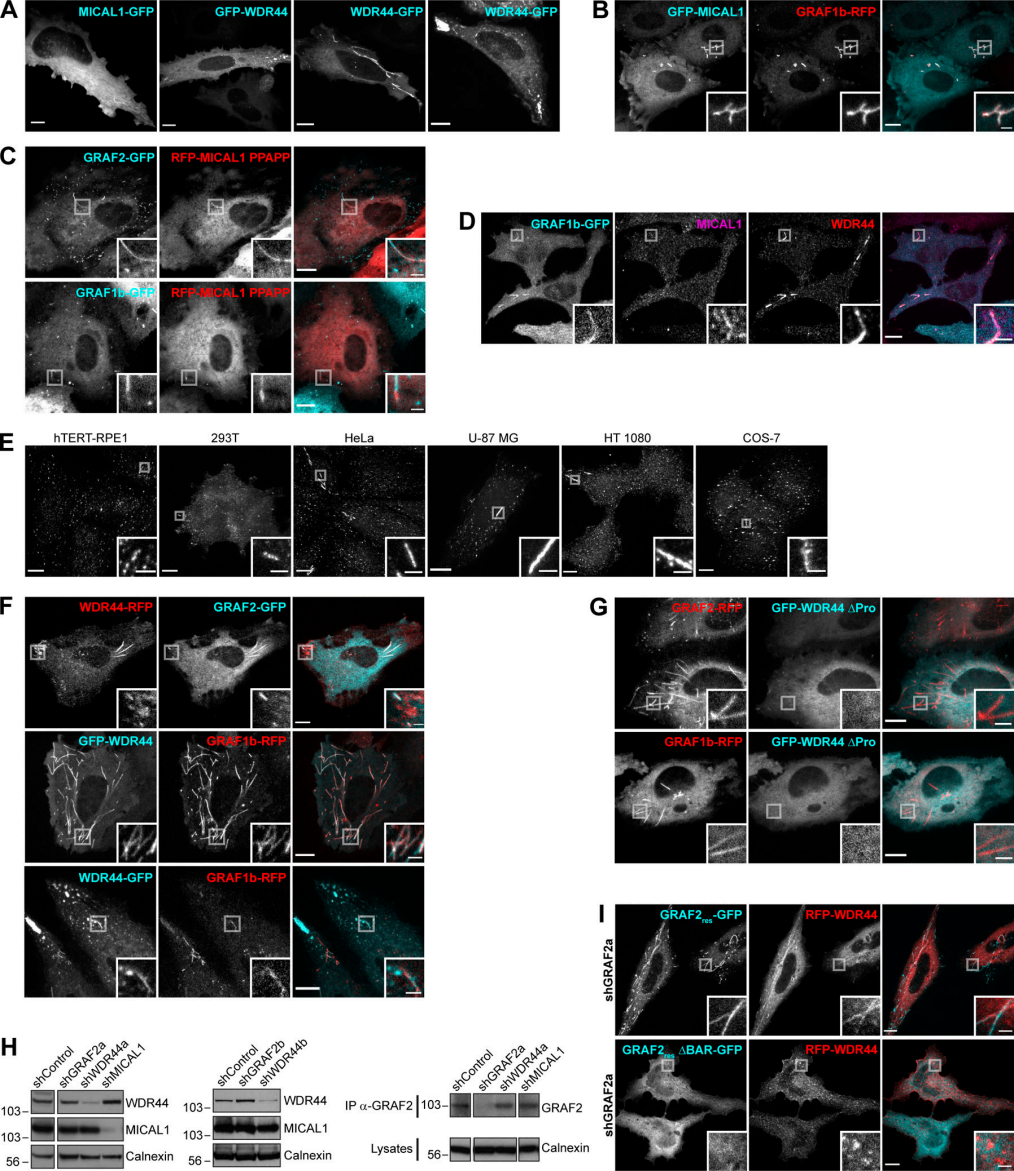
**GRAF1b/2 colocalize with MICAL1 and WDR44. (A)** Confocal stacks of transfected HeLa cells showing a similar distribution of N- and C-terminal–tagged MICAL1 and WDR44 (compare with Fig. 2 G). In some cells, WDR44 was found on irregular peripheral patches. **(B)** Confocal images of transfected HeLa cells showing GRAF1b-RFP on the same intracellular tubules as GFP-MICAL1. **(C)** Confocal images of transfected HeLa cells showing that although MICAL1 PPAPP did not efficiently colocalize with GRAF1b/2 (Fig. 2 I), it was sometimes found on GRAF-positive tubules (boxed areas). **(D)** Confocal images of transfected HeLa cells stained with α-MICAL1 and α-WDR44. Endogenous WDR44 and to a lesser extent endogenous MICAL1 colocalized with GRAF1b-GFP. **(E)** Confocal stacks of hTERT-RPE1, 293T, HeLa, U-87 MG, HT 1080, and COS-7 cells stained with α-WDR44. Endogenous WDR44 tubules of various lengths were found in all but hTERT-RPE1 cells. Tubules were more abundant and longer in HeLa, HT 1080, and U-87 MG cells. **(F)** Confocal images of transfected HeLa cells showing colocalization of GRAF1b/2 with WDR44 tubules but not with its peripheral patches. **(G)** Confocal images of transfected HeLa cells showing that WDR44 ΔPro was not recruited to GRAF1b/2 tubules. **(H)** Western blot analysis of shRNA-transfected HeLa cell lysates. Calnexin was used as loading control. Left and middle: Specific knockdown of WDR44 in cells expressing the shRNA-enconding plasmids shWDR44a and shWDR44b and of MICAL1 in cells expressing shMICAL1. The two parts of the left blots are from the same membrane and correspond to identical exposure times. Right: Immunoprecipitation (IP) of endogenous GRAF2 from equal amounts of cell lysates and Western blot analysis, showing specific knockdown of GRAF2 in shGRAF2a-transfected cells. The three parts of the blots are from the same membranes and correspond to identical exposure times. **(I)** Confocal images of shGRAF2a-expressing HeLa cells. The shGRAF2a-resistant protein GRAF2_res_-GFP and RFP-WDR44 colocalized on intracellular tubules; GRAF2_res_ ΔBAR–GFP was diffuse and led to RFP-WDR44 being only found on puncta. **(A–G and I)** Insets show magnifications of the boxed areas. Scale bars: 10 µm; scale bars of insets: 2 µm.

In live HeLa cells, GFP-tagged MICAL1 was mostly cytosolic and diffuse but was associated with dynamic tubules in 15%–20% of the cells (Fig. 2 G, Fig. S3 A, and Video 1), where it colocalized throughout time with GRAF1b/2 (Fig. 2 H, Fig. S3 B, and Video 2). Although MICAL1 PPAPP could sometimes be found on GRAF1b/2 tubules (Fig. S3 C), colocalization was significantly reduced (Fig. 2 I). MICAL1 antibodies did not label any clear structures in HeLa cells (Fig. 2 J) and were only faintly associated with GRAF1b/2 tubules (Fig. 2 K and Fig. S3 D). GFP-tagged WDR44, on the other hand, labeled a heterogeneous ensemble of dynamic tubules and vesicles but also, in ∼30% of the cells, peripheral amorphous patches (Fig. 2 G, Fig. S3 A, and Video 1). These patches were an artifact of protein overexpression, as endogenous WDR44 was only associated with puncta and tubules (Fig. 2 J and Fig. S3 E). In addition, unlike WDR44 tubules, they did not colocalize with GRAF1b/2 (Fig. 2 H, Fig. S3 F, and Video 3). In agreement with its failure to coimmunoprecipitate GRAF1b/2, WDR44 ΔPro did not colocalize with them (Fig. 2 I and Fig. S3 G). Endogenous GRAF2 was found on GFP-WDR44 tubules and vice versa (Fig. 2, K and L). The number of HeLa cells with endogenous WDR44 tubules increased upon overexpression of GRAF1b/2, but not MICAL1 (Fig. 2 M). Reciprocally, it was significantly decreased by down-regulation of GRAF2 expression, but not MICAL1 (Fig. 2 N and Fig. S3 H). The BAR domain of GRAF2 was necessary, as GRAF2 ΔBAR was diffuse and, when transfected in shGRAF2a-expressing cells, led to the association of RFP-WDR44 with puncta instead of long tubules (Fig. S3 I). In HeLa cells, the extent of endogenous WDR44 tubulation was thus directly related to the expression level of GRAF2.

**Video 1.** **GRAF1b, GRAF2, MICAL1, and WDR44 are cytosolic proteins associated with dynamic intracellular tubules.** HeLa cells were transfected with GRAF1b-GFP, GRAF2-GFP, GFP-MICAL1, or GFP-WDR44 and imaged with a confocal spinning disk. Images were captured at 5-s intervals. Movies are run here in parallel at seven frames per second. Scale bars: 10 µm.

**Video 2.** **GRAF2 and MICAL1 are associated with the same dynamic intracellular tubules.** HeLa cells were transfected with GRAF2-RFP and GFP-MICAL1 and imaged with a confocal spinning disk. Snapshots were captured at 5-s intervals and are shown here at seven frames per second. Boxed areas showing examples of newly formed tubules simultaneously positive for GRAF2-RFP and GFP-MICAL1 are magnified. Scale bar: 10 µm; scale bars of insets: 2 µm.

**Video 3.** **GRAF2 and WDR44 are associated with the same dynamic intracellular tubules.** HeLa cells were transfected with GRAF2-RFP and WDR44-GFP and imaged with a confocal spinning disk. Snapshots were captured at 5-s intervals and are shown here at seven frames per second. Boxed areas showing examples of newly formed tubules simultaneously positive for GRAF2-RFP and WDR44-GFP are magnified. Scale bar: 10 µm; scale bars of insets: 2 µm.

We have shown that WDR44 and MICAL1 bind the same domain of GRAF1b/2, suggesting that they may compete for GRAF1b/2 binding. In agreement, although WDR44 and MICAL1 were found on the same tubules, they were enriched on complementary segments (Fig. 2 O). Live imaging showed the initial formation of MICAL1-positive tubules to which WDR44 was later recruited (Video 4). These experiments show that WDR44 and MICAL1 are dynamically associated with the same intracellular tubules as GRAF1b/2 and suggest a temporal transition from GRAF1b/2–MICAL1 to GRAF1b/2–WDR44 complexes.

**Video 4.** **MICAL1 precedes WDR44 on intracellular tubules.** HeLa cells were transfected with RFP-MICAL1 and GFP-WDR44 and imaged with a confocal spinning disk. Snapshots were captured at 5-s intervals and are shown here at 3.5 frames per second. Boxed areas showing examples of newly formed RFP-MICAL1 tubules that then acquire GFP-WDR44 are magnified. Scale bar: 10 µm; scale bars of insets: 2 µm.

### MICAL1 connects GRAF1b/2 to Rab8 and Rab10

**Figure 3.**
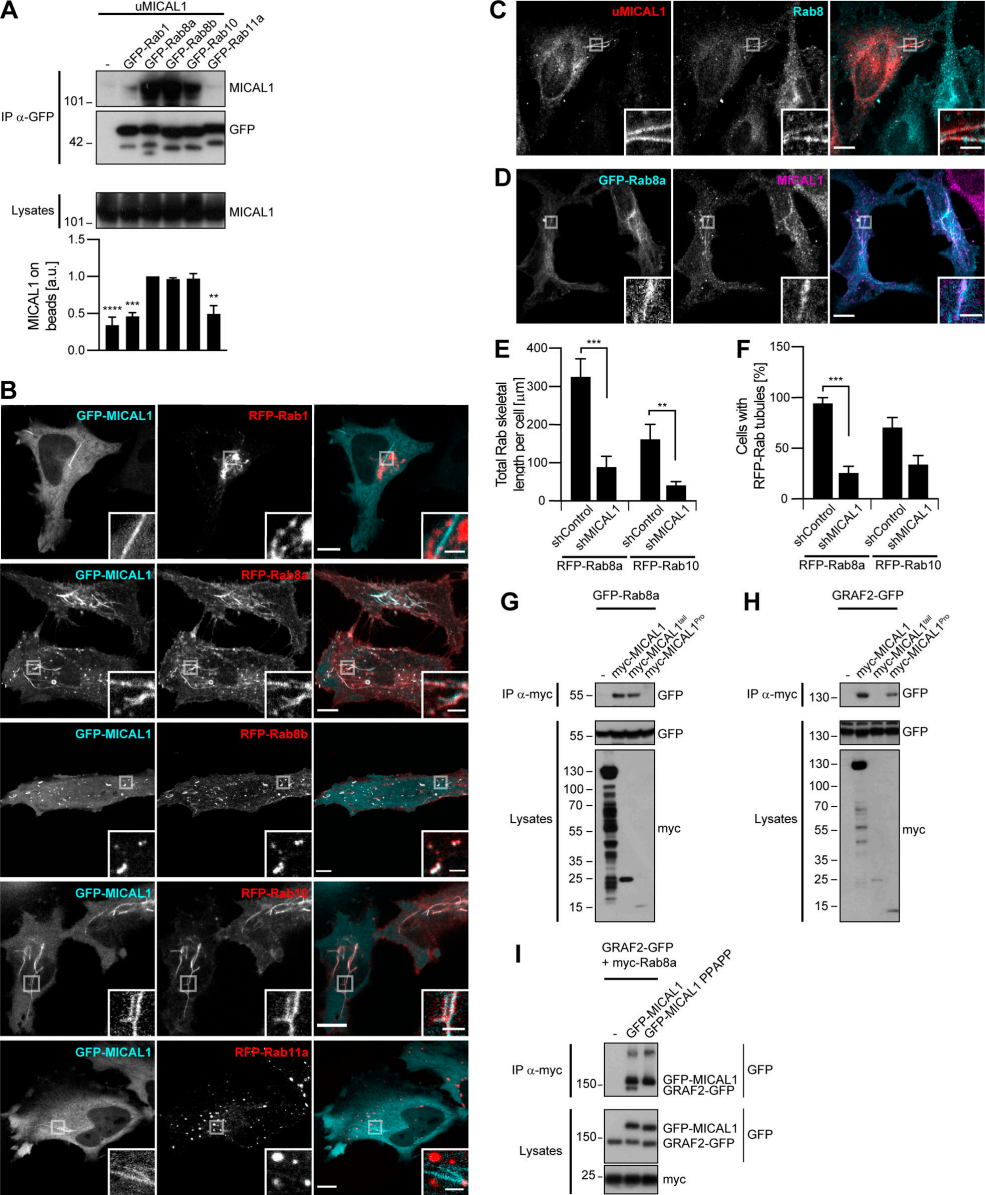
**MICAL1 connects GRAF1b/2 to Rab8 and Rab10. (A)** Immunoprecipitation (IP) of transfected 293T cells with α-GFP. The efficiency of uMICAL1 binding was quantified using coimmunoprecipitation with GFP-Rab8a as reference. *n* = 2–5. MICAL1 bound to Rab8a, Rab8b, and Rab10. **(B)** Confocal images of transfected HeLa cells. GFP-MICAL1 colocalized with RFP-Rab8a, Rab8b, and Rab10 but not Rab1 or Rab11a. **(C and D)** Confocal images of transfected HeLa cells stained with α-Rab8 or α-MICAL1. **(C)** Endogenous Rab8 was found on uMICAL1 tubules. **(D)** Endogenous MICAL1 was found on GFP-Rab8a tubules. **(E and F)** shControl or shMICAL1-expressing cells were transfected with RFP-Rab8a or Rab10. **(E)** Total skeletal length of RFP-Rab8a/10 structures per cell. *n* = 22–36 cells. **(F)** Proportion of cells with Rab8a/10–positive tubules. *n* = 3. **(G–I)** Immunoprecipitation (IP) of transfected 293T cells with α-myc–coated beads (G and H) or α-myc (I). **(G and H)** myc-MICAL1^tail^ only coimmunoprecipitated GFP-Rab8a; myc-MICAL1^Pro^ only coimmunoprecipitated GRAF2-GFP. **(I)** GRAF2-GFP was only coimmunoprecipitated with myc-Rab8a when GFP-MICAL1 was coexpressed, not GFP-MICAL1 PPAPP. **(A, E, and F)** Data are means ± SEM; **, P < 0.01; ***, P < 0.001; and ****, P < 0.0001. **(B–D)** Insets show magnifications of the boxed areas. Scale bars: 10 µm; scale bars of insets: 2 µm.

**Figure S4.**
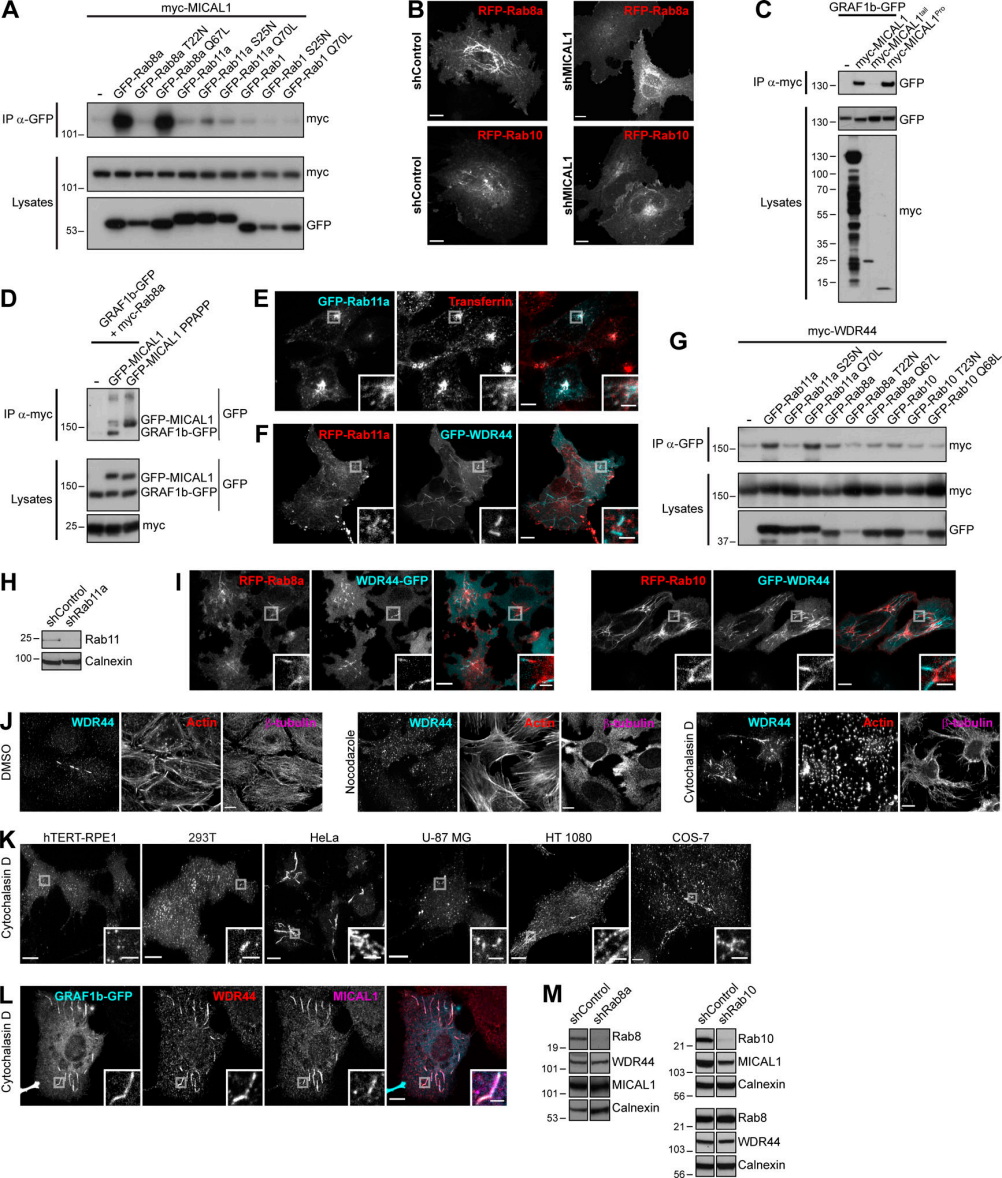
**MICAL1 and WDR44 connect GRAF1b/2 to Rab8, Rab10, and Rab11. (A, C, D, and G)** Immunoprecipitation (IP) of transfected 293T cells with α-GFP (A and G), α-myc–coated beads (C), or α-myc (D). **(A)** myc-MICAL1 was coimmunoprecipitated by Rab8a Q67L, but not by Rab8a T22N. **(B)** Confocal stacks of shControl or shMICAL1-expressing HeLa cells transfected with RFP-Rab8a or RFP-Rab10. **(C)** myc-MICAL1 and myc-MICAL1^Pro^ but not myc-MICAL1^tail^ coimmunoprecipitated GRAF1b-GFP. **(D)** GRAF1b-GFP was only coimmunoprecipitated with myc-Rab8a when GFP-MICAL1 was coexpressed, not GFP–MICAL1 PPAPP. **(E)** Confocal images of GFP-Rab11a–transfected HeLa cells incubated with Alexa Fluor 546–Transferrin (10 µg/ml, 30 min) showing colocalization. **(F)** Confocal images of transfected HeLa cells. RFP-Rab11a was found on GFP-WDR44–positive tubules, but GFP-WDR44 was not recruited to RFP-Rab11a–positive endosomes. **(G)** myc-WDR44 was coimmunoprecipitated by Rab11a and by its constitutively active mutant Rab11a Q70L, but not by the dominant negative mutant Rab11a S25N. **(H and M)** Western blot analysis of cell lysates from HeLa cells transfected with specific shRNA-encoding plasmids. Calnexin was used as loading control. **(H)** Rab11 was knocked down in shRab11a-transfected cells. **(I)** Confocal images of transfected HeLa cells showing RFP-tagged Rab8a/10 on the same intracellular tubules as GFP-tagged WDR44 but enriched on complementary segments of the tubules. **(J)** Confocal stacks of HeLa cells incubated with DMSO (vehicle, 2 h), Nocodazole (20 µg/ml, 2 h), or Cytochalasin D (0.5 µg/ml, 30 min) and stained with α-WDR44, α-β-tubulin, and Alexa Fluor 546–phalloidin. **(K)** Confocal stacks of hTERT-RPE1, 293T, HeLa, U-87 MG, HT 1080, and COS-7 cells incubated with Cytochalasin D (0.5 µg/ml, 30 min) and stained with α-WDR44. Cytochalasin D induced endogenous WDR44 tubules in HeLa, HT 1080, and COS-7 cells (compare with Fig. S3 E). **(L)** Confocal images of transfected HeLa cells incubated with Cytochalasin D (0.5 µg/ml, 30 min) and stained with α-WDR44 and α-MICAL1 showing colocalization with GRAF1b-GFP. **(M)** Rab8a and Rab10 were knocked down in shRab8a- and shRab10-transfected cells, respectively. In each case, the two parts of the blots are from the same membrane and correspond to identical exposure times. shRab10: the upper three blots and the lower three are from replicate membranes. **(B, E, F, I, and J–L)** Insets show magnifications of the boxed areas. Scale bars: 10 µm; scale bars of insets: 2 µm.

Based on the literature, MICAL1 may connect GRAF1b/2 to Rabs. To investigate this possibility, we first confirmed earlier reports that MICAL1 interacts with activated forms of Rab8a, Rab8b, and Rab10, but not Rab1 or Rab11a (Fukuda et al., 2008; Rai et al., 2016), by testing coimmunoprecipitation of the overexpressed proteins in 293T cells (Fig. 3 A and Fig. S4 A) and colocalization in HeLa cells (Fig. 3 B). Time-lapse imaging showed that MICAL1 was recruited simultaneously to Rab8a and Rab10 on newly formed tubules (Video 5 and Video 6). Endogenous Rab8 was found on overexpressed MICAL1 tubules and reciprocally (Fig. 3, C and D). MICAL1 knockdown significantly decreased the extent of tubulation associated with RFP-Rab8a and Rab10 (Fig. 3, E and F; and Fig. S4 B). This suggests a role for endogenous MICAL1 in the growth of Rab8/10 tubules. In agreement with recent data (Rai et al., 2016), a protein made of the last 167 aa of MICAL1, MICAL1^tail^, was sufficient to coimmunoprecipitate Rab8a (Fig. 3 G). The GRAF-binding domain of MICAL1 lies outside this region, as MICAL^tail^ did not coimmunoprecipitate GRAF1b/2, unlike MICAL1^Pro^ (aa 800–850), which contains the PPKPP motif (Fig. 3 H and Fig. S4 C). This suggests that MICAL1 may bind simultaneously to GRAFs and Rabs. In agreement, Rab8a coimmunoprecipitated GRAF1b/2 upon coexpression of MICAL1, but not MICAL1 PPAPP (Fig. 3 I and Fig. S4 D). MICAL1 can thus bridge GRAF1b/2 and Rab8a and, by extension, is expected to bridge GRAF1b/2 and Rab8b/10.

**Video 5.** **MICAL1 colocalizes with Rab10.** HeLa cells were transfected with RFP-Rab10 and GFP-MICAL1 and imaged with a confocal spinning disk. Snapshots were captured at 5-s intervals and are shown here at seven frames per second. Boxed areas showing examples of newly formed tubules simultaneously positive for RFP-Rab10 and GFP-MICAL1 are magnified. Scale bar: 10 µm; scale bars of insets: 2 µm.

**Video 6.** **MICAL1 colocalizes with Rab8a.** HeLa cells were transfected with RFP-Rab8a and GFP-MICAL1 and imaged with a confocal spinning disk. Snapshots were captured at 5-s intervals and are shown here at seven frames per second. Boxed areas showing examples of newly formed tubules simultaneously positive for RFP-Rab8a and GFP-MICAL1 are magnified. Scale bar: 10 µm; scale bars of insets: 2 µm.

### WDR44 tubules form in a Rab11-, Rab8-, and Rab10-dependent manner and are induced by Cytochalasin D

**Figure 4.**
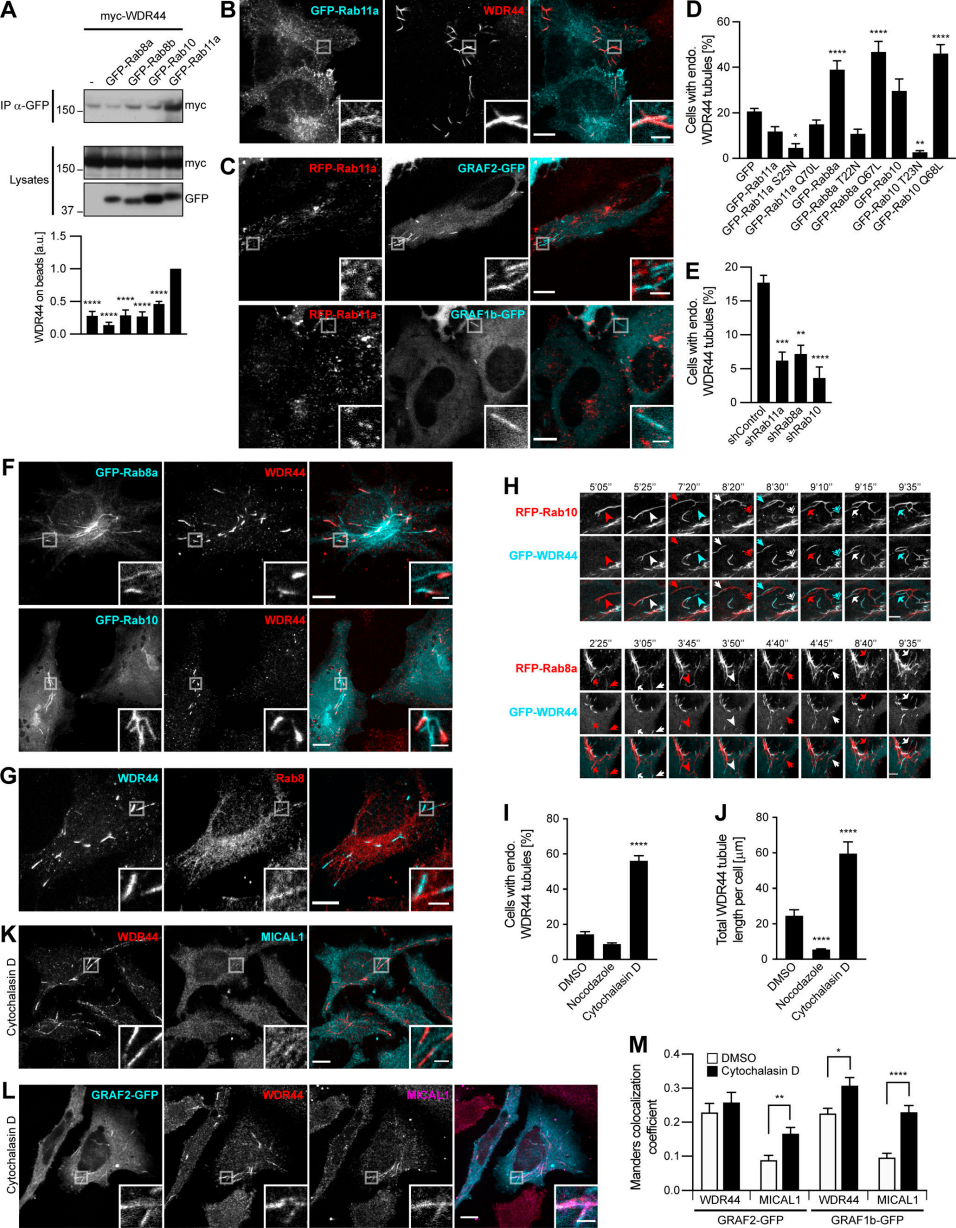
**WDR44 tubules form in a Rab11-, Rab8-, and Rab10-dependent manner and are induced by Cytochalasin D**. **(A)** Immunoprecipitation (IP) of transfected 293T cells with α-GFP. The efficiency of myc-WDR44 binding was quantified using coimmunoprecipitation with GFP-Rab11a as reference. *n* = 3–5. myc-WDR44 only bound to Rab11a. **(B and C)** Confocal images of transfected HeLa cells. **(B)** Cells were stained with α-WDR44. GFP-Rab11a was found on endogenous WDR44 tubules, but WDR44 was absent from GFP-Rab11a–positive endosomes. **(C)** RFP-Rab11a was absent from GRAF1b/2–positive tubules. **(D and E)** Percentage of HeLa cells with endogenous (endo.) WDR44 tubules. **(D)** *n* = 3–6. **(E)** *n* = 3. **(F)** Confocal images of transfected HeLa cells stained with α-WDR44 showing endogenous WDR44 on the end of GFP-Rab8a and GFP-Rab10 tubules. **(G)** Confocal images of untransfected HeLa cells stained with α-WDR44 and α-Rab8 showing colocalization on tubules. **(H)** Confocal images of transfected HeLa cells. Snapshots were taken every 5 s. Regions with dynamic tubules are shown at relevant time points. Top: Stills from Video 7. Arrows/arrowheads point at tubules that were first positive for Rab10 (red), then acquired WDR44 (white), and finally lost Rab10, leaving only WDR44 (cyan). Bottom: Stills from Video 8. Arrows/arrowheads point at newly formed Rab8a tubules (red) that then acquired WDR44 (white). Rab8a did not clearly dissociate from WDR44 tubules. Scale bars: 5 µm. **(I and J)** HeLa cells were incubated with DMSO (vehicle, 2 h), Nocodazole (20 µg/ml, 2 h), or Cytochalasin D (0.5 µg/ml, 30 min) and stained with α-WDR44. **(I)** Percentage of cells with endogenous (endo.) WDR44 tubules. *n* = 4–6. **(J)** Total length of WDR44 tubules per cell. *n* = 70–100 cells. **(K and L)** Confocal images of untransfected (K) or transfected (L) HeLa cells incubated with Cytochalasin D (0.5 µg/ml, 30 min) and stained with α-WDR44 and α-MICAL1. **(M)** Manders colocalization coefficients for endogenous WDR44 and MICAL1 on GRAF1b/2–GFP structures in DMSO or Cytochalasin D–treated cells. *n* = 20–33 cells. **(A, D, E, I, J, and M) **Data are means ± SEM; *, P < 0.05; **, P < 0.01; ***, P < 0.001; and ****, P < 0.0001. **(B, C, F, G, K, and L)** Insets show magnifications of the boxed areas. Scale bars: 10 µm; scale bars of insets: 2 µm.

WDR44 was originally identified as a Rab11-binding protein (Zeng et al., 1999; Mammoto et al., 1999). In agreement, Rab11a, but not Rab8a/b or Rab10, coimmunoprecipitated WDR44 when overexpressed in 293T cells (Fig. 4 A). When transfected in HeLa cells, Rab11a was principally associated with puncta that colocalized with Transferrin-positive endosomes (Fig. S4 E). Rab11a was also found on endogenous WDR44 tubules, but WDR44 was not recruited to Rab11a-positive endosomes (Fig. 4 B and Fig. S4 F). In addition, neither GRAF1b/2 nor MICAL1 colocalized with Rab11a (Fig. 4 C and Fig. 3 B). Whereas overexpression of Rab11a or of the constitutively active mutant Rab11a Q70L had no significant effect on WDR44 tubulation, overexpression of the dominant negative mutant Rab11a S25N, which did not bind WDR44 (Fig. S4 G), decreased it (Fig. 4 D). Down-regulation of Rab11a expression with transfection of the shRNA-expressing plasmid shRab11a (Fig. S4 H) also led to a significant decrease in WDR44 tubulation (Fig. 4 E). Therefore, whereas Rab11a is not a stable component of the MICAL1/GRAF/WDR44 tubules, its activation is important for WDR44-positive tubules to form.

Even though WDR44 did not bind to Rab8 or Rab10, it displayed a contiguous colocalization with them on intracellular tubules (Fig. 4, F and G; and Fig. S4 I). Imaging of live cells showed that WDR44 was recruited to preexisting Rab8a- and Rab10-positive tubules (Fig. 4 H, Video 7, and Video 8). Rab10 then dissociated from WDR44-positive tubules, but Rab8a remained. By similarity to Rab8 and Rab10 (Etoh and Fukuda, 2019; Radhakrishna and Donaldson, 1997; Hattula et al., 2006), WDR44 tubules were destabilized by incubation of cells with Nocodazole, which disrupts microtubules, and induced by incubation of cells with Cytochalasin D, which fragments the actin cytoskeleton (Fig. 4, I and J; and Fig. S4, J and K). Cytochalasin D also improved detection of endogenous MICAL1 along WDR44-positive tubules (Fig. 4 K) and on GRAF1b/2 tubules (Fig. 4, L and M; and Fig. S4 L). Overexpression of Rab8a and of the constitutively active mutants Rab8a Q67L and Rab10 Q68L increased WDR44 tubulation (Fig. 4 D). Conversely, overexpression of the dominant negative mutant Rab10 T23N decreased it. These experiments suggest that Rab8 and Rab10 actively promote the formation of intracellular tubules, to which WDR44 is recruited. In agreement, knocking down Rab8a or Rab10 inhibited endogenous WDR44 tubulation (Fig. 4 E; and Fig. S4 M). Therefore, although WDR44 only binds to Rab11a, WDR44 tubule formation is differentially controlled by the three GTPases Rab8, Rab10, and Rab11. Our experiments also show that the formation, stability, and turnover of MICAL1-GRAF-WDR44 complexes and associated membrane tubules are intricately connected to the status and dynamics of the actin and microtubule cytoskeleton.

**Video 7.** **Sequential association of Rab10 and WDR44 on intracellular tubules.** HeLa cells were transfected with RFP-Rab10 and GFP-WDR44 and imaged with a confocal spinning disk. Snapshots were captured at 5-s intervals and are shown here at seven frames per second. Boxed area corresponds to region magnified in Fig. 4 H. Scale bar: 10 µm.

**Video 8.** **Sequential association of Rab8a and WDR44 on intracellular tubules.** HeLa cells were transfected with RFP-Rab8a and GFP-WDR44 and imaged with a confocal spinning disk. Snapshots were captured at 5-s intervals and are shown here at seven frames per second. Boxed area corresponds to region magnified in Fig. 4 H. Scale bar: 10 µm.

### MICAL1 G3W, WDR44 ΔC, and GRAF1/2 BAR-PH are dominant negative mutants interfering with Rab8/10–mediated trafficking and WDR44 tubulation

**Figure 5.**
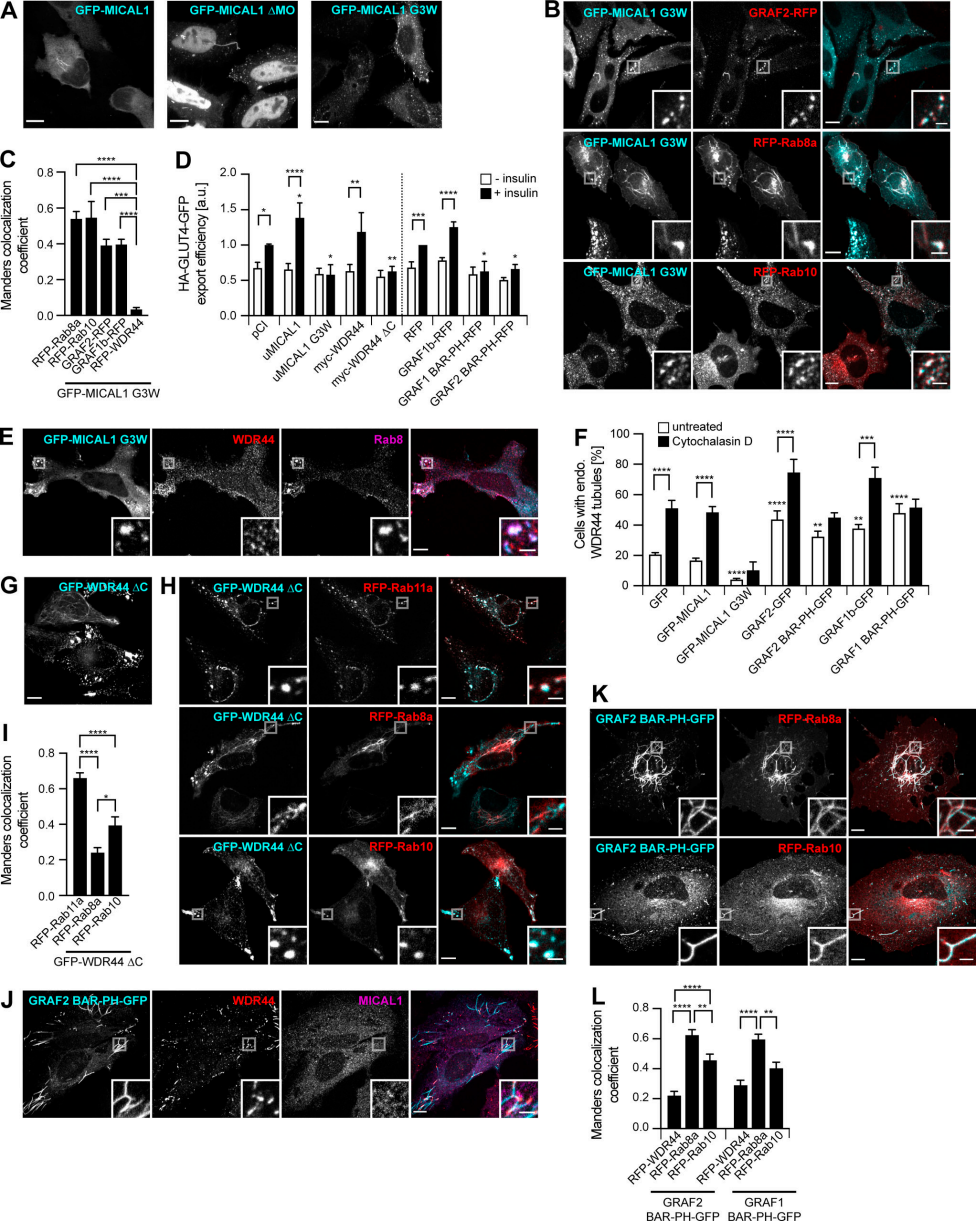
**MICAL1 G3W, WDR44 ΔC, and GRAF1/2 BAR-PH are dominant negative mutants. (A)** Confocal stacks of transfected HeLa cells. GFP–MICAL1 ΔMO and GFP–MICAL1 G3W were associated with many puncta and tubules. **(B)** Confocal images of transfected HeLa cells. GFP–MICAL1 G3W colocalized with RFP-tagged GRAF2, Rab8a, and Rab10 on puncta and tubules. **(C)** Manders colocalization coefficients for the indicated proteins on GFP–MICAL1 G3W structures. *n* = 9–24 cells. **(D)** HA-GLUT4-GFP export in transfected 3T3-L1 adipocytes under resting conditions or after the addition of insulin (10 µg/ml, 30 min). *n* = 4–12. **(E)** Confocal images of transfected HeLa cells stained with α-WDR44 and α-Rab8. Unlike WDR44, endogenous Rab8 colocalized with GFP–MICAL1 G3W. **(F)** Percentage of transfected HeLa cells left untreated or after incubation with Cytochalasin D (0.5 µg/ml, 30 min), with endogenous (endo.) WDR44 tubules. *n* = 3–8. **(G)** Confocal stack of HeLa cells transfected with GFP–WDR44 ΔC showing its association with large peripheral patches but also, in a few cells, with a thin reticular network. **(H)** Confocal images of transfected HeLa cells. GFP–WDR44 ΔC recruited RFP-tagged Rab11a and Rab10 to amorphous peripheral patches and colocalized with Rab8a and Rab10 on intracellular puncta and tubules. **(I)** Manders colocalization coefficients for the indicated proteins on GFP–WDR44 ΔC structures. *n* = 20–31 cells. **(J)** Confocal images of transfected HeLa cells stained with α-MICAL1 and α-WDR44. Unlike MICAL1, WDR44 was recruited to sections of GRAF2 BAR-PH–positive membranes. **(K)** Confocal images of transfected HeLa cells. GRAF2 BAR-PH–GFP colocalized with RFP-tagged Rab8a and Rab10. **(L)** Manders colocalization coefficients for the indicated proteins on GRAF1/2 BAR-PH–GFP structures. *n* = 21–26 cells. **(C, D, F, I, and L)** Data are means ± SEM; *, P < 0.05; **, P < 0.01; ***, P < 0.001; and ****, P < 0.0001. **(A, B, E, G, H, J, and K)** Insets show magnifications of the boxed areas. Scale bars: 10 µm; scale bars of insets: 2 µm.

**Figure S5.**
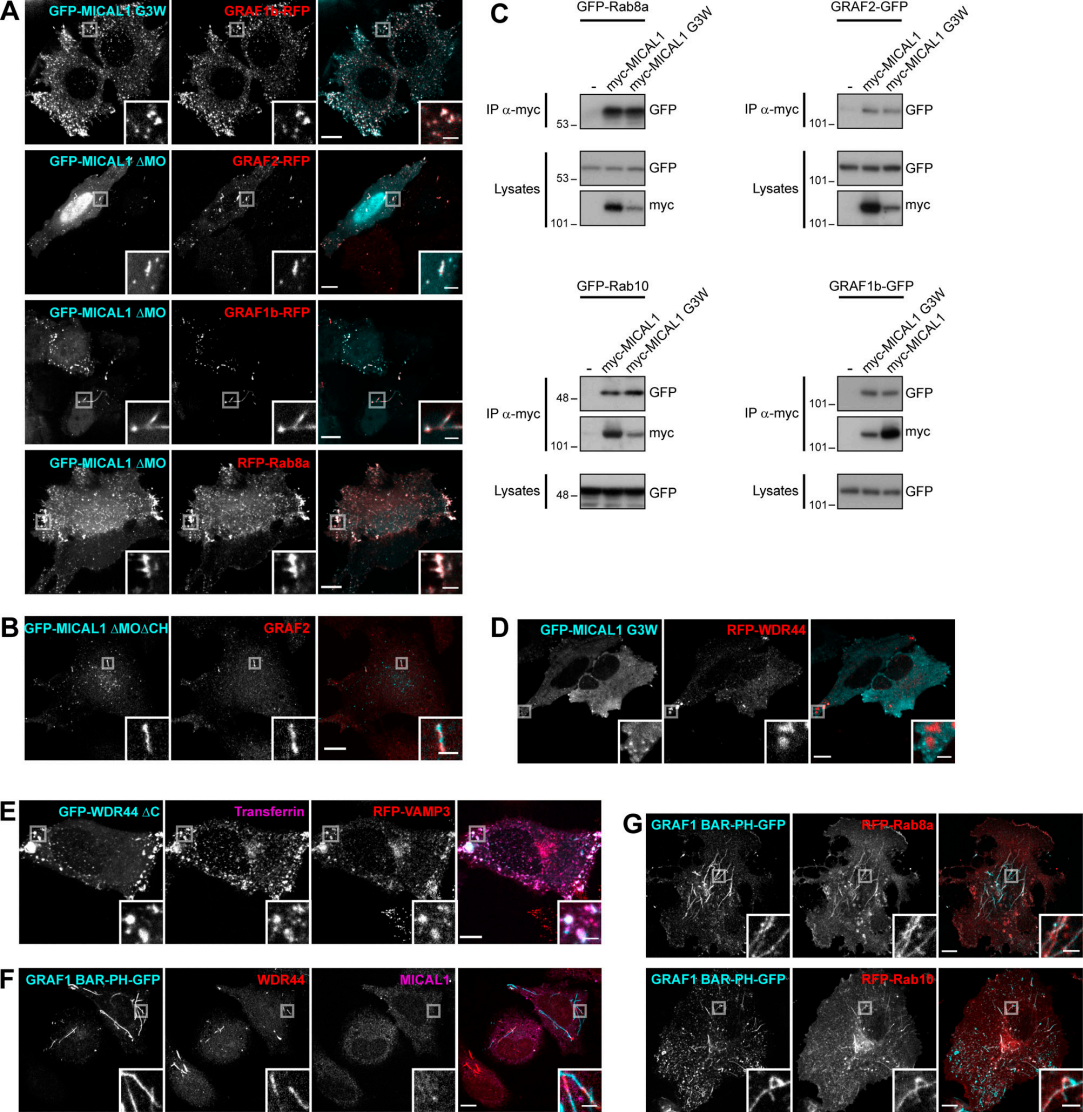
**MICAL1 G3W, WDR44 ΔC, and GRAF1/2 BAR-PH are dominant negative mutants interfering with Rab8/10–mediated trafficking and WDR44 tubulation.** **(A)** Confocal images of transfected HeLa cells. GFP**–**MICAL1 G3W colocalized with GRAF1b-RFP; GFP**–**MICAL1 ΔMO colocalized with RFP-tagged GRAF2, GRAF1b, and Rab8a on intracellular puncta and tubules. **(B)** Confocal images of transfected HeLa cells stained with α-GRAF2. Endogenous GRAF2 was found on GFP**–**MICAL1 ΔMOΔCH puncta and tubules. GFP**–**MICAL1 ΔMOΔCH had a lower nuclear background than GFP**–**MICAL1 ΔMO. **(C)** Immunoprecipitation (IP) of transfected 293T cells with α-myc. GFP-tagged Rab8a, Rab10, GRAF2, and GRAF1b were coimmunoprecipitated by myc**–**MICAL1 G3W as well as by myc-MICAL1. **(D)** Confocal images of transfected HeLa cells. RFP-WDR44 did not colocalize with GFP**–**MICAL1 G3W. **(E)** Confocal images of HeLa cells cotransfected with GFP**–**WDR44 ΔC and RFP-VAMP3 and incubated with Alexa Fluor 647**–**Transferrin (10 µg/ml, 1 h). The three proteins colocalized on intracellular peripheral patches. **(F)** Confocal images of transfected HeLa cells stained with α-WDR44 and α-MICAL1. Unlike MICAL1, endogenous WDR44 was found on GRAF1 BAR-PH**–**GFP puncta and tubules. **(G)** Confocal images of transfected HeLa cells. GRAF1 BAR-PH**–**GFP colocalized with RFP-tagged Rab8a and Rab10 on intracellular tubules and puncta. **(A, B, and D–G)** Insets show magnifications of the boxed areas. Scale bars: 10 µm; scale bars of insets: 2 µm.

The MICAL family of proteins is thought to coordinate Rab binding or receptor activation with rearrangements of the cytoskeleton (Giridharan and Caplan, 2014; Frémont et al., 2017). MICAL1–3 have a MonoOxygenase (MO) domain that can oxidize proteins (Vitali et al., 2016; Schmidt et al., 2008). So far, two MICAL substrates have been identified, Actin and CRMP2, whose oxidation results in destabilization of F-actin and microtubules, respectively (Lee et al., 2013; Morinaka et al., 2011). Deletion (MICAL1 ΔMO) or inactivation (MICAL1 G3W; Terman et al., 2002) of the MO domain of MICAL1 increased its association with intracellular puncta and tubules (Fig. 5 A). These structures colocalized with GRAF1b/2, Rab8a, and Rab10 (Fig. 5, B and C; and Fig. S5, A and B), which were still coimmunoprecipitated by MICAL1 G3W (Fig. S5 C). Since the subcellular distribution of Rab8/10 was dramatically altered by MICAL1 G3W (compare Rab8a/10 in Fig. 5 B with Fig. 4 F), we tested whether overexpression of MICAL1 G3W interfered with their function. Indeed, MICAL1 G3W blocked insulin-stimulated export of GLUT4 (Fig. 5 D), which is dependent on Rab10 in adipocytes (Sano et al., 2008). In addition, whereas MICAL1 G3W did not colocalize with WDR44 (Fig. 5, C and E; and Fig. S5 D), overexpression of MICAL1 G3W decreased endogenous WDR44 tubules and prevented their induction by Cytochalasin D (Fig. 5 F). This suggests that the MO activity of MICAL1 promotes its dissociation from intracellular membranes and the turnover of its complex with GRAF2. This would limit its effects to a local destabilization of the cytoskeleton that could facilitate the recruitment of WDR44 by GRAF2 but still allow the elongation of WDR44 tubules along intact microtubules. MICAL1 G3W is thus a dominant negative mutant that interferes with Rab10- (and presumably Rab8-) mediated trafficking and with WDR44 tubule formation.

By analogy, we then looked for a WDR44 mutant that might interfere with Rab-mediated trafficking. WDR44(1–504) was reported to inhibit Rab11-mediated recycling of the Transferrin receptor (Zeng et al., 1999). In agreement, WDR44 ΔC was mostly found on peripheral patches (Fig. 5 G), where it recruited Rab11a (Fig. 5, H and I) and colocalized with internalized Transferrin and the recycling endosome marker VAMP3 (Fig. S5 E). Unlike Rab10, Rab8a was not found on patches of WDR44 ΔC; but as WDR44 ΔC colocalized with both of them on cytoplasmic tubules and puncta (Fig. 5, H and I), it may still interfere with their functioning. In agreement, WDR44 ΔC inhibited insulin-stimulated export of GLUT4 (Fig. 5 D). WDR44 ΔC is thus a dominant negative mutant that interferes not only with Rab11-mediated recycling but also with Rab10- (and presumably Rab8-) dependent trafficking.

We have shown that WDR44 and MICAL1 bind to the SH3 domain of GRAF1/2. But even though the membrane-binding regions of GRAF1/2 (GRAF1/2 BAR-PH) did not colocalize with endogenous MICAL1, endogenous WDR44 was found on the same structures (Fig. 5 J and Fig. S5 F). Live imaging showed that these tubules were relatively immobile (Video 9). By similarity to the full-length proteins, GRAF1/2 BAR-PH induced endogenous WDR44 tubules (Fig. 5 F). Cytochalasin D, however, did not further stimulate it. This suggests that overexpression of GRAF1/2 BAR-PH interfered with the normal dynamics of WDR44 tubules. In addition, GRAF1/2 BAR-PH colocalized with Rab8a and Rab10 on puncta and tubules (Fig. 5, K and L; and Fig. S5 G) and interfered with insulin-stimulated export of GLUT4 (Fig. 5 D). This also indicates that the intrinsic characteristics of Rab8a/10 tubules are sufficient to recruit the membrane-binding region of GRAF1/2.

**Video 9.** **Dynamics of GRAF2 BAR-PH–associated tubules.** HeLa cells were transfected with GRAF2 BAR-PH–GFP and RFP-WDR44 and imaged with a confocal spinning disk. Snapshots were captured at 5-s intervals and are shown here at seven frames per second. Whereas GRAF2 BAR-PH–GFP was associated with many dynamic puncta, which could result from mistargeting to Caveolin- and Flotilin-associated structures (Lundmark et al., 2008), GRAF2 BAR-PH-GFP–associated tubules were more static than the tubules found with the full-length proteins. Scale bar: 10 µm.

MICAL1 G3W, WDR44 ΔC, GRAF2 BAR-PH, and GRAF1 BAR-PH are thus four dominant negative proteins affecting the normal functioning of different components of Rab8/10/11– and MICAL1/GRAF/WDR44–mediated trafficking.

### WDR44 tubules are in close contact with the ER via binding to VAPA/B

**Figure 6.**
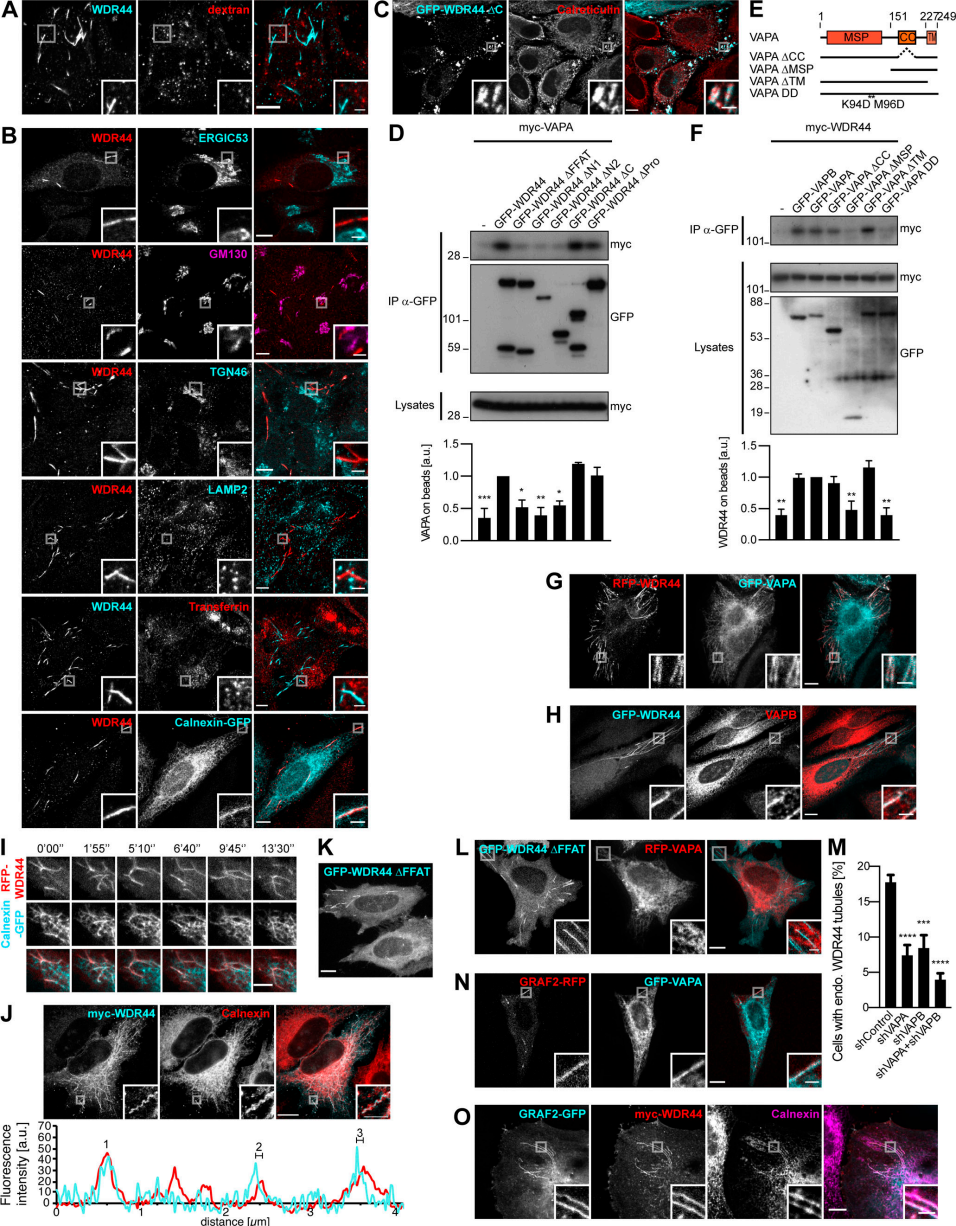
**WDR44 tubules are in close contact with the ER via binding to VAPA/B. (A)** Confocal images of HeLa cells incubated with 10 kD Alexa Fluor 546–dextran (5 mg/ml, 30 min) and stained with α-WDR44. There was no colocalization. **(B)** Confocal images of HeLa cells stained with α-WDR44 and α-ERGIC53, α-GM130, α-TGN46, or α-LAMP2. For visualization of recycling endosomes, cells were incubated with Alexa Fluor 546–Transferrin (10 µg/ml, 1 h); for the ER, cells were transfected with Calnexin-GFP. WDR44 tubules only colocalized with Calnexin-GFP. **(C)** Confocal images of transfected HeLa cells stained with α-Calreticulin, which colocalized with GFP–WDR44 ΔC patches. **(D and F)** Immunoprecipitation (IP) of transfected 293T cells with α-GFP. The efficiency of binding of myc-VAPA (D) and myc-WDR44 (F) was quantified using binding to GFP-WDR44 (D) or to GFP-VAPA (F) as reference. *n* = 3 or 4. **(D)** myc-VAPA was coimmunoprecipitated by GFP-WDR44, but not by mutants lacking the first 14 aa of the protein. **(E)** Schematic representation of human VAPA and of the mutants used. VAPA and VAPB have a cytoplasmic Major Sperm Protein (MSP) domain, a coiled coil (CC) region, and a transmembrane (TM) tail for anchoring in the ER. **(F)** myc-WDR44 was coimmunoprecipitated by GFP-tagged VAPA/B, but not by VAPA ΔMSP or VAPA DD. **(G and H)** Confocal images of transfected HeLa cells showing colocalization of RFP-WDR44 tubules with GFP-VAPA (G) and with endogenous VAPB (H). **(I)** Confocal images of live HeLa cells. Snapshots were taken every 5 s and are shown at relevant time points. RFP-WDR44 and Calnexin-GFP tubules were closely associated throughout time. Scale bars: 5 µm. **(J)** STED images of myc-WDR44–transfected HeLa cells stained with α-myc and α-Calnexin, and fluorescence intensity profiles along the line drawn, showing that the myc-WDR44 (red line) and Calnexin (cyan line) peaks were superimposed on tubule 1 but shifted by ∼80 nm on tubules 2 and 3. **(K)** Confocal stack of transfected HeLa cells. GFP-WDR44 ΔFFAT was associated with tubules. **(L)** Confocal images of transfected HeLa cells. GFP-WDR44 ΔFFAT tubules did not colocalize with RFP-VAPA. **(M)** Percentage of shRNA-expressing HeLa cells with endogenous (endo.) WDR44 tubules. *n* = 5–8. **(N)** Confocal images of transfected HeLa cells showing colocalization of GRAF2-RFP tubules with GFP-VAPA. **(O)** Confocal images of transfected HeLa cells stained with α-myc and α-Calnexin. When myc-WDR44 was coexpressed, GRAF2-GFP–positive tubules colocalized with an endogenous ER marker. **(D, F, and M) **Data are means ± SEM; *, P < 0.05; **, P < 0.01; ***, P < 0.001; and ****, P < 0.0001. **(A–C, G, H, J–L, N, and O)** Insets show magnifications of the boxed areas. Scale bars: 10 µm; scale bars of insets: 2 µm.

**Figure S6.**
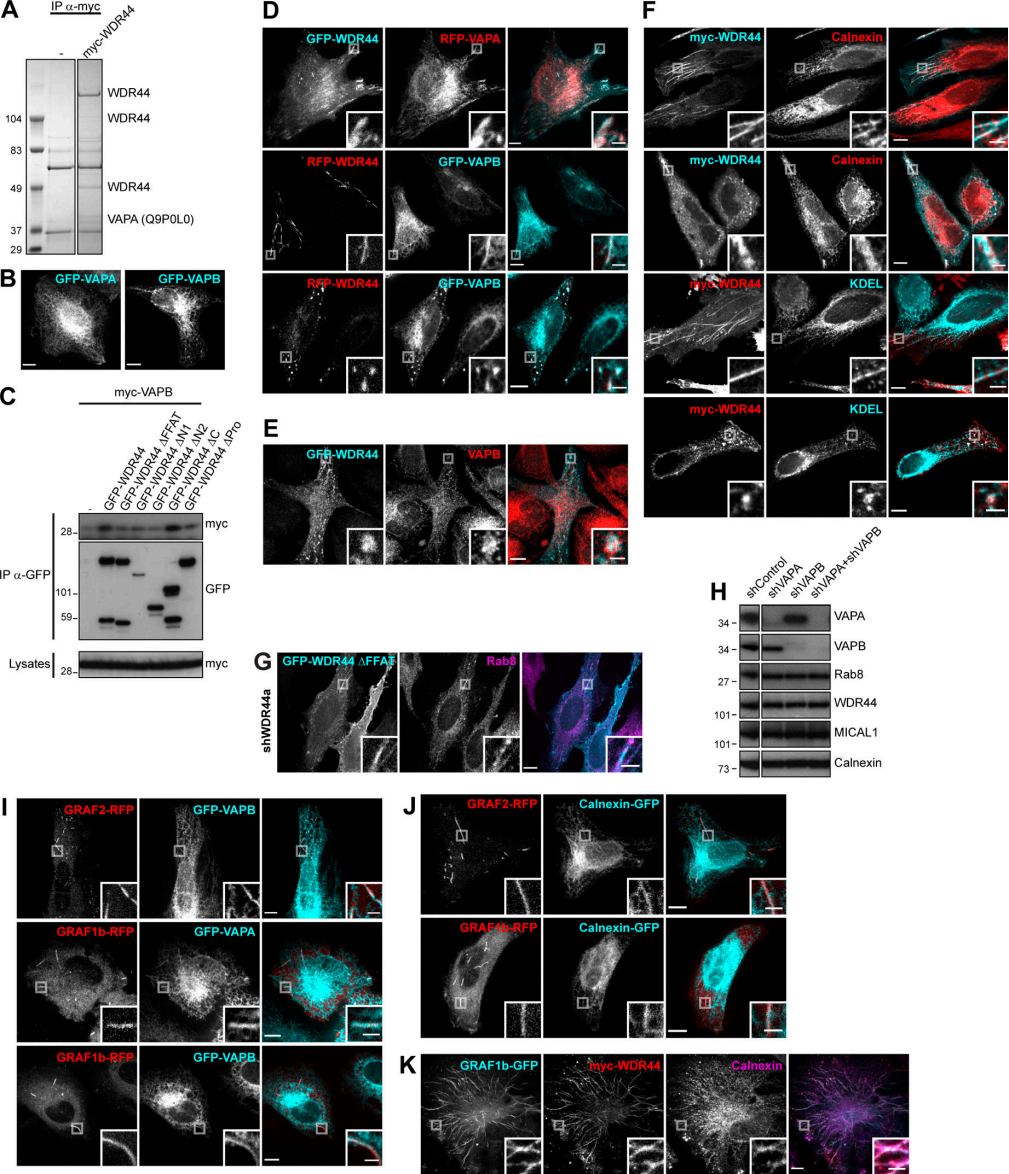
**WDR44 tubules are in close contact with the ER via binding to VAPA/B. (A)** Immunoprecipitation (IP) of endogenous proteins from 293T cells, untransfected or expressing myc-WDR44, with α-myc. Bound proteins were analyzed by electrophoresis and Coomassie staining. VAPA (Uniprot accession no. Q9P0L0) was identified by LC-MS/MS. **(B)** Confocal images of transfected HeLa cells showing GFP-VAPA or GFP-VAPB in reticular ER. **(C)** Immunoprecipitation (IP) of transfected 293T cells with α-GFP. myc-VAPB was coimmunoprecipitated by GFP-WDR44 but not by mutants lacking the first 14 aa of the protein. **(D)** Confocal images of transfected HeLa cells showing colocalization of WDR44 and VAPA/B on tubules and on peripheral patches. **(E)** Confocal images of transfected HeLa cells stained with α-VAPB showing colocalization of GFP-WDR44 patches and endogenous VAPB. **(F)** Confocal images of transfected HeLa cells stained with α-myc and α-Calnexin or α-KDEL. Myc-WDR44 tubules and patches colocalized with Calnexin, an ER transmembrane protein, and α-KDEL, which recognizes luminal ER proteins containing the ER retention signal KDEL. **(G)** Confocal images of shWDR44a-expressing HeLa cells transfected with GFP-WDR44 ΔFFAT and stained with α-Rab8. GFP**–**WDR44 ΔFFAT was associated with Rab8-positive tubules. **(H)** Western blot analysis of an equal protein amount of shRNA-expressing HeLa cell lysates showing specific knockdown of VAPA in shVAPA- and of VAPB in shVAPB-transfected cells. Calnexin was used as loading control. The two parts of the blots were from the same membrane and correspond to identical exposure times. The membrane was initially probed with α-WDR44 and α-VAPB, followed by α-MICAL1 and α-VAPA, and finally α-Calnexin and α-Rab8. **(I and J)** Confocal images of transfected HeLa cells. GRAF1b/2**–**RFP tubules colocalized with GFP-VAPA/B (I) but rarely with Calnexin-GFP (J). **(K)** Confocal images of transfected HeLa cells stained with α-myc and α-Calnexin. When WDR44 was coexpressed, GRAF1b tubules colocalized with an endogenous ER marker. **(B, D–G, and I–K)** Insets show magnifications of the boxed areas. Scale bars: 10 µm; scale bars of insets: 2 µm.

To gain more insight into the identity of MICAL1/GRAF/WDR44 tubules, we first examined colocalization of endogenous WDR44 with dextran as a marker of clathrin-independent endocytosis. There was none (Fig. 6 A). Endogenous WDR44 tubules also did not colocalize with markers of the ERGIC (ERGIC53), Golgi (GM130), TGN (TGN46), lysosomes (LAMP2), or recycling endosomes (internalized Transferrin; Fig. 6 B). They were, however, often aligned with markers of the ER (Calnexin-GFP), and the patches associated with WDR44 ΔC overlapped with endogenous Calreticulin, an ER protein (Fig. 6 C). We searched for binding partners of WDR44 ΔC by coimmunoprecipitation of endogenous proteins after overexpression in 293T cells and identified VAPA (Fig. S6 A). VAPA and its closely related homologue VAPB are transmembrane proteins of the ER (Fig. S6 B). They act as receptors for many cytoplasmic proteins, which often bind the MSP domain of VAPs via an FFAT motif (EFFDAxE; Murphy and Levine, 2016). Indeed, WDR44 has an FFAT sequence (Fig. 2 B), and WDR44 ΔFFAT was not coimmunoprecipitated by VAPA (Fig. 6 D) or VAPB (Fig. S6 C). Reciprocally, VAPA ΔMSP and VAPA DD, with two point mutations in its FFAT-binding site (K94D M96D; Kaiser et al., 2005), were not coimmunoprecipitated by WDR44 (Fig. 6, E and F).

In transfected HeLa cells, WDR44 tubules and patches colocalized with VAPA and VAPB (Fig. 6 G and Fig. S6 D). WDR44 tubules also colocalized with endogenous VAPB (Fig. 6 H and Fig. S6 E) and other endogenous markers of the ER (Fig. S6 F). The dynamics of WDR44 tubules closely followed the dynamics of the ER (Fig. 6 I). Super-resolution microscopy however showed that even though WDR44 and endogenous ER markers were in close apposition, their fluorescence intensity maxima were sometimes slightly shifted from one another (Fig. 6 J). In addition, WDR44 ΔFFAT was still found on tubules (Fig. 6 K and Fig. S6 G), but it failed to colocalize with VAPA (Fig. 6 L). Nevertheless, when VAPA and VAPB were knocked down together, endogenous WDR44 tubules were reduced (Fig. 6 M and Fig. S6 H). Therefore, VAPA/B contribute to WDR44 tubule formation and/or stabilization.

By similarly with WDR44, GRAF1b/2 tubules colocalized with VAPA and VAPB (Fig. 6 N and Fig. S6 I). Colocalization with Calnexin-GFP was however rare (Fig. S6 J), and colocalization with endogenous ER markers only became clear when WDR44 was coexpressed (Fig. 6 O and Fig. S6 K). Together, these experiments demonstrate that via VAP binding, WDR44 mediates close apposition of GRAF1b/2–positive tubules with the ER.

### GRAF/WDR44 label a subset of tubular endosomes but are not involved in recycling

**Figure 7.**
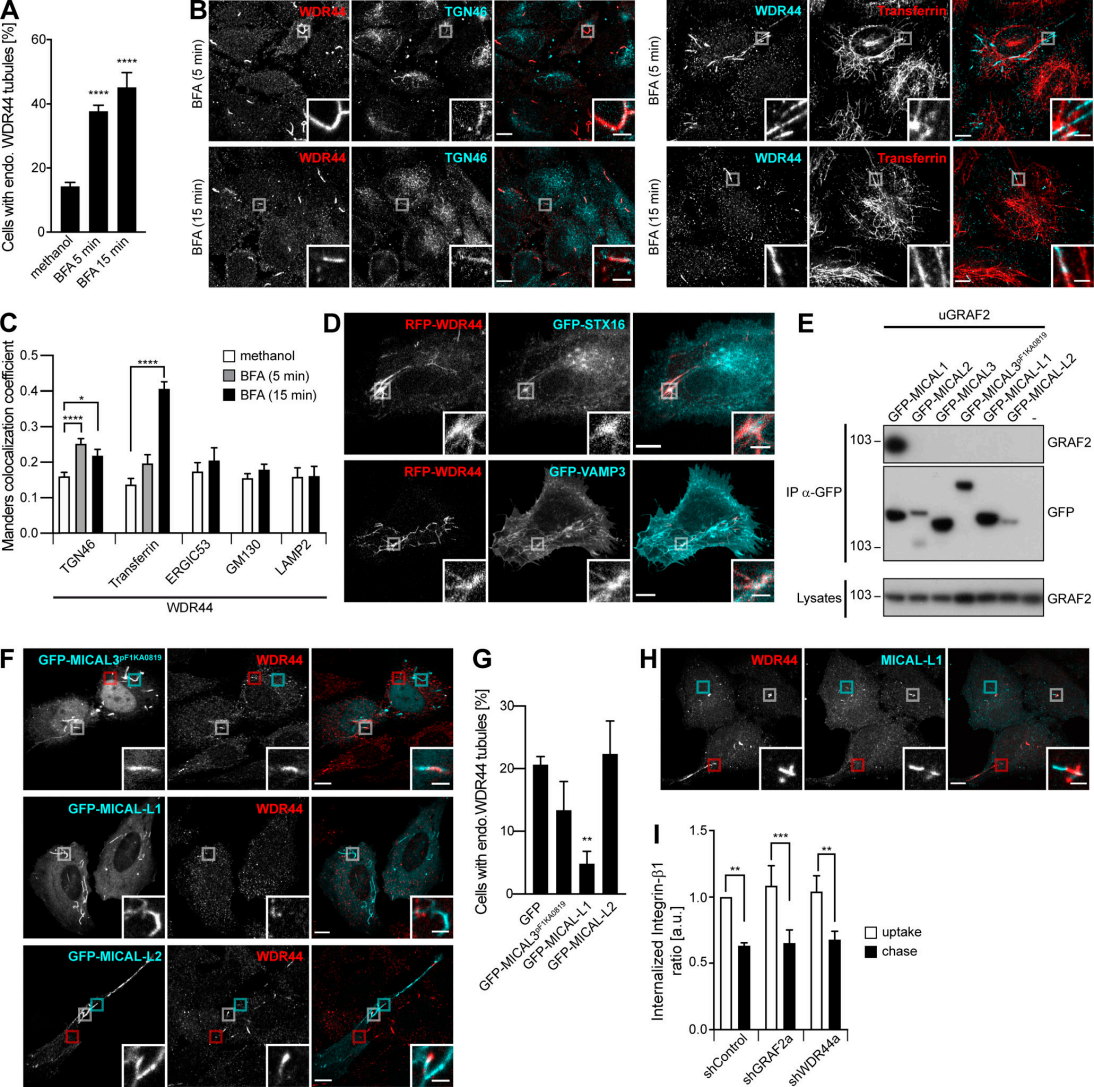
**GRAF/WDR44 label a subset of tubular endosomes. (A–C)** HeLa cells were incubated with methanol (vehicle) or BFA (5 µg/ml) for 5 or 15 min. **(A)** Percentage of cells with endogenous (endo.) WDR44 tubules. *n* = 7**–**25. **(B)** Confocal images of cells stained with α-WDR44 and α-TGN46 or preincubated with Alexa Fluor 546**–**Transferrin (10 µg/ml, 1 h). **(C)** Manders colocalization coefficients for the indicated proteins with endogenous WDR44 structures. *n* = 10**–**50 cells. **(D)** Confocal images of transfected HeLa cells showing colocalization of RFP-WDR44 with GFP-STX16 and GFP-VAMP3. **(E)** Immunoprecipitation (IP) of transfected 293T cells with α-GFP. uGRAF2 was coimmunoprecipitated by GFP-MICAL1, but not by any other member of the MICAL family. **(F)** Confocal images of transfected HeLa cells stained with α-WDR44. In the case of MICAL3^pF1KA0819^ and MICAL-L2, boxed areas show tubules positive only for WDR44 (red), only for MICAL3^pF1KA0819^/MICAL-L2 (cyan), or shared by the two proteins (white). **(G)** Percentage of transfected HeLa cells with endogenous (endo.) WDR44 tubules. *n* = 4. **(H)** Confocal images of untransfected HeLa cells stained with α-WDR44 and α-MICAL-L1. Boxed areas show tubules positive only for WDR44 (red), only for MICAL-L1 (cyan), or shared by the two proteins (white). **(I)** Internalized Integrin-β1 in shRNA-expressing HeLa cells after uptake of α-Integrin-β1 and following a 4-h chase. *n* = 4. **(A, C, G, and I)** Data are means ± SEM; *, P < 0.05; **, P < 0.01; ***, P < 0.001; and ****, P < 0.0001. **(B, D, F, and H)** Insets show magnifications of the boxed areas. Scale bars: 10 µm; scale bars of insets: 2 µm.

**Figure S7.**
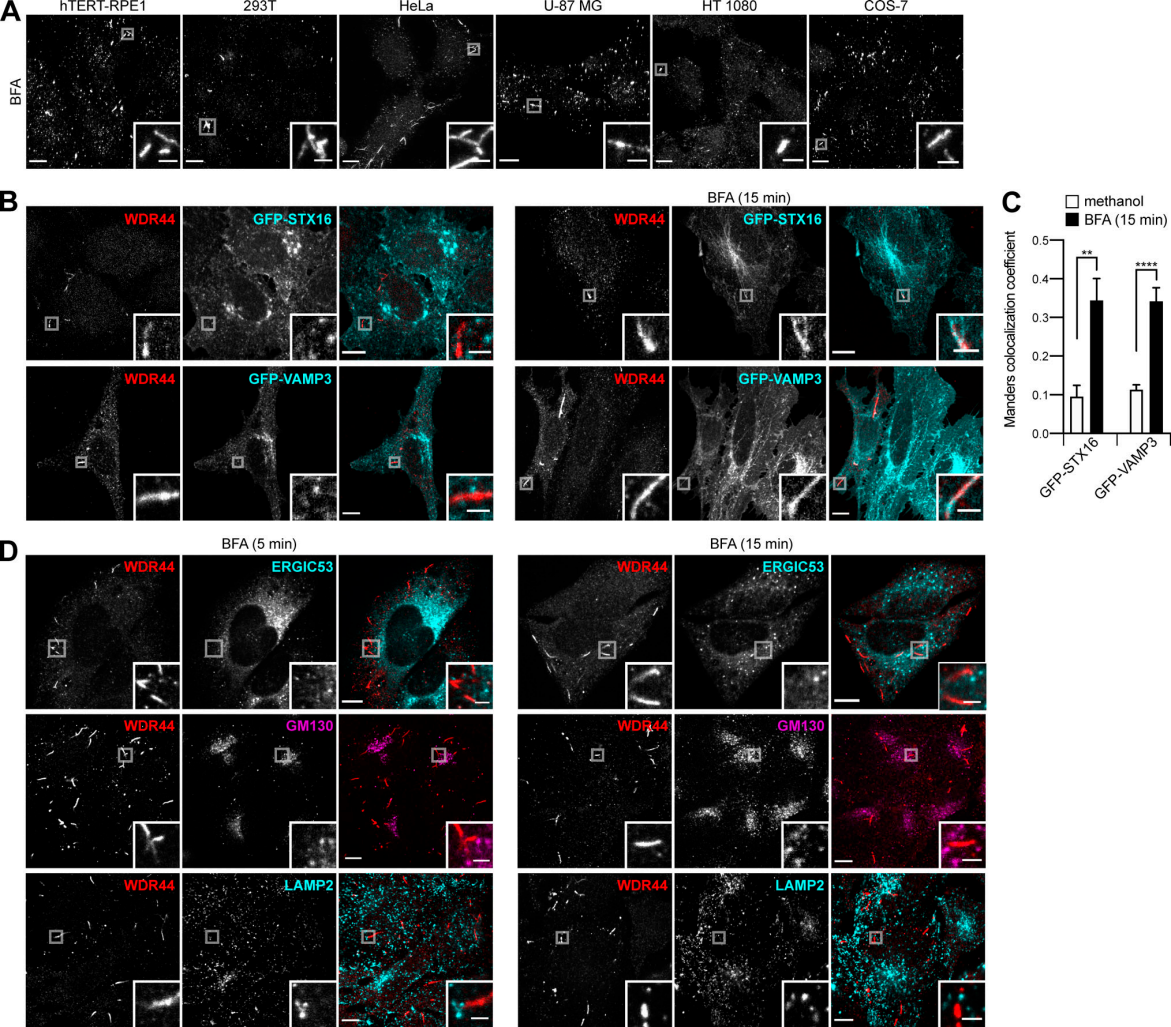
**WDR44 labels a subset of tubular endosomes. (A)** Confocal stacks of hTERT-RPE1, 293T, HeLa, U-87 MG, HT 1080, and COS-7 cells incubated with BFA (5 µg/ml, 15 min) and stained with α-WDR44. All cell types showed an increase in endogenous WDR44 tubules (compare with Fig. S3 E). **(B)** Confocal images of transfected HeLa cells left untreated or after incubation with BFA (5 µg/ml, 15 min) and stained with α-WDR44. While there was no colocalization of endogenous WDR44 tubules with GFP-STX16 or GFP-VAMP3 under resting conditions, colocalization was seen after incubation with BFA. **(C)** Manders colocalization coefficients for GFP-STX16 and GFP-VAMP3 with endogenous WDR44 structures. *n* = 10**–**20 cells. Data are means ± SEM; **, P < 0.01; and ****, P < 0.0001. **(D)** Confocal images of untransfected HeLa cells incubated with BFA (5 µg/ml) for 5 or 15 min and stained with α-WDR44 and α-ERGIC53, α-GM130, or α-LAMP2. Endogenous WDR44 tubules did not colocalize with any of these proteins. **(A, B, and D)** Insets show magnifications of the boxed areas. Scale bars: 10 µm; scale bars of insets: 2 µm.

To identify the compartments with which WDR44 tubules communicate, we used BFA, a drug that leads to the tubulation of certain organelles and the mixing of groups of intracellular compartments (Lippincott-Schwartz et al., 1991). Incubation of cells with BFA increased WDR44 tubulation (Fig. 7 A and Fig. S7 A). WDR44 was not responsible for the tubulation of the TGN (Video 10), but WDR44 tubules colocalized with TGN46 after 5 min and internalized Transferrin after 15 min (Fig. 7, B and C). Colocalization was also seen with other markers of the TGN (STX16) and recycling endosomes (VAMP3; Fig. S7, B and C). There was no increase in colocalization with ERGIC53, GM130, or LAMP2 (Fig. 7 C and Fig. S7 D). These observations suggest that WDR44 tubules are part of the TGN-endosome–plasma membrane homotypic membrane system (Lippincott-Schwartz et al., 1991). In agreement, when all proteins were overexpressed, STX16 and VAMP3 were found in WDR44 tubules, even without BFA (Fig. 7 D).

**Video 10.** **WDR44 is not responsible for the BFA-induced tubulation of the TGN.** HeLa cells were transfected with GFP-WDR44 and TGN46-RFP and imaged with a confocal spinning disk. Snapshots were captured at 5-s intervals and are shown here at seven frames per second. BFA (5 µg/ml) was added after 2 min. Scale bar: 10 µm.

**Figure S8.**
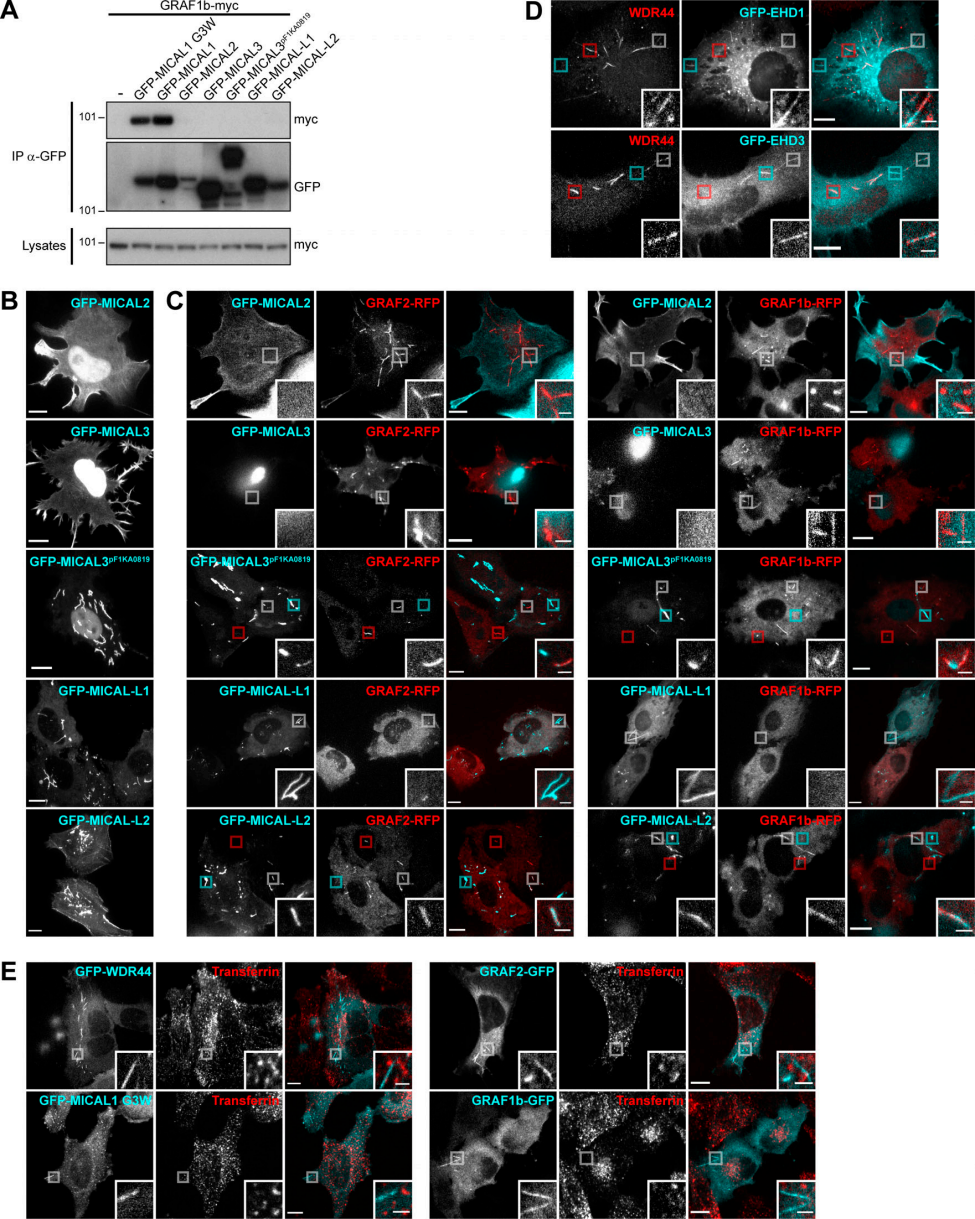
**Interplay of GRAF/WDR44 with other proteins of the MICAL family and with recycling endosomes. (A)** Immunoprecipitation (IP) of transfected 293T cells with α-GFP. GRAF1b-myc was coimmunoprecipitated by GFP-MICAL1 and GFP**–**MICAL1 G3W but not by other members of the MICAL family. **(B)** Confocal stacks of transfected HeLa cells. As reported before, GFP-MICAL2 and GFP-MICAL3 were mostly nuclear but were also found at the plasma membrane (Giridharan and Caplan, 2014). A longer isoform of MICAL3, GFP-MICAL3^pF1KA0819^, was also nuclear but labeled thick and relatively static cytoplasmic tubular structures (Grigoriev et al., 2011). GFP**–**MICAL-L1 localized to intracellular tubules and puncta. GFP**–**MICAL-L2 was found at the plasma membrane and on intracellular puncta and tubules. **(C)** Confocal images of transfected HeLa cells. GFP-MICAL2, GFP-MICAL3, and GFP**–**MICAL-L1 did not colocalize with GRAF1b/2**–**RFP. GFP-MICAL3^pF1KA0819^ and GFP**–**MICAL-L2 displayed partial colocalization with GRAF1b/2**–**RFP tubules. Red boxed areas correspond to GRAF1b/2 tubules devoid of MICAL3^pF1KA0819^/MICAL-L2; cyan boxed areas correspond to MICAL3^pF1KA0819^/MICAL-L2 structures devoid of GRAF1b/2; white boxed areas show regions of colocalization and are magnified. Upon cotransfection of GFP**–**MICAL-L1, GRAF1b/2**–**RFP were essentially cytosolic and not found on tubules. **(D)** Confocal images of transfected HeLa cells stained with α-WDR44. Boxed areas show tubules positive only for WDR44 (red), only for GFP**–**EHD1/3 (cyan), or shared by WDR44 and GFP**–**EHD1/3 (white). **(E)** Confocal images of transfected HeLa cells incubated with Alexa Fluor 546**–**Transferrin (10 µg/ml, 30 min). GFP-tagged WDR44, MICAL1 G3W, GRAF2, or GRAF1b did not colocalize with Transferrin-positive endosomes. **(B–E)** Insets show magnifications of the boxed areas. Scale bars: 10 µm; scale bars of insets: 2 µm.

These results are consistent with the involvement of Rab8, Rab10, and Rab11 in protein export and recycling. In addition to MICAL1, three other members of the MICAL family can bind Rab8 and Rab10 and have been involved in similar processes: MICAL3, MICAL-L1, and MICAL-L2 (Rai et al., 2016; Grigoriev et al., 2011; Sharma et al., 2009; Yamamura et al., 2008; Sun et al., 2016). Although none of them coimmunoprecipitated GRAF1b/2 (Fig. 7 E and Fig. S8 A), some GRAF1b/2 tubules colocalized with MICAL-L2 and MICAL3^pF1KA0819^, and overexpression of MICAL-L1 inhibited GRAF1b/2 tubulation (Fig. S8, B and C). The same was true for endogenous WDR44 (Fig. 7, F and G). Significantly however, a few WDR44 tubules colocalized with endogenous MICAL-L1 (Fig. 7 H) and with two other proteins of tubular endosomes, GFP-EHD1 and EHD3 (Fig. S8 D; Sharma et al., 2009). Unlike what has been reported for MICAL-L1, however, down-regulation of WDR44 or GRAF2 did not perturb recycling of internalized Integrin-β1 (Fig. 7 I), a clathrin-independent cargo, and GRAF1b/2, WDR44, and MICAL1 G3W did not colocalize with internalized Transferrin (Fig. S8 E), a clathrin-dependent cargo (Xie et al., 2016).

### GRAF2 and WDR44 are involved in the specific export of neosynthesized E-cadherin, MMP14, CFTR, and CFTR ΔF508

**Figure 8.**
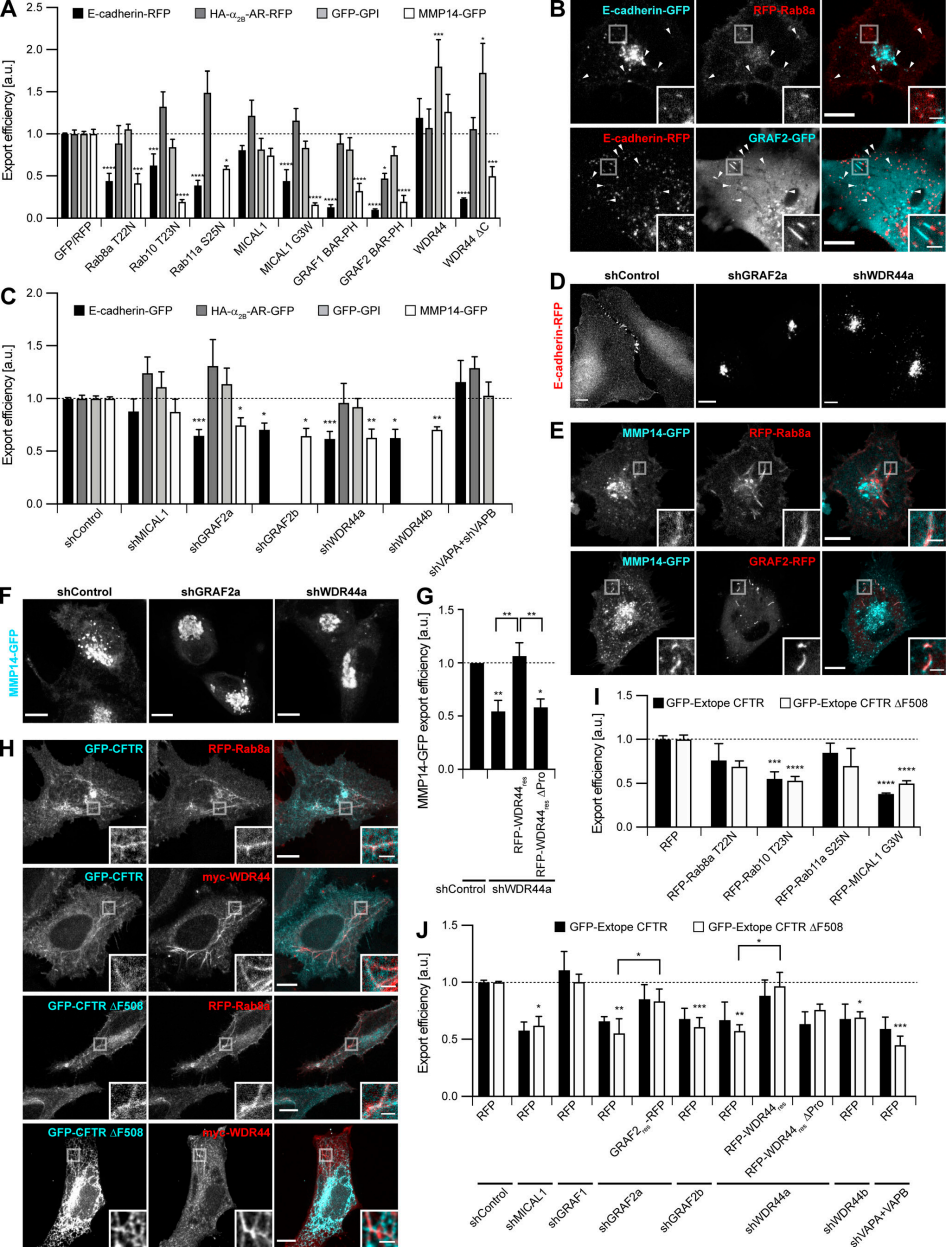
**GRAF2 and WDR44 are involved in the specific export of neosynthesized E-cadherin, MMP14, and CFTR ΔF508. (A)** Export efficiency of E-cadherin**–**RFP, HA**–**α_2B_-AR**–**RFP, and GFP-GPI in HeLa cells and of MMP14-GFP in HT 1080 cells upon cotransfection of GFP or RFP-tagged proteins, as appropriate. *n* = 3**–**8. **(B)** Confocal images of HeLa cells 8 h after transfection. E-cadherin was found in many Rab8a- and GRAF2-positive puncta (arrowheads) or was often associated with GRAF2-positive tubules. **(C)** Export efficiency of E-cadherin**–**GFP, HA**–**α_2B_-AR**–**GFP, and GFP-GPI in shRNA-expressing HeLa cells and of MMP14-GFP in shRNA-expressing HT 1080 cells. *n* = 4**–**14. **(D)** Confocal stacks of transfected HeLa cells. In shGRAF2- and shWDR44a-expressing cells, E-cadherin**–**RFP was trapped in an intracellular compartment. **(E)** Confocal images of transfected HT 1080 cells 8 h after transfection showing colocalization of MMP14-GFP with RFP-Rab8a and GRAF2-RFP in intracellular tubules. **(F)** Confocal stacks of transfected HT 1080 cells. In shGRAF2a- and shWDR44a-expressing cells, MMP14-GFP was trapped in an intracellular compartment. **(G)** Export efficiency of MMP14-GFP in shRNA-expressing HT 1080 cells without or with RFP-WDR44_res_ or RFP**–**WDR44_res_ ΔPro. *n* = 5**–**7. **(H)** Confocal images of transfected HeLa cells. GFP-CFTR and GFP**–**CFTR ΔF508 colocalized with RFP-Rab8a and myc-WDR44 tubules. myc-WDR44 was detected with α-myc staining. **(I)** Export efficiency of GFP-Extope CFTR and GFP-Extope CFTR ΔF508 in transfected 293T cells. *n* = 3**–**9. **(J)** Export efficiency of GFP**–**Extope CFTR or GFP**–**Extope CFTR ΔF508 in 293T cells cotransfected with the indicated shRNAs and RFP, GRAF2_res_-RFP, RFP-WDR44_res_, or RFP**–**WDR44_res_ ΔPro. *n* = 3**–**10. **(A, C, G, I, and J) **Data are means ± SEM; *, P < 0.05; **, P < 0.01; ***, P < 0.001; and ****, P < 0.0001. The dashed line represents the export efficiency under control conditions. **(B, D–F, and H)** Insets show magnifications of the boxed areas. Scale bars: 10 µm; scale bars of insets: 2 µm.

**Figure S9.**
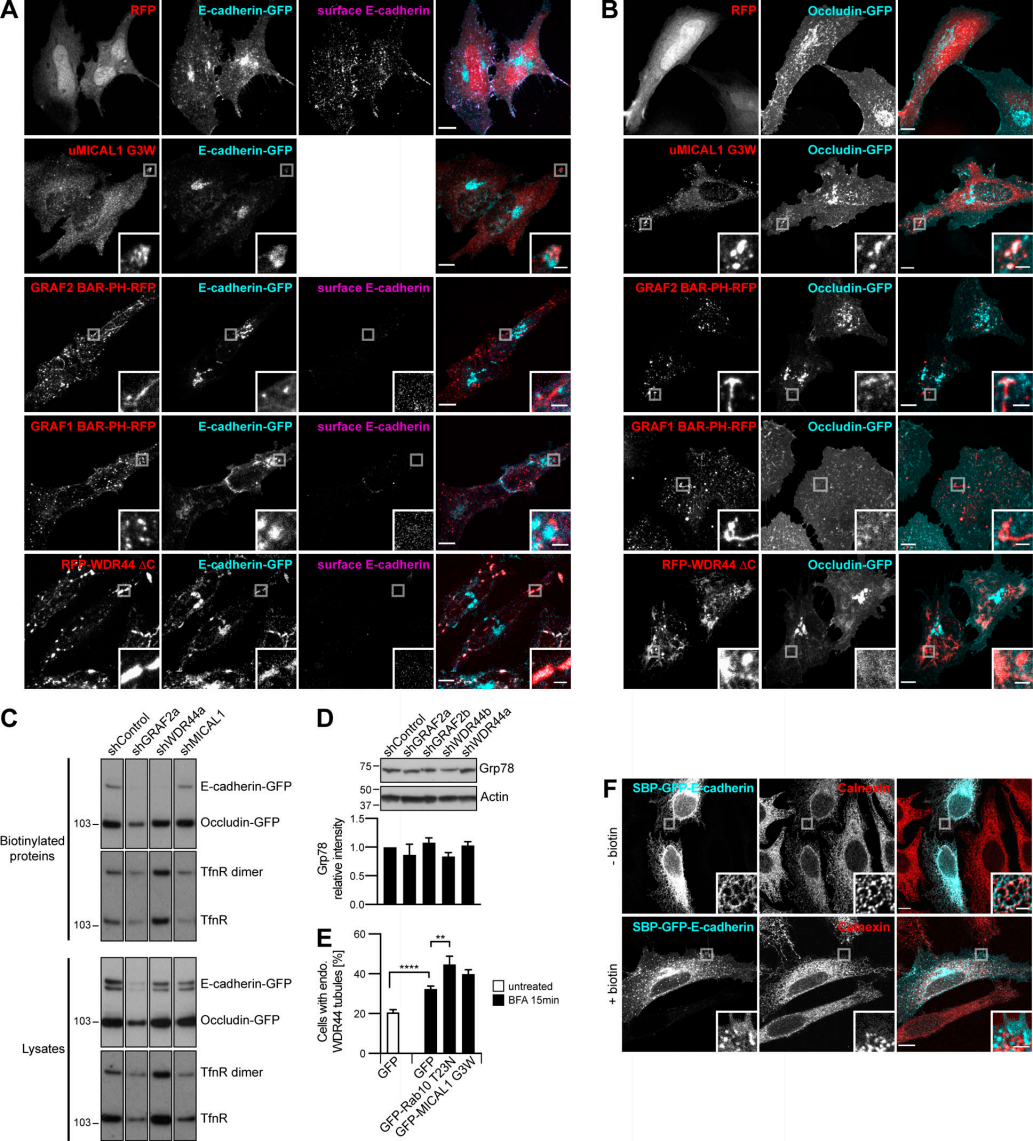
**GRAF2 and WDR44 are involved in the specific export of neosynthesized E-cadherin, MMP14, and CFTR ΔF508. (A)** Confocal stacks of transfected HeLa cells stained with α**–**E-cadherin under nonpermeabilizing conditions or with α-MICAL1 under permeabilizing conditions. Upon cotransfection of all the dominant negative constructs, E-cadherin**–**GFP became trapped in intracellular compartments. E-cadherin**–**GFP colocalized with untagged MICAL1 (uMICAL1) G3W puncta and RFP**–**WDR44 ΔC patches. **(B)** Confocal stacks of transfected HeLa cells stained, in the case of uMICAL1 G3W, with α-MICAL1. Occludin-GFP was transported to the plasma membrane in all cases. Occludin-GFP was found in uMICAL1 G3W puncta, but not in WDR44 ΔC patches. **(C)** Biotinylation of surface proteins of shRNA-expressing HeLa cells transfected with E-cadherin**–**GFP and Occludin-GFP. The Transferrin receptor (TfnR) was used as loading control; it was also found as a dimer. The four parts of the blots are from the same membranes and correspond to identical exposure times. **(D)** Western blot analysis of an equal protein amount of shRNA-expressing HeLa cell lysates. The intensity of the Grp78 protein band was normalized by the Actin signal and expressed as a ratio to the value obtained for shControl-transfected cells. *n* = 2. Down-regulation of GRAF2 or WDR44 expression did not lead to an increase in Grp78 expression, a marker of ER stress (Lee, 2005). **(E)** Percentage of transfected HeLa cells, incubated or not with BFA (5 µg/ml, 15 min), with endogenous (endo.) WDR44 tubules. *n* = 24**–**31 for GFP-transfected cells and 4**–**6 for the others. Unlike under resting conditions (Fig. 4 D and Fig. 5 F), Rab10 T23N and MICAL1 G3W did not inhibit BFA-induced WDR44 tubules, suggesting that these may not be functional for protein export. **(F)** Confocal images of transfected HeLa cells, left untreated or incubated with biotin (40 µM, 1 h) and stained with α-Calnexin. In untreated cells, SBP-GFP**–**E-cadherin was in the ER and was released to the plasma membrane upon incubation with biotin.** (D and E) **Data are means ± SEM; **, P < 0.01; and ****, P < 0.0001. **(A, B, and F)** Insets show magnifications of the boxed areas. Scale bars: 10 µm; scale bars of insets: 2 µm.

In addition to recycling, Rab8, Rab10, and Rab11 regulate the export of a subset of neosynthesized proteins. Since WDR44-positive tubules were induced by BFA (Fig. 7 A), we focused on candidates that were also reported to use an unconventional pathway of export. We first examined E-cadherin, an adhesion protein essential for epithelial morphogenesis and behaving as a tumor suppressor (Pal et al., 2018). In agreement with Lock and Stow (2005), we found that export of neosynthesized E-cadherin in HeLa cells was inhibited by Rab11a S25N (Fig. 8 A). It was also inhibited by Rab8a T22N, Rab10 T23N, MICAL1 G3W, GRAF1/2 BAR-PH, and WDR44 ΔC (Fig. 8 A and Fig. S9 A). This was specific, as export of the tight junction protein Occludin was unaffected by these mutants (Fig. S9 B). Shortly after transfection, E-cadherin was found in intracellular puncta that colocalized with Rab8 and GRAF2 and were often associated with GRAF2-positive tubules (Fig. 8 B). When GRAF2 or WDR44 was knocked down, E-cadherin was exported less efficiently (Fig. 8 C and Fig. S9 C) and was trapped in a perinuclear compartment (Fig. 8 D). Knockdown of MICAL1 led to a modest decrease (12%), albeit not statistically significant, while down-regulation of VAPA/B did not have an effect (Fig. 8 C). Export of Occludin was not affected by down-regulation of GRAF2 or WDR44 (Fig. S9 C).

We then analyzed export of the α_2B_-Adrenergic Receptor (α_2B_-AR), a G protein–coupled receptor that was reported to reach the plasma membrane in a Rab1-independent and Rab8-dependent manner (Filipeanu et al., 2006; Dong et al., 2010). We however failed to see a significant effect of Rab8a T22N on the export of α_2B_-AR, nor did we see an effect of MICAL1 G3W, GRAF1 BAR-PH, or WDR44 ΔC (Fig. 8 A). Overexpression of GRAF2 BAR-PH inhibited export by ∼50% (Fig. 8 A). However, down-regulation of GRAF2 or WDR44 expression had no effect (Fig. 8 C). By similarity with α_2B_-AR, export of the glycosylphosphatidylinositol (GPI)-anchored protein GFP-GPI was not inhibited by overexpression of Rab8a T22N, Rab10 T23N, MICAL1 G3W, GRAF1/2 BAR-PH, or WDR44 ΔC (Fig. 8 A). Neither was it inhibited by down-regulation of GRAF2 or WDR44 (Fig. 8 C).

MMP14 is a matrix metalloproteinase that targets components of the extracellular matrix and is tightly associated with tumor progression and cell migration (Egeblad and Werb, 2002). In agreement with earlier studies, we found that in HT 1080 cells grown on collagen-coated dishes, export of MMP14 was inhibited by overexpression of Rab8a T22N (Fig. 8 A; Bravo-Cordero et al., 2016, 2007; Wiesner et al., 2013). It was also inhibited by Rab10 T23N, MICAL1 G3W, GRAF1/2 BAR-PH, and WDR44 ΔC. Imaging of live cells showed that MMP14 was transported in Rab8a- and GRAF2-positive tubules (Fig. 8 E). In addition, export of MMP14 was decreased in cells in which GRAF2 or WDR44 was knocked down (Fig. 8 C), where it accumulated in perinuclear structures (Fig. 8 F). In shWDR44a-expressing cells, export of MMP14 was restored upon cotransfection of the shRNA-resistant protein RFP-WDR44_res_, but not RFP–WDR44_res_ ΔPro (Fig. 8 G), a mutant that cannot bind GRAF1b/2 (Fig. 2 C and Fig. S2 D). By similarity with E-cadherin, export was not significantly perturbed by down-regulation of MICAL1 (12% reduction; Fig. 8 C).

CFTR is a channel responsible for the transport of anions across the plasma membrane of a variety of epithelial cells. It can follow several routes of export as it progresses through the secretory pathway (Yoo et al., 2002; Egan et al., 2002; Rennolds et al., 2008; Gee et al., 2011). Knockdown of Rab8 or Rab11 was shown to inhibit its apical delivery (Vogel et al., 2015). Of particular relevance for patients with cystic fibrosis (Elborn, 2016), deletion of F508 leads to intracellular retention of the channel and degradation. Surface exposure of CFTR ΔF508 can be helped by small molecules, such as VX-809 and C4. VX-809, which aids protein folding (Farinha et al., 2015; Loo and Clarke, 2017), limits the degradation of misfolded CFTR ΔF508 and, as such, leads to an increase in the expression level not only of the fully glycosylated Golgi-modified protein but also of the immature unglycosylated protein (Farinha et al., 2013, 2015). C4, on the other hand, limits the degradation of CFTR ΔF508 by lysosomes (Farinha et al., 2013; Holleran et al., 2013). The combination of VX-809 and C4 is potent in stabilizing plasma membrane levels of CFTR ΔF508, irrespective of its pathway of export. When expressed in HeLa cells, CFTR and CFTR ΔF508 colocalized with Rab8a and WDR44 (Fig. 8 H). In 293T cells, overexpression of Rab10 T23N and MICAL1 G3W significantly decreased export of CFTR and CFTR ΔF508 (Fig. 8 I). Down-regulation of GRAF2, WDR44, MICAL1, and VAPA/B expression inhibited export of CFTR ΔF508, which, in the case of GRAF2 and WDR44, was rescued by overexpression of shRNA-resistant clones, but not by WDR44_res_ ΔPro (Fig. 8 J). Export of CFTR was also sensitive to knockdown of GRAF2, WDR44, MICAL1, and VAPA/B, but to a lesser degree, suggesting that for the wild-type protein, other pathways may compensate more efficiently. Down-regulation of GRAF1, which is also expressed in 293T cells (Fig. S1 E), did not significantly perturb export of CFTR ΔF508 (Fig. 8 J).

These experiments together show that unlike Occludin, α_2B_-AR, and GPI-anchored proteins, E-cadherin, MMP14, CFTR, and CFTR ΔF508 reach the plasma membrane in a Rab8/10-, GRAF2-, and WDR44-dependent manner.

### Export of neosynthesized CFTR, CFTR ΔF508, E-cadherin, and MMP14 is sensitive to ER stress

**Figure 9.**
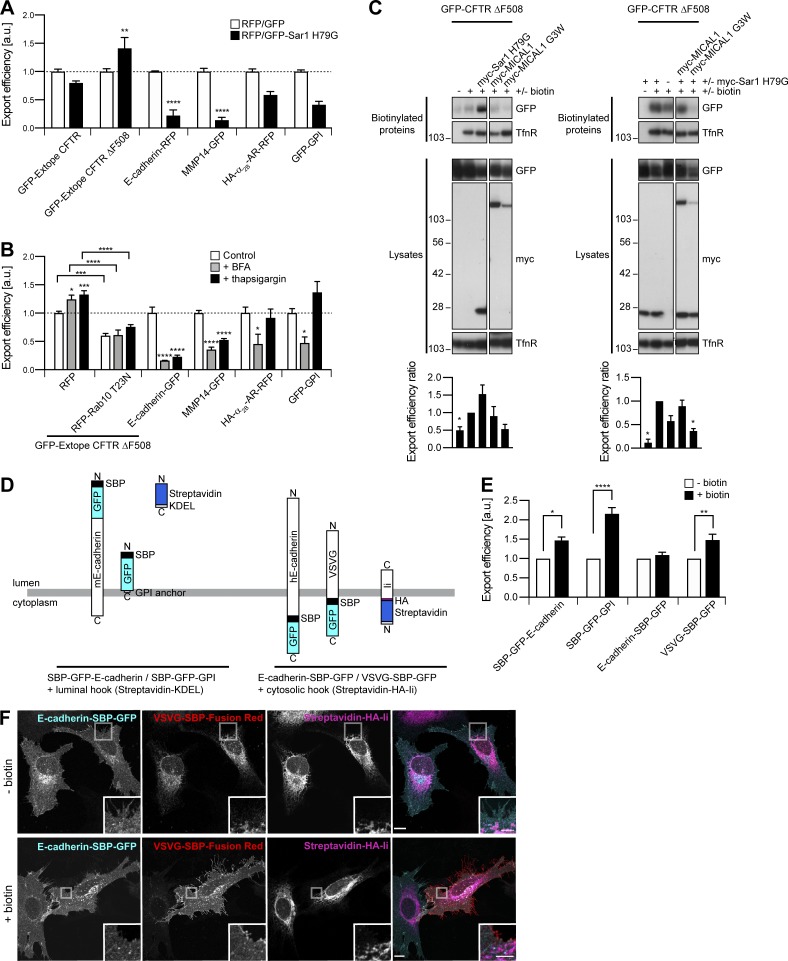
**Export of neosynthesized CFTR, CFTR ΔF508, E-cadherin, and MMP14 is sensitive to ER stress. (A)** Export efficiency of GFP**–**Extope CFTR or GFP**–**Extope CFTR ΔF508 in 293T cells, of E-cadherin**–**RFP, HA**–**α_2B_-AR**–**RFP, or GFP-GPI in HeLa cells and of MMP14-GFP in HT 1080 cells. *n* = 3**–**6. Sar1 H79G increased export of GFP**–**Extope CFTR ΔF508 but inhibited export of E-cadherin**–**RFP and MMP14-GFP. **(B)** Export efficiency of GFP**–**Extope CFTR ΔF508 in 293T cells and of E-cadherin**–**GFP, MMP14-GFP, HA**–**α_2B_-AR**–**RFP, or GFP-GPI in HeLa cells. For GFP**–**Extope CFTR ΔF508, cells were left untreated, incubated with BFA (1.25 µg/ml, 12 h), or thapsigargin (1 µM, 2 h), the latter followed by a 2-h chase (Gee et al., 2011). Cells were collected 36 h after transfection. HeLa cells were incubated with BFA (1.25 µg/ml, 6 h) or thapsigargin (1 µM, 6 h) and collected 12 h after transfection. Basal and ER stress-induced export of GFP**–**Extope CFTR ΔF508 was inhibited by RFP**–**Rab10 T23N. ER stress inhibited export of E-cadherin**–**GFP and MMP14-GFP. *n* = 4**–**6. **(A and B)** The dashed line represents the export efficiency under control conditions. **(C)** Biotinylation of surface proteins in transfected 293T cells. Export efficiency of GFP**–**CFTR ΔF508 was quantified using single-transfected cells (left) or cells cotransfected with myc**–**Sar1 H79G (right) as control. *n* = 3**–**5. The Transferrin receptor (TfnR) was used as loading control. In each case, the two parts of the blots are from the same membrane and correspond to identical exposure times. **(D)** Schematic representation of the RUSH proteins used. **(E and F)** Transfected HeLa cells were left untreated or incubated with biotin (40 µM, 1 h). **(E)** Export efficiency of RUSH proteins. *n* = 3**–**5. **(A, B, C, and E)** Data are means ± SEM; *, P < 0.05; **, P < 0.01; ***, P < 0.001; and ****, P < 0.0001. **(F)** Confocal images after staining with DyLight 650 α-HA. In untreated cells, VSVG-SBP**–**Fusion Red was in the ER where it colocalized with the Streptavidin-HA-Ii retention hook; E-cadherin**–**SBP-GFP did not accumulate in the ER and was already found at the plasma membrane. Upon biotin addition, VSVG-SBP-GFP was released from the ER and translocated to the plasma membrane. Insets show magnifications of the boxed areas. Scale bars: 10 µm; scale bars of insets: 4 µm.

In previous studies, ER stress was shown to induce export of CFTR ΔF508 through a Golgi bypass route (Gee et al., 2011). In agreement, overexpression of Sar1 H79G, a dominant negative mutant interfering with COPII coat dynamics, stimulated export of CFTR ΔF508 (Fig. 9 A). This was also seen after incubation of the cells with BFA or with thapsigargin, an ER Ca^2+^-ATPase inhibitor (Fig. 9 B). Strikingly, Sar1 H79G–induced export of CFTR ΔF508 was inhibited by MICAL1 G3W (Fig. 9 C). By similarity, BFA and thapsigargin-induced export were inhibited by Rab10 T23N (Fig. 9 B). These results suggest that basal and stress-induced exports of CFTR ΔF508 converge in Rab10-associated tubular endosomes.

Contrasting with CFTR ΔF508, we found that export of E-cadherin and MMP14 was inhibited by Sar1 H79G overexpression or BFA addition, even more so than export of GFP-GPI and α_2B_-AR (Fig. 9, A and B). Moreover, unlike GFP-GPI and α_2B_-AR, E-cadherin and MMP14 were also trapped in cells in the presence of thapsigargin (Fig. 9 B). This suggests that their export is particularly sensitive to ER stress in general, which prompted us to verify that knocking down WDR44 and GRAF2 did not induce it (Fig. S9 D; Lee, 2005). As BFA increased endogenous WDR44 tubules (Fig. 7 A), this might not be the ER stress-sensitive step of the export pathway of E-cadherin and MMP14. Alternatively, BFA-induced WDR44 tubules might not be functional for protein export, as they were still induced in the presence of Rab10 T23N or MICAL1 G3W (Fig. S9 E). To examine at which point E-cadherin diverges from the classical pathway of secretion, we expressed a fusion protein developed for the Retention Using Selective Hooks (RUSH) system (Boncompain et al., 2012). Streptavidin Binding Protein (SBP)-GFP–E-cadherin consists of the N-terminal signal sequence of interleukin-2 followed by SBP, GFP, and finally mouse E-cadherin without its prodomain (Fig. 9 D). In agreement with Boncompain et al. (2012), SBP-GFP–E-cadherin was trapped in the ER and released with biotin addition (Fig. 9 E and Fig. S9 F). This was also true for SBP-GFP-GPI (Fig. 9 E). However, when the full sequence of human E-cadherin was used to make E-cadherin–SBP-GFP (Fig. 9 D), the protein already reached the plasma membrane in untreated cells (Fig. 9 F). Biotin addition failed to induce a significant increase in plasma membrane E-cadherin–SBP-GFP, contrary to VSVG-SBP-GFP (Fig. 9 E). This suggests that E-cadherin–SBP-GFP already diverges from the classical pathway of secretion in the ER, as it escapes retention by an ER-resident hook.

## Discussion

In this study, we report the identification of three novel proteins—GRAF2, WDR44, and MICAL1—participating in the export of a subset of neosynthesized proteins. E-cadherin, MMP14, CFTR, and CFTR ΔF508 were previously found to reach the plasma membrane in a Rab8- and/or Rab11-dependent manner (Bravo-Cordero et al., 2007; Wiesner et al., 2013; Lock and Stow, 2005; Desclozeaux et al., 2008; Vogel et al., 2015). We now show that their export is also sensitive to overexpression of dominant negative Rab10, MICAL1, GRAF1b/2, and WDR44 mutants and inhibited by knockdown of GRAF2 or WDR44. This is specific, as exports of a GPI-anchored protein, of α_2B_-AR, and of Occludin are not affected. Apart from CFTR ΔF508, their export is only moderately inhibited by MICAL1 knockdown, maybe because a catalytic amount of MICAL1 is sufficient to target GRAF2 to Rab10/8–positive membranes. This would be coherent with the lower MICAL1 expression in 293T cells (in which CFTR ΔF508 export was assessed) and with the fact that we did not see endogenous MICAL1 tubules under resting conditions. Alternatively, other proteins might substitute for MICAL1, although we have shown that none of the other MICAL family members interact with GRAF1b/2. Contrasting with our original expectation, but in agreement with others (Geng et al., 2012; Proulx-Bonneau et al., 2011; Chu et al., 2004; Zucker et al., 2002), we found that BFA inhibits the export of E-cadherin and MMP14. We further show that this is true for other inducers of ER stress and is specific, as export of α_2B_-AR and of a GPI-anchored protein are unaffected by incubation of the cells with thapsigargin. As inhibition of ER to Golgi transport invariably leads to ER stress (Hikiji et al., 2015; Gee et al., 2011), we now show that it can also specifically block protein export indirectly and therefore cannot be used as proof that a protein enters the classical pathway of secretion. Our results do not allow us to determine whether E-cadherin and MMP14 bypass the Golgi or not, but the fact that E-cadherin–SBP-GFP was not trapped in the ER by an ER-resident hook suggests that E-cadherin already segregates from other cargo in the ER. In agreement, the exit of E-cadherin from the ER was proposed to require binding of the phosphatidylinositol-(4,5)-bisphosphate (PI(4,5)P_2_)–generating enzyme PIPKIγ, something that was reported to be abolished by ER stress (Geng et al., 2012). Nevertheless, as E-cadherin and MMP14 accumulate in a perinuclear compartment in cells in which GRAF2 or WDR44 has been knocked down, GRAF2/WDR44 tubules are probably not directly involved in transport out of the ER, but at a later step. This step might be shared with stress-induced unconventional secretion that bypasses the Golgi, as MICAL1 G3W and Rab10 T23N also inhibit stress-induced export of CFTR ΔF508. Our results therefore support the existence of at least three modes of protein export: (1) a classical route using COPII-coated vesicles followed by GPI-anchored proteins and α_2B_-AR, insensitive to ER stress but blocked by Sar1 H79G and BFA; (2) a route inhibited by ER stress followed by E-cadherin, MMP14, CFTR, and CFTR ΔF508 that may or may not use COPII-coated vesicles and at some stage depends on Rab8, Rab10, GRAF2, and WDR44; and (3) a stress-induced pathway followed by CFTR ΔF508 that bypasses the Golgi and is also dependent on Rab10.

**Figure 10.**
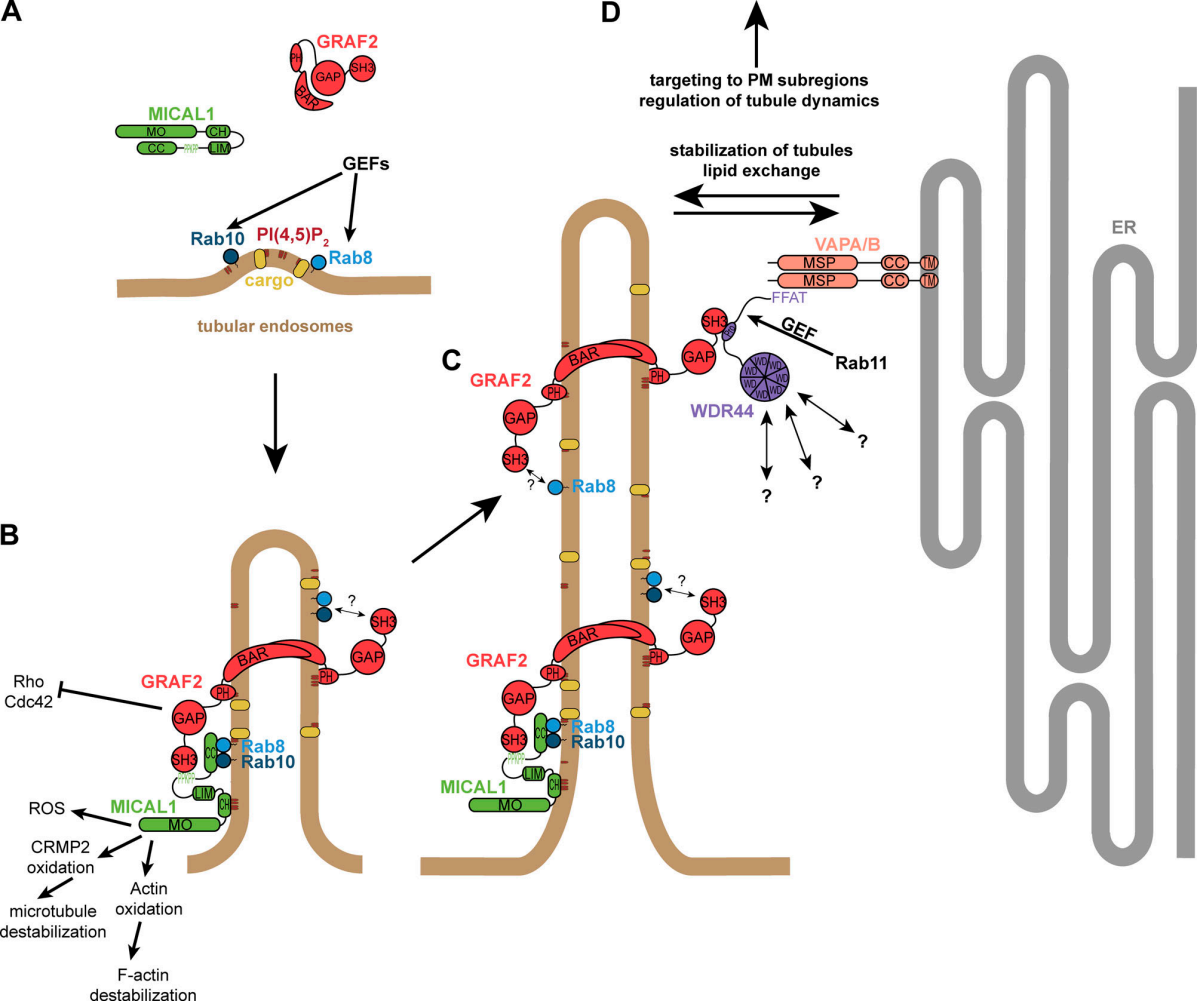
**Working model for the formation of MICAL1/GRAF2/WDR44 tubules. (A)** Activation of Rab10 and then Rab8a/b by unknown guanine nucleotide exchange factors (GEFs) promotes their association with budding tubules, which originate in the perinuclear region of cells and could be described as a subset of tubular endosomes. Cargo selection proceeds by an unknown mechanism, which may be related to a specific lipid environment such as an enrichment in PI(4,5)P_2_ or cholesterol (Ling et al., 2007; Tan et al., 2015; Annabi et al., 2001; Seveau et al., 2004), partitioning in highly curved membranes, direct binding of Rabs or adapters (Ling et al., 2007; Lock and Stow, 2005; Lau and Mruk, 2003), and/or cytoskeletal retention (MacDonald et al., 2018). **(B)** GTP-bound Rab10 and Rab8a/b recruit and activate MICAL1, which may bind two Rabs simultaneously (Rai et al., 2016; Esposito et al., 2018). MICAL1 may also bind directly to the membrane via its PI(4,5)P_2_-binding CH domain (Alqassim et al., 2016). MICAL1 can now recruit and activate GRAF2, which by similarity to GRAF1b is expected to directly bind to PI(4,5)P_2–_containing membranes via its PH domain (Lundmark et al., 2008). The BAR domain of GRAF2 increases its affinity for Rab8/10 membranes and promotes tubule extension. Activated GRAF1b/2 and MICAL1 trigger rearrangements of the cytoskeleton, which may contribute to regulating the stability and dynamics of the tubules. Other unknown proteins (?) may also bridge GRAF2 and Rab8/10. **(C)** The MO activity of MICAL1 leads to its dissociation from intracellular membranes and allows GRAF2 to recruit WDR44. The association of Rab8 with WDR44-positive tubules is more stable than that of Rab10. **(D)** WDR44 bridges GRAF2-positive tubules to the ER proteins VAPA/B and, via its WD repeats that fold in a β-propeller, may recruit additional unknown proteins (?) involved in trafficking. See text for further details. GAP, GTPase Activating Protein; ROS, reactive oxygen species; CC, coiled coil; MSP, Major Sperm Protein; TM, transmembrane; PM, plasma membrane.

In recent years, proteins of the GRAF family have attracted attention because they are mutated in cancer (Li et al., 2017; Aissani et al., 2015; Bozóky et al., 2013; Qian et al., 2010) and have been associated with neuropsychiatric diseases (Barresi et al., 2010; Jarius and Wildemann, 2015; Dahm et al., 2014), but little mechanistic information has been published. We show here that direct binding of proline-rich regions of MICAL1 and WDR44 to the SH3 domain of GRAF1/2 allows GRAF-mediated trafficking to be controlled by Rab8, Rab10, and Rab11 (see model in Fig. 10). Rab8, Rab10, MICAL1, GRAF1b/2, and WDR44 are dynamically associated with the same intracellular tubules. Activation of Rab8a is essential for GRAF1b/2 to be recruited. Our results suggest that GRAF1b/2 can be brought to preformed Rab8/10 tubules via MICAL1 binding and that both MICAL1 and GRAF1b/2 contribute to Rab8/10 tubule extension. The MO activity of MICAL1 regulates the turnover of its complex with GRAF1b/2, as overexpression of an enzymatically inactive MICAL1 G3W mutant prevents recruitment of WDR44. Knockdown of GRAF2, Rab8, or Rab10 inhibits formation of endogenous WDR44 tubules, while overexpression of GRAF1b/2 or of constitutively active mutants of Rab8 and Rab10 induces them. With seven WD repeats that fold into β-propellers (Li and Roberts, 2001), WDR44 is likely to constitute an interaction platform for the recruitment of yet-to-be-identified proteins that could contribute to tubule elongation, membrane scission, transport, or fusion. Our results also indicate that although Rab11a is not a stable component of GRAF1b/2 tubules, it indirectly regulates the pathway via WDR44. MICAL1/GRAF/WDR44 constitutes a new molecular path that can connect Rab11 to Rab8/10, as suggested before for Rabin-8 (Knödler et al., 2010) and Myosin Vb (Roland et al., 2011).

Overexpression of Rab8 is known to induce a decrease in F-actin and focal adhesions in a RhoA-dependent manner (Hattula et al., 2006; Bravo-Cordero et al., 2016). We now show that GRAF1b/2 and MICAL1 could be involved, GRAF1b/2 via their GTPase Activating Protein (GAP) domain (Ren et al., 2001; Taylor et al., 1999; Doherty et al., 2011a) and MICAL1 via its MO domain (Giridharan and Caplan, 2014). Under resting conditions, both MICAL1 and GRAF1b/2 are in an auto-inhibited state (Eberth et al., 2009; Giridharan et al., 2012; Schmidt et al., 2008). This could be released in response to Rab8 and MICAL1 binding, respectively, and allow the three proteins to cooperate in triggering rearrangements of the cytoskeleton (Nüchel et al., 2018; Frémont et al., 2017). Reciprocally, we also report that similar to Rab8 tubules, endogenous WDR44 tubules are stabilized on microtubules and are induced by destabilization of F-actin. In the case of Rab8, Cytochalasin D was proposed to accelerate macropinocytosis, leading to an increase in trafficking through recycling endosomes (Hattula et al., 2006). This may also be responsible for the increase in WDR44 tubules, as similar to Rab8 and Rab10 (Henry and Sheff, 2008; Ang et al., 2003; Gerges et al., 2004; Etoh and Fukuda, 2019; Shibata et al., 2016), we have shown that WDR44 tubules are part of the TGN-endosome–plasma membrane homotypic membrane system (Lippincott-Schwartz et al., 1991). GRAF2-dependent WDR44 tubules could thus also be described as tubular endosomes, which correspond to a heterogeneous ensemble of compartments, at the crossroad between endocytic and exocytic pathways (Cresawn et al., 2007; Baetz and Goldenring, 2013; Powelka et al., 2004; Xie et al., 2014). We have shown partial colocalization of endogenous WDR44 with another protein associated with tubular endosomes, MICAL-L1. Recently, overexpression of GRAF1c was reported to decrease the number of endogenous MICAL-L1 tubules (Cai et al., 2014). We made the reciprocal observation: overexpression of MICAL-L1 inhibits WDR44 and GRAF1b/2 tubulation. This suggests competition rather than cooperation between GRAF/WDR44– and MICAL-L1–mediated trafficking. In agreement, unlike GRAF1b/2 and WDR44, the subcellular distribution of MICAL-L1 is insensitive to knockdown of Rab8 and Rab10 (Kobayashi et al., 2014; Rahajeng et al., 2012); and unlike silencing of MICAL-L1 (Sharma et al., 2009), silencing of GRAF2 and WDR44 does not interfere with Integrin-β1 recycling. As knockdown of GRAF1 in 293T cells does not significantly interfere with the export of CFTR ΔF508, it is possible that GRAF1c participates in MICAL-L1–mediated protein recycling, but that together with WDR44, GRAF2 is specifically involved in exocytosis of neosynthesized proteins.

Our results also reveal a new feature of GRAF2/WDR44 tubules: they are closely aligned with ER membranes via binding of WDR44 to VAPA/B. Even though VAPA/B were proposed to play a role in protein export and in membrane trafficking (Peretti et al., 2008; Dong et al., 2016; Wakana et al., 2015; Kuijpers et al., 2013; Saita et al., 2009), it is the first time that contact sites between a membrane trafficking intermediate and the ER have been observed. We have shown that down-regulation of VAPA/B expression inhibits endogenous WDR44 tubules. However, among the cargos tested, this only affected export of CFTR ΔF508, indicating that VAPA/B are not mechanistically essential for GRAF2/WDR44–derived trafficking and that the effect on CFTR ΔF508 could also be related to other changes resulting from VAPA/B knockdown (Dong et al., 2016; Wakana et al., 2015). VAP-mediated ER contacts might therefore simply contribute to the formation and/or stabilization of membrane tubules on a preexisting reticular scaffold, control their dynamics (Dong et al., 2016; Raiborg et al., 2015; Rocha et al., 2009), or help target the tubules, for example, to PI(4,5)P_2_–enriched microdomains of the plasma membrane (Pan et al., 2018). But they might also allow lipid exchange with the ER (Peretti et al., 2008; Perry and Ridgway, 2006; Mesmin et al., 2013; Kim et al., 2015), indirectly promoting cargo selection. Of interest, Rab10 was proposed to play a role in the growth of new ER tubules by regulating the formation of a domain enriched in lipid-synthesizing enzymes (English and Voeltz, 2013). Therefore, if GRAF2/WDR44 were also associated with these domains, they could play a role in transporting newly synthesized lipids directly out of the ER and indirectly regulate the morphology and dynamics of the ER.

Our results have deepened our understanding of the mechanism of GRAF2 and Rab8/Rab10–mediated trafficking. Of interest, although little was known about the function of mammalian WDR44 and MICAL1, they were both proposed to play a role in protein export (Mammoto et al., 2000; Van Battum et al., 2014; Grauzam et al., 2018). Given the importance of the proper regulation of plasma membrane levels of E-cadherin and MMP14 in cancer and of CFTR in cystic fibrosis, it is essential to identify the molecular pathways that control their trafficking. Our results now show that in addition to Rab8/10/11, GRAF2, WDR44, and MICAL1 are also important.

## Materials and methods

### Cloning

In general, cDNAs were amplified from cDNA libraries, IMAGE clones, or plasmids and were integrated in the entry vector pDONR201 for cloning using the Gateway recombination system (Thermo Fisher Scientific). For expression in mammalian cells, the entry clones were recombined with modified pCI (Promega) vectors, engineered to be Gateway compatible and to express proteins with a GFP (EGFP protein), RFP (tagRFP protein), or myc tag in N-terminal or C-terminal position. The empty destination (DEST) vectors pCI N-GFP DEST and pCI N-RFP DEST were used for expression of soluble EGFP and tagRFP, respectively. For expression of GST-tagged proteins in bacteria, entry clones were recombined with a modified pGex 4T2 vector (GE Healthcare Life Sciences) engineered to be Gateway compatible. Mutagenesis was performed by PCR amplification of parent clones, digestion of the template with DpnI, ligation, and transformation. Apart from Rab8a and Rab1, which were canine, EHD1 which was from mouse, and Sar1 which was from rat, all the other constructs were human. pCI GRAF2-GFP and pCI GRAF2-RFP were recombined from pDONR GRAF2 C-tag (made from a PCR of IMAGE clone 40027832 using 5′-GGG​GAC​AAG​TTT​GTA​CAA​AAA​AGC​AGG​CTT​CGA​AGG​AGA​TAG​AAC​CAT​GGG​GCT​GCA​GCC​CCT​GGA​G-3′ and 5′-GGG​GAC​CAC​TTT​GTA​CAA​GAA​AGC​TGG​GTC​CAG​CAG​CTT​GAC​GTA​GTT​CTG​TGG-3′ with a silent point mutation introduced to abolish unwanted recombinations; Lucken-Ardjomande Häsler et al., 2014); pCI untagged GRAF2 (uGRAF2) was recombined from pDONR GRAF2 (made from a PCR of pDONR GRAF2 C-tag using 5′-GGG​GAC​AAG​TTT​GTA​CAA​AAA​AGC​AGG​CTT​CGA​AGG​AGA​TAG​AAC​CAT​GGG​GCT​GCA​GCC​CCT​GGA​G-3′ and 5′-GGG​GAC​CAC​TTT​GTA​CAA​GAA​AGC​TGG​GTC​CCT​ACA​GCA​GCT​TGA​CGT​AGT​TCT​G-3′); pGex 4T2 GRAF2 SH3 was recombined from pDONR GRAF2 SH3 N-tag (aa 718–786 of GRAF2, made from a PCR of pDONR GRAF2 C-tag using 5′-GGG​GAC​AAG​TTT​GTA​CAA​AAA​AGC​AGG​CTT​CGC​TAC​TGT​AGC​GGA​CAA​GCC​ACC-3′ and 5′-GGG​GAC​CAC​TTT​GTA​CAA​GAA​AGC​TGG​GTC​CCT​ACA​GCA​GCT​TGA​CGT​AGT​TCT​G-3′); pCI GRAF2 BAR-PH–GFP and pCI GRAF2 BAR-PH–RFP were recombined from pDONR GRAF2 BAR-PH C-tag (aa 1–387 of GRAF2, made from a PCR of pDONR GRAF2 C-tag using 5′-GGG​GAC​AAG​TTT​GTA​CAA​AAA​AGC​AGG​CTT​CGA​AGG​AGA​TAG​AAC​CAT​GGG​GCT​GCA​GCC​CCT​GGA​G-3′ and 5′-GGG​GAC​CAC​TTT​GTA​CAA​GAA​AGC​TGG​GTC​ATT​TCC​TTC​TGG​TCT​TGG​GAT​GAT​GGC-3′); pCI GRAF2_res_-GFP and pCI GRAF2_res_-RFP were recombined from pDONR GRAF2_res_ C-tag (mutagenesis of pDONR GRAF2 C-tag with 5′-CCC​ATT​TGA​GCA​TCG​ATC​TGG​CGG​GAA​ACT​TGG-3′ and 5′-CCA​AGT​TTC​CCG​CCA​GAT​CGA​TGC​TCA​AAT​GGG-3′); pCI GRAF2_res_ ΔBAR-GFP was recombined from pDONR GRAF2_res_ ΔBAR C-tag (aa 241–786 of GRAF2, made from a PCR of pDONR GRAF2_res_ C-tag using 5′-GGG​GAC​AAG​TTT​GTA​CAA​AAA​AGC​AGG​CTT​CGA​AGG​AGA​TAG​AAC​CAT​GGG​AAC​AAG​GTC​AGA​AGT​GGA​AGA​GC-3′ and 5′-GGG​GAC​CAC​TTT​GTA​CAA​GAA​AGC​TGG​GTC​CAG​CAG​CTT​GAC​GTA​GTT​CTG​TGG-3′); pCI GRAF1b-GFP, pCI GRAF1b-RFP, and pCI GRAF1b-myc were recombined from pDONR GRAF1b C-tag (made from a PCR of a human brain cDNA library using 5′-GGG​GAC​AAG​TTT​GTA​CAA​AAA​AGC​AGG​CTT​CGA​AGG​AGA​TAG​AAC​CAT​GGG​GCT​CCC​AGC​GCT​CGA​G-3′ and 5′-GGG​GAC​CAC​TTT​GTA​CAA​GAA​AGC​TGG​GTC​GAG​GAA​CTC​CAC​GTA​ATT​CTC​AGG​G-3′); pGex 4T2 GRAF1 SH3 was recombined from pDONR GRAF1 SH3 N-tag (aa 669–759 of GRAF1b, made from a PCR of pDONR GRAF1b C-tag using 5′-GGG​GAC​AAG​TTT​GTA​CAA​AAA​AGC​AGG​CTT​CCC​AAC​TTC​ACC​CCT​CTC​GCC​ATC-3′ and 5′-GGG​GAC​CAC​TTT​GTA​CAA​GAA​AGC​TGG​GTC​CCT​AGA​GGA​ACT​CCA​CGT​AAT​TCT​CAG​GG-3′); pCI GRAF1 BAR-PH–GFP and pCI GRAF1 BAR-PH–RFP were recombined from pDONR GRAF1 BAR-PH C-tag (aa 1–381 of GRAF1b, made from a PCR of pDONR GRAF1b C-tag using 5′-GGG​GAC​AAG​TTT​GTA​CAA​AAA​AGC​AGG​CTT​CGA​AGG​AGA​TAG​AAC​CAT​GGG​GCT​CCC​AGC​GCT​CGA​G-3′ and 5′-GGG​GAC​CAC​TTT​GTA​CAA​GAA​AGC​TGG​GTC​AGT​CCC​TTC​ACT​CTG​GCT​GTC​TTT​G-3′); pCI myc-WDR44, pCI GFP-WDR44, and pCI RFP-WDR44 were recombined from pDONR WDR44 N-tag (made from a PCR of IMAGE 4837674 using 5′-GGG​GAC​AAG​TTT​GTA​CAA​AAA​AGC​AGG​CTT​CGC​GTC​GGA​AAG​CGA​CAC​C-3′ and 5′-GGG​GAC​CAC​TTT​GTA​CAA​GAA​AGC​TGG​GTC​CCT​AAG​ATA​CAT​TTT​TTC​TTT​TAT​TAA​CAA​ACA​CTT​TG-3′); pCI WDR44-GFP and pCI WDR44-RFP were recombined from pDONR WDR44 C-tag (made from a PCR of IMAGE 4837674 using 5′-GGG​GAC​AAG​TTT​GTA​CAA​AAA​AGC​AGG​CTT​CGA​AGG​AGA​TAG​AAC​CAT​GGC​GTC​GGA​AAG​CGA​CAC​C-3′ and 5′-GGG​GAC​CAC​TTT​GTA​CAA​GAA​AGC​TGG​GTC​AGA​TAC​ATT​TTT​TCT​TTT​ATT​AAC​AAA​CAC​TTT​G-3′); pCI GFP-WDR44 ΔFFAT was recombined from pDONR WDR44 ΔFFAT N-tag (aa 16–913 of WDR44, made from a PCR of pDONR WDR44 N-tag using 5′-GGG​GAC​AAG​TTT​GTA​CAA​AAA​AGC​AGG​CTT​CGA​TGT​GCA​CCT​AGG​GGG​CG-3′ and 5′-GGG​GAC​CAC​TTT​GTA​CAA​GAA​AGC​TGG​GTC​CCT​AAG​ATA​CAT​TTT​TTC​TTT​TAT​TAA​CAA​ACA​CTT​TG-3′); pCI GFP-WDR44 ΔN1 was recombined from pDONR WDR44 ΔN1 N-tag (aa 201–913 of WDR44, made from a PCR of pDONR WDR44 N-tag using 5′-GGG​GAC​AAG​TTT​GTA​CAA​AAA​AGC​AGG​CTT​CAA​AGA​TTT​TGC​CGC​TGT​GGA​AG-3′ and 5′-GGG​GAC​CAC​TTT​GTA​CAA​GAA​AGC​TGG​GTC​CCT​AAG​ATA​CAT​TTT​TTC​TTT​TAT​TAA​CAA​ACA​CTT​TG-3′); pCI GFP-WDR44 ΔN2 and pGex 4T2 WDR44 ΔN2 were recombined from pDONR WDR44 ΔN2 N-tag (aa 473–913 of WDR44, made from a PCR of pDONR WDR44 N-tag using 5′-GGG​GAC​AAG​TTT​GTA​CAA​AAA​AGC​AGG​CTT​CGA​TGA​TGA​AGG​AAT​GCC​ATA​CAC-3′ and 5′-GGG​GAC​CAC​TTT​GTA​CAA​GAA​AGC​TGG​GTC​CCT​AAG​ATA​CAT​TTT​TTC​TTT​TAT​TAA​CAA​ACA​CTT​TG-3′); pCI GFP-WDR44 ΔC, pCI myc-WDR44 ΔC, pCI RFP-WDR44 ΔC, and pGex 4T2 WDR44 ΔC were recombined from pDONR WDR44 ΔC N-tag (aa 1–472 of WDR44, made from a PCR of pDONR WDR44 N-tag using 5′-GGG​GAC​AAG​TTT​GTA​CAA​AAA​AGC​AGG​CTT​CGC​GTC​GGA​AAG​CGA​CAC​C-3′ and 5′-GGG​GAC​CAC​TTT​GTA​CAA​GAA​AGC​TGG​GTC​CCT​AAC​TTG​ATG​AAG​GAT​CAT​CTT​GAT​CAG-3′); pCI GFP-WDR44 ΔPro was recombined from pDONR WDR44 ΔPro N-tag (deletion of aa 210–257 of WDR44 by mutagenesis of pDONR WDR44 N-tag using 5′-GGC​CAC​TTC​TTC​CAC​AGC​GG-3′ and 5′-AGA​AAA​AGG​AAA​AGC​GAA​TTG​GAA​TTT​G-3′); pCI RFP-WDR44_res_ was recombined from pDONR WDR44_res_ N-tag (mutagenesis of pDONR WDR44 N-tag using 5′-GTG​GCT​TTG​TGG​AAC​GAG​GTA​GAC​GGT​CAG​ACA​AAA​TTG​ATC-3′ and 5′-GAT​CAA​TTT​TGT​CTG​ACC​GTC​TAC​CTC​GTT​CCA​CAA​AGC​CAC-3′); pCI RFP-WDR44_res_ ΔPro was recombined from pDONR WDR44_res_ ΔPro N-tag (mutagenesis of pDONR WDR44 ΔPro N-tag using 5′-GTG​GCT​TTG​TGG​AAC​GAG​GTA​GAC​GGT​CAG​ACA​AAA​TTG​ATC-3′ and 5′-GAT​CAA​TTT​TGT​CTG​ACC​GTC​TAC​CTC​GTT​CCA​CAA​AGC​CAC-3′); pCI myc-MICAL1, pCI GFP-MICAL1, pCI RFP-MICAL1, and pGex 4T2 MICAL1 were recombined from pDONR MICAL1 N-tag (made from a PCR of IMAGE 5756097 using 5′-GGG​GAC​AAG​TTT​GTA​CAA​AAA​AGC​AGG​CTT​CGC​TTC​ACC​TAC​CTC​CAC​CAA​CC-3′ and 5′-GGG​GAC​CAC​TTT​GTA​CAA​GAA​AGC​TGG​GTC​CCT​AGC​CCT​GGG​CCC​CTG​TCC-3′); pCI MICAL1-GFP was recombined from pDONR MICAL1 C-tag (made from a PCR of IMAGE 5756097 using 5′-GGG​GAC​AAG​TTT​GTA​CAA​AAA​AGC​AGG​CTT​CGA​AGG​AGA​TAG​AAC​CAT​GGC​TTC​ACC​TAC​CTC​CAC​CAA​CC-3′ and 5′-GGG​GAC​CAC​TTT​GTA​CAA​GAA​AGC​TGG​GTC​GCC​CTG​GGC​CCC​TGT​CC-3′); pCI untagged MICAL1 (uMICAL1) was recombined from pDONR MICAL1 (made from a PCR of pDONR MICAL1 N-tag using 5′-GGG​GAC​AAG​TTT​GTA​CAA​AAA​AGC​AGG​CTT​CGA​AGG​AGA​TAG​AAC​CAT​GGC​TTC​ACC​TAC​CTC​CAC​CAA​CC-3′ and 5′-GGG​GAC​CAC​TTT​GTA​CAA​GAA​AGC​TGG​GTC​CCT​AGC​CCT​GGG​CCC​CTG​TCC-3′); pGex 4T2 MICAL1 ΔN was recombined from pDONR MICAL1 ΔN N-tag (aa 191–1067 of MICAL1, made from a PCR of pDONR MICAL1 N-tag using 5′-GGG​GAC​AAG​TTT​GTA​CAA​AAA​AGC​AGG​CTT​CGG​GAG​TGG​CTG​GCG​TGC-3′ and 5′-GGG​GAC​CAC​TTT​GTA​CAA​GAA​AGC​TGG​GTC​CCT​AGC​CCT​GGG​CCC​CTG​TCC-3′); pCI GFP-MICAL1 ΔMO was recombined from pDONR MICAL1 ΔMO N-tag (aa 503–1067 of MICAL1, made from a PCR of pDONR MICAL1 N-tag using 5′-GGG​ACA​AGT​TTG​TAC​AAA​AAA​GCA​GGC​TTC​CCA​GCC​ACC​GGG​TCG​GC-3′ and 5′-GGG​GAC​CAC​TTT​GTA​CAA​GAA​AGC​TGG​GTC​CCT​AGC​CCT​GGG​CCC​CTG​TCC-3′); pCI GFP-MICAL1 ΔMOΔCH and pGex 4T2 MICAL1 ΔMOΔCH were recombined from pDONR MICAL1 ΔMOΔCH N-tag (aa 651–1067 of MICAL1, made from a PCR of pDONR MICAL1 N-tag using 5′-GGG​GAC​AAG​TTT​GTA​CAA​AAA​AGC​AGG​CTT​CGC​AGA​GGA​TGC​TGG​TGG​C-3′ and 5′-GGG​GAC​CAC​TTT​GTA​CAA​GAA​AGC​TGG​GTC​CCT​AGC​CCT​GGG​CCC​CTG​TCC-3′); pCI myc-MICAL1^tail^ was recombined from pDONR MICAL1^tail^ N-tag (aa 901–1067 of MICAL1, made from a PCR of pDONR MICAL1 N-tag using 5′-GGG​GAC​AAG​TTT​GTA​CAA​AAA​AGC​AGG​CTT​CGG​CAC​CAT​GAA​TAA​CTA​CCC​AAC-3′ and 5′-GGG​GAC​CAC​TTT​GTA​CAA​GAA​AGC​TGG​GTC​CCT​AGC​CCT​GGG​CCC​CTG​TCC-3′); pCI myc-MICAL1^Pro^ was recombined from pDONR MICAL1^Pro^ N-tag (aa 800–850 of MICAL1, made from a PCR of pDONR MICAL1 N-tag using 5′-GGG​GAC​AAG​TTT​GTA​CAA​AAA​AGC​AGG​CTT​CCA​GCC​CAC​CCG​TCG​GC-3′ and 5′-GGG​GAC​CAC​TTT​GTA​CAA​GAA​AGC​TGG​GTC​CCT​ACA​CAA​AGC​TGC​TCT​CCA​GGG-3′); pCI GFP-MICAL1 G3W, pCI RFP-MICAL1 G3W, and pCI myc-MICAL1 G3W were recombined from pDONR MICAL1 G3W N-tag (MICAL1 G91W G93W G96W by mutagenesis of pDONR MICAL1 N-tag using 5′-CAA​GTG​CCT​GGT​GGT​GTG​GGC​TTG​GCC​TTG​CTG​GCT​GCG​GGT​CGC​TGT​GG-3′ and 5′-CCA​CAG​CGA​CCC​GCA​GCC​AGC​AAG​GCC​AAG​CCC​ACA​CCA​CCA​GGC​ACT​TG-3′); pCI uMICAL1 G3W was recombined from pDONR MICAL1 G3W (mutagenesis of pDONR MICAL1 using 5′-CAA​GTG​CCT​GGT​GGT​GTG​GGC​TTG​GCC​TTG​CTG​GCT​GCG​GGT​CGC​TGT​GG-3′ and 5′-CCA​CAG​CGA​CCC​GCA​GCC​AGC​AAG​GCC​AAG​CCC​ACA​CCA​CCA​GGC​ACT​TG-3′); pCI myc-MICAL1 PPAPP, pCI GFP-MICAL1 PPAPP, and pCI RFP-MICAL1 PPAPP were recombined from pDONR MICAL1 PPAPP N-tag (MICAL1 K832A by mutagenesis of pDONR MICAL1 N-tag using 5′-GGA​GCC​TCC​ACC​CGC​GCC​TCC​CCG​CAG​C-3′ and 5′-GCT​GCG​GGG​AGG​CGC​GGG​TGG​AGG​CTC​C-3′); pCI RFP-Rab8a, pCI GFP-Rab8a, and pCI myc-Rab8a were recombined from pDONR Rab8a N-tag (made from a PCR of pEGFP cRab8 using 5′-GGG​GAC​AAG​TTT​GTA​CAA​AAA​AGC​AGG​CTT​CGC​GAA​GAC​CTA​CGA​TTA​CCT​G-3′ and 5′-GGG​GAC​CAC​TTT​GTA​CAA​GAA​AGC​TGG​GTC​CCT​ACA​GAA​GAA​CAC​ATC​GGA​AAA​AGC-3′); pCI RFP-Rab8a T22N and pCI GFP-Rab8a T22N were recombined from pDONR Rab8a T22N N-tag (mutagenesis of pDONR Rab8a N-tag using 5′-GGG​GGT​GGG​GAA​GAA​CTG​TGT​CCT​GTT​CC-3′ and 5′-GGA​ACA​GGA​CAC​AGT​TCT​TCC​CCA​CCC​CC-3′); pCI RFP-Rab8a Q67L and pCI GFP-Rab8a Q67L were recombined from pDONR Rab8a Q67L N-tag (made from a PCR of pEGFP cRab8 Q67L using 5′-GGG​GAC​AAG​TTT​GTA​CAA​AAA​AGC​AGG​CTT​CGC​GAA​GAC​CTA​CGA​TTA​CCT​G-3′ and 5′-GGG​GAC​CAC​TTT​GTA​CAA​GAA​AGC​TGG​GTC​CCT​ACA​GAA​GAA​CAC​ATC​GGA​AAA​AGC-3′); pCI RFP-Rab8b and pCI GFP-Rab8b were recombined from pDONR Rab8b N-tag (made from a PCR of IMAGE 4701429 using 5′-GGG​GAC​AAG​TTT​GTA​CAA​AAA​AGC​AGG​CTT​CGC​GAA​GAC​GTA​CGA​TTA​TCT​C-3′ and 5′-GGG​GAC​CAC​TTT​GTA​CAA​GAA​AGC​TGG​GTC​CCT​AAA​GTA​GCG​AGC​AAC​GAA​AG-3′); pCI RFP-Rab10 and pCI GFP-Rab10 were recombined from pDONR Rab10 N-tag (made from a PCR of a 293T cDNA library using 5′-GGG​GAC​AAG​TTT​GTA​CAA​AAA​AGC​AGG​CTT​CGC​GAA​GAA​GAC​GTA​CGA​CCT​GC-3′ and 5′-GGG​GAC​CAC​TTT​GTA​CAA​GAA​AGC​TGG​GTC​CCT​AGC​AGC​ATT​TGC​TCT​TCC​AGC​C-3′); pCI GFP-Rab10 T23N and pCI RFP-Rab10 T23N were recombined from pDONR Rab10 T23N N-tag (mutagenesis of pDONR Rab10 N-tag using 5′-GGA​GTG​GGG​AAG​AAC​TGC​GTC​CTT​TTT​C-3′ and 5′-GGA​ATC​CCC​GAT​CAG​GAG​CAG​C-3′); pCI GFP-Rab10 Q68L was recombined from pDONR Rab10 Q68L N-tag (mutagenesis of pDONR Rab10 N-tag using 5′-GGG​ATA​CAG​CAG​GCC​TGG​AGC​GAT​TTC​AC-3′ and 5′-ATA​TCT​GTA​GCT​TGA​TCT​TCT​TTC​C-3′); pCI GFP-Rab11a and pCI RFP-Rab11a were recombined from pDONR Rab11a N-tag (PCR of IMAGE 3510339 using 5′-GGG​GAC​AAG​TTT​GTA​CAA​AAA​AGC​AGG​CTT​CGG​CAC​CCG​CGA​CGA​CGA​G-3′ and 5′-GGG​GAC​CAC​TTT​GTA​CAA​GAA​AGC​TGG​GTC​CCT​AGA​TGT​TCT​GAC​AGC​ACT​GCA​CC-3′); pCI GFP-Rab11a S25N, and pCI RFP-Rab11a S25N were recombined from pDONR Rab11a S25N N-tag (mutagenesis of pDONR Rab11a N-tag using 5′-GAG​ATT​CTG​GTG​TTG​GAA​AGA​ATA​ATC​TCC​TGT​CTC​G-3′ and 5′-CGA​GAC​AGG​AGA​TTA​TTC​TTT​CCA​ACA​CCA​GAA​TCT​C-3′); pCI GFP-Rab11a Q70L was recombined from pDONR Rab11a Q70L N-tag (mutagenesis of pDONR Rab11a N-tag using 5′-GAT​ATG​GGA​CAC​AGC​AGG​GCT​AGA​GCG​ATA​TCG​AGC-3′ and 5′-GCT​CGA​TAT​CGC​TCT​AGC​CCT​GCT​GTG​TCC​CAT​ATC-3′); pCI GFP-Rab1 S25N was recombined from pDONR Rab1 S25N N-tag (made from a PCR of pEGFP-C cRab1 S25N [gift from E. Boucrot (University College London, London, UK)] using 5′-GGG​GAC​AAG​TTT​GTA​CAA​AAA​AGC​AGG​CTT​CTC​CAG​CAT​GAA​TCC​CGA​ATA​TG-3′ and 5′-GGG​GAC​CAC​TTT​GTA​CAA​GAA​AGC​TGG​GTC​CCT​AGC​AGC​AAC​CTC​CAC​CTG​AC-3′); pCI GFP-Rab1 and pCI RFP-Rab1 were recombined from pDONR Rab1 N-tag (mutagenesis of pDONR Rab1 S25N N-tag using 5′-GCG​ACT​CTG​GGG​TTG​GAA​AGA​GTT​GCC​TCC​TTC​TTA​GG-3′ and 5′-CCT​AAG​AAG​GAG​GCA​ACT​CTT​TCC​AAC​CCC​AGA​GTC​GC-3′); pCI GFP-Rab1 Q70L was recombined from pDONR Rab1 Q70L N-tag (mutagenesis of pDONR Rab1 N-tag using 5′-CAA​ATA​TGG​GAC​ACA​GCA​GGC​CTA​GAA​AGA​TTT​CG-3′ and 5′-CGA​AAT​CTT​TCT​AGG​CCT​GCT​GTG​TCC​CAT​ATT​TG-3′); pCI myc-VAPA, pCI RFP-VAPA, and pCI GFP-VAPA were recombined from pDONR VAPA N-tag (made from a PCR of IMAGE 2822547 using 5′-GGG​GAC​AAG​TTT​GTA​CAA​AAA​AGC​AGG​CTT​CGC​GTC​CGC​CTC​AGG​GG-3′ and 5′-GGG​GAC​CAC​TTT​GTA​CAA​GAA​AGC​TGG​GTC​CCT​ACA​AGA​TGA​ATT​TCC​CTA​GAA​AGA​ATC​C-3′); pCI GFP-VAPA ΔCC was recombined from pDONR VAPA ΔCC N-tag (deletion of aa 151–225 of VAPA by mutagenesis of pDONR VAPA N-tag using 5′-CAG​TGG​AAC​AGC​TTT​GCT​AGG-3′ and 5′-CTT​CCT​TCA​CTT​CTT​GTT​GTA​ATT​GC-3′); pCI GFP-VAPA ΔMSP was recombined from pDONR VAPA ΔMSP N-tag (aa 152–249 of VAPA, made from a PCR of pDONR VAPA N-tag using 5′-GGG​GAC​AAG​TTT​GTA​CAA​AAA​AGC​AGG​CTT​CAA​TGC​ATC​TAA​GCA​AGA​TGG​ACC-3′ and 5′-GGG​GAC​CAC​TTT​GTA​CAA​GAA​AGC​TGG​GTC​CCT​ACA​AGA​TGA​ATT​TCC​CTA​GAA​AGA​ATC​C-3′); pCI GFP-VAPA ΔTM was recombined from pDONR VAPA ΔTM N-tag (aa 1–226 of VAPA, made from a PCR of pDONR VAPA N-tag using 5′-GGG​GAC​AAG​TTT​GTA​CAA​AAA​AGC​AGG​CTT​CGC​GTC​CGC​CTC​AGG​GG-3′ and 5′-GGG​GAC​CAC​TTT​GTA​CAA​GAA​AGC​TGG​GTC​CCT​AGG​ACT​GGT​GAC​ATT​ATC​TCT​GAA​GG-3′); pCI GFP-VAPA DD was recombined from pDONR VAPA DD N-tag (VAPA K94D M96D by mutagenesis of pDONR VAPA N-tag using 5′-GAG​TAA​ACA​CGA​CTT​TGA​CGT​ACA​GAC​AAT​TTT​TGC​TCC​ACC​A-3′ and 5′-TGG​TGG​AGC​AAA​AAT​TGT​CTG​TAC​GTC​AAA​GTC​GTG​TTT​ACT​C-3′); pCI GFP-VAPB and pCI myc-VAPB were recombined from pDONR VAPB N-tag (made from a PCR of IMAGE 3543354 using 5′-GGG​GAC​AAG​TTT​GTA​CAA​AAA​AGC​AGG​CTT​CGC​GAA​GGT​GGA​GCA​GG-3′ and 5′-GGG​GAC​CAC​TTT​GTA​CAA​GAA​AGC​TGG​GTC​CCT​ACA​AGG​CAA​TCT​TCC​CAA​TAA​TTA​C-3′); pCI GFP-MICAL2 was recombined from pDONR MICAL2 N-tag (made from a PCR of IMAGE 5275364 using 5′-GGG​GAC​AAG​TTT​GTA​CAA​AAA​AGC​AGG​CTT​CGG​GGA​AAA​CGA​GGA​TGA​GAA​G-3′ and 5′-GGG​GAC​CAC​TTT​GTA​CAA​GAA​AGC​TGG​GTC​CCT​AGC​CAA​GAA​GTG​GGT​GTA​GC-3′); pCI GFP-MICAL3 was recombined from pDONR MICAL3 N-tag (made from a PCR of IMAGE 100000333 using 5′-GGG​GAC​AAG​TTT​GTA​CAA​AAA​AGC​AGG​CTT​CGA​GGA​GAG​GAA​GCA​TGA​GAC​C-3′ and 5′-GGG​GAC​CAC​TTT​GTA​CAA​GAA​AGC​TGG​GTC​CCT​AGG​GCC​GTG​TGG​CGG​G-3′); pCI GFP-MICAL-L1 was recombined from pDONR MICAL-L1 N-tag (made from a PCR of IMAGE 100015878 using 5′-GGG​GAC​AAG​TTT​GTA​CAA​AAA​AGC​AGG​CTT​CGC​TGG​GCC​GCG​GGG-3′ and 5′-GGG​GAC​CAC​TTT​GTA​CAA​GAA​AGC​TGG​GTC​CCT​AGC​TCT​TGT​CTC​TGG​GGG​AC-3′); pCI GFP–MICAL-L2 was recombined from pDONR MICAL-L2 N-tag (made from a PCR of IMAGE 5521653 using 5′-GGG​GAC​AAG​TTT​GTA​CAA​AAA​AGC​AGG​CTT​CGC​GGC​CAT​CAG​GGC​G-3′ and 5′-GGG​GAC​CAC​TTT​GTA​CAA​GAA​AGC​TGG​GTC​CCT​ACT​GGG​AGG​GGC​TGC​TTT​TGC-3′); pCI E-cadherin–GFP and pCI E-cadherin–RFP were recombined from pDONR E-cadherin C-tag (made from a PCR of a human kidney cDNA library using 5′-GGG​GAC​AAG​TTT​GTA​CAA​AAA​AGC​AGG​CTT​CGA​AGG​AGA​TAG​AAC​CAT​GGG​CCC​TTG​GAG​CCG​C-3′ and 5′-GGG​GAC​CAC​TTT​GTA​CAA​GAA​AGC​TGG​GTC​GTC​GTC​CTC​GCC​GCC​TCC​G-3′); pCI Occludin-GFP was recombined from pDONR Occludin C-tag (made from a PCR of a human kidney cDNA library using 5′-GGG​GAC​AAG​TTT​GTA​CAA​AAA​AGC​AGG​CTT​CGA​AGG​AGA​TAG​AAC​CAT​GTC​ATC​CAG​GCC​TCT​TGA​AAG-3′ and 5′-GGG​GAC​CAC​TTT​GTA​CAA​GAA​AGC​TGG​GTC​TGT​TTT​CTG​TCT​ATC​ATA​GTC​TCC​AAC​C-3′); pCI MMP14-GFP was recombined from pDONR MMP14 C-tag (made from a PCR of a human kidney cDNA library using 5′-GGG​GAC​AAG​TTT​GTA​CAA​AAA​AGC​AGG​CTT​CGA​AGG​AGA​TAG​AAC​CAT​GTC​TCC​CGC​CCC​AAG-3′ and 5′-GGG​GAC​CAC​TTT​GTA​CAA​GAA​AGC​TGG​GTC​GAC​CTT​GTC​CAG​CAG​GG-3′); HA–α_2B_-AR–GFP and HA–α_2B_-AR–RFP were recombined from pDONR HA–α_2B_-AR C-tag (made from a PCR on IMAGE 9020527 using 5′-GGG​GAC​AAG​TTT​GTA​CAA​AAA​AGC​AGG​CTT​CGA​AGG​AGA​TAG​AAC​CAT​GTA​CCC​ATA​CGA​TGT​TCC​AGA​TTA​CGC​TGA​CCA​CCA​GGA​CCC​CTA​C-3′ and 5′-GGG​GAC​CAC​TTT​GTA​CAA​GAA​AGC​TGG​GTC​CCA​GGC​CGT​CTG​GGT​CCA​C-3′); pDONR Sar1 N-tag was made from a PCR on IMAGE 712455 using 5′-GGG​GAC​AAG​TTT​GTA​CAA​AAA​AGC​AGG​CTT​CTC​TTT​CAT​CTT​TGA​GTG​GAT​CTA​C-3′ and 5′-GGG​GAC​CAC​TTT​GTA​CAA​GAA​AGC​TGG​GTC​CCT​AGT​CAA​TAT​ACT​GGG​AAA​GCC​AGC-3′); pCI myc-Sar1 H79G, pCI GFP-Sar1 H79G, and pCI RFP-Sar1 H79G were recombined from pDONR Sar1 H79G N-tag (mutagenesis of pDONR Sar1 N-tag using 5′-CTT​TTG​ATC​TTG​GTG​GGG​GCG​AGC​AAG​CAC​GTC-3′ and 5′-GAC​GTG​CTT​GCT​CGC​CCC​CAC​CAA​GAT​CAA​AAG-3′); pCI Calnexin-GFP was recombined from pDONR Calnexin C-tag (made from a PCR on Calnexin-CFP using 5′-GGG​GAC​AAG​TTT​GTA​CAA​AAA​AGC​AGG​CTT​CGA​AGG​AGA​TAG​AAC​CAT​GGA​AGG​GAA​GTG​GTT​GCT​GTG-3′ and 5′-GGG​GAC​CAC​TTT​GTA​CAA​GAA​AGC​TGG​GTC​CTC​TCT​TCG​TGG​CTT​TCT​GTT​TC-3′); pCI GFP-STX16 was recombined from pDONR STX16 N-tag (made from a PCR of a 293T cDNA library using 5′-GGG​GAC​AAG​TTT​GTA​CAA​AAA​AGC​AGG​CTT​CGC​CAC​CAG​GCG​TTT​AAC​CG-3′ and 5′-GGG​GAC​CAC​TTT​GTA​CAA​GAA​AGC​TGG​GTC​CCT​ATC​GAG​ACT​TCA​CGC​CAA​CG-3′); pCI GFP-VAMP3 and pCI RFP-VAMP3 were recombined from pDONR VAMP3 N-tag (made from a PCR on IMAGE 3544610 using 5′-GGG​GAC​AAG​TTT​GTA​CAA​AAA​AGC​AGG​CTT​CTC​TAC​AGG​TCC​AAC​TGC​TGC-3′ and 5′-GGG​GAC​CAC​TTT​GTA​CAA​GAA​AGC​TGG​GTC​CCT​ATG​AAG​AGA​CAA​CCC​ACA​CG-3′); pCI TGN46-RFP was recombined from pDONR TGN46 C-tag (made from a PCR on IMAGE 4272702 using 5′-GGG​GAC​AAG​TTT​GTA​CAA​AAA​AGC​AGG​CTT​CGA​AGG​AGA​TAG​AAC​CAT​GCG​GTT​CGT​AGT​TGC​C-3′ and 5′-GGG​GAC​CAC​TTT​GTA​CAA​GAA​AGC​TGG​GTC​GGA​CTT​CTG​GTC​CAA​ACG​TTG​GTA-3′); pCI GFP-EHD1 was recombined from pDONR EHD1 N-tag (made from a PCR on pEGFP C3 mEHD1 using 5′-GGG​GAC​AAG​TTT​GTA​CAA​AAA​AGC​AGG​CTT​CTT​CAG​CTG​GGT​GAG​CAA​GG-3′ and 5′-GGG​GAC​CAC​TTT​GTA​CAA​GAA​AGC​TGG​GTC​CCT​ACT​CGT​GCC​TCC​GTT​TGG​AG-3′); and pCI GFP-EHD3 was recombined from pDONR EHD3 N-tag (made from a PCR on SKB-LNB hEHD3 using 5′-GGG​GAC​AAG​TTT​GTA​CAA​AAA​AGC​AGG​CTT​CTT​CAG​CTG​GCT​GGG​TAC​GG-3′ and 5′-GGG​GAC​CAC​TTT​GTA​CAA​GAA​AGC​TGG​GTC​CCT​ACT​CGG​CAA​CTT​TCC​TCT​TGG​ACG-3′).

For cloning of GFP–Extope-CFTR and GFP–Extope CFTR ΔF508, Extope CFTR and Extope CFTR ΔF508 were amplified from the corresponding plasmids (gifts from Martina Gentzsch [Marsico Lung Institute, Chapel Hill, NC]) using the primers 5′-GTA​CAA​GTC​CGG​ACT​CAG​ATC​TCG​AGC​TCA​GAG​GTC​GCC​TCT​GGA​AAA​GGC​C-3′ and 5′-CCG​GTG​GAT​CCC​GGG​CCC​GCG​GTA​CCC​TAA​AGC​CTT​GTA​TCT​TGC​ACC​TCT​TCT​TCT​GTC​TCC​TCT​TT-3′ and inserted into pEGFP-C1 (Takara). The plasmids Str-KDEL_SBP-EGFP-Ecadherin, Str-KDEL_SBP-EGFP-GPI, and Str-Ii_VSVG-SBP-EGFP were gifts from Franck Perez (Institut Curie, Paris, France). VSVG was excised with XmaI and EcoRI and replaced with human E-cadherin using primers 5′-CTT​GCC​ACA​ACC​CGG​GAG​GCG​CGC​CAT​GGG​CCC​TTG​GAG​CCG​C-3′ and 5′-CTC​GTC​CAT​GGA​ATT​CAG​GTC​GTC​CTC​GCC​GCC​TCC-3′ and the In-Fusion system (Takara Bio) to make Str-Ii_E-cadherin–SBP-EGFP. Str-Ii_VSVG-SBP–Fusion Red was made after excision of EGFP from Str-Ii_VSVG-SBP-EGFP using SbfI and XbaI and insertion of Fusion Red using primers 5′-TCA​ACG​TGA​ACC​ACC​TGC​AGG​TAT​GGT​GAG​CGA​GCT​GAT​TAA​GG-3′ and 5′-TGA​TCA​GTT​ATC​TAG​AGT​TTC​ATT​TAC​CTC​CAT​CAC​CAG​CGC-3′ and the In-Fusion system.

GFP-GPI (GPI anchoring region of hCD58 fused to GFP) was a gift from Ben Nichols (Laboratory of Molecular Biology, Cambridge, UK); GFP-MICAL3FL (GFP-MICAL3^pF1KA0819^) was a gift from Anna Akhmanova (Utrecht University, Utrecht, Netherlands); HA-GLUT4-GFP was a gift from Sam Cushman (National Institutes of Health, Bethesda, MD); pEGFP-wtCFTR, and pEGFP-ΔF508-CFTR were gifts from Ineke Braakman (Utrecht University, Utrecht, Netherlands).

For shRNA cloning, optimal shRNA sequences were determined using the siDESIGN Center tool (Dharmacon). Corresponding oligonucleotides were annealed, phosphorylated, and ligated into pLKO.1-puro (Sigma), which had been predigested with BglII and HindIII. For each gene, at least three different shRNAs were cloned and tested. The best ones were used: shControl (5′-ccg​gCG​TAC​GCG​GAA​TAC​TTC​GAt​tca​aga​gaT​CGA​AGT​ATT​CCG​CGT​ACG​ttt​ttg-3′ and 5′-aat​tca​aaa​aCG​TAC​GCG​GAA​TAC​TTC​GAt​ctc​ttg​aaT​CGA​AGT​ATT​CCG​CGT​ACG-3′); shGRAF2a (5′-ccg​gGC​ACA​GAT​CTG​GAG​GGA​AAt​tca​aga​gaT​TTC​CCT​CCA​GAT​CTG​TGC​ttt​ttg-3′ and 5′-aat​tca​aaa​aGC​ACA​GAT​CTG​GAG​GGA​AAt​ctc​ttg​aaT​TTC​CCT​CCA​GAT​CTG​TGC-3′); shGRAF2b (5′-ccg​gCG​TTG​AAA​CAC​GAG​GTA​TAt​tca​aga​gaT​ATA​CCT​CGT​GTT​TCA​ACG​ttt​ttg-3′ and 5′-aat​tca​aaa​aCG​TTG​AAA​CAC​GAG​GTA​TAt​ctc​ttg​aaT​ATA​CCT​CGT​GTT​TCA​ACG-3′); shGRAF1 (5′-ccg​gAG​GAA​GTC​CAA​GAG​AGA​AAt​tca​aga​gaT​TTC​TCT​CTT​GGA​CTT​CCt​ttt​ttg-3′ and 5′-aat​tca​aaa​aaG​GAA​GTC​CAA​GAG​AGA​AAt​ctc​ttg​aaT​TTC​TCT​CTT​GGA​CTT​CCT-3′); shWDR44a (5′-ccg​gGG​AAT​GAA​GTA​GAT​GGT​CAt​tca​aga​gaT​GAC​CAT​CTA​CTT​CAT​TCC​ttt​ttg-3′ and 5′-aat​tca​aaa​aGG​AAT​GAA​GTA​GAT​GGT​CAt​ctc​ttg​aaT​GAC​CAT​CTA​CTT​CAT​TCC-3′); shWDR44b (5′-ccg​gTG​ATG​AAA​CCT​GTG​AGA​AAt​tca​aga​gaT​TTC​TCA​CAG​GTT​TCA​TCA​ttt​ttg-3′ and 5′-aat​tca​aaa​aTG​ATG​AAA​CCT​GTG​AGA​AAT​CTC​TTG​AAT​TTC​TCA​CAG​GTT​TCA​TCA-3′); shMICAL1 (5′-ccg​gGC​ATT​GAT​CTG​GAG​AAC​ATt​tca​aga​gaA​TGT​TCT​CCA​GAT​CAA​TGC​ttt​ttg-3′ and 5′-aat​tca​aaa​aGC​ATT​GAT​CTG​GAG​AAC​ATt​ctc​ttg​aaA​TGT​TCT​CCA​GAT​CAA​TGC-3′); shRab8a1 (5′-ccg​gGA​ACA​AGT​GTG​ATG​TGA​ATt​tca​aga​gaA​TTC​ACA​TCA​CAC​TTG​TTC​ttt​ttg-3′ and 5′-aat​tca​aaa​aGA​ACA​AGT​GTG​ATG​TGA​ATt​ctc​ttg​aaA​TTC​ACA​TCA​CAC​TTG​TTC-3′) cotransfected with shRab8a2 (5′-ccg​gGA​GAA​TTA​AAC​TGC​AGA​TAt​tca​aga​gaT​ATC​TGC​AGT​TTA​ATT​CTC​ttt​ttg-3′ and 5′-aat​tca​aaa​aGA​GAA​TTA​AAC​TGC​AGA​TAt​ctc​ttg​aaT​ATC​TGC​AGT​TTA​ATT​CTC-3′); shRab10 (5′-ccg​gGA​ATA​GAC​TTC​AAG​ATC​AAt​tca​aga​gaT​TGA​TCT​TGA​AGT​CTA​TTC​ttt​ttg-3′ and 5′-aat​tca​aaa​aGA​ATA​GAC​TTC​AAG​ATC​AAt​ctc​ttg​aaT​TGA​TCT​TGA​AGT​CTA​TTC-3′); shRab11a (5′-ccg​gAG​TTT​AAT​CTG​GAA​AGC​AAt​tca​aga​gaT​TGC​TTT​CCA​GAT​TAA​ACT​ttt​ttg-3′ and 5′-aat​tca​aaa​aAG​TTT​AAT​CTG​GAA​AGC​AAt​ctc​ttg​aaT​TGC​TTT​CCA​GAT​TAA​ACT-3′); shVAPA (5′-ccg​gCC​ACA​GAC​CTC​AAA​TTC​AAt​tca​aga​gaT​TGA​ATT​TGA​GGT​CTG​TGG​ttt​ttg-3′ and 5′-aat​tca​aaa​aCC​ACA​GAC​CTC​AAA​TTC​AAt​ctc​ttg​aaT​TGA​ATT​TGA​GGT​CTG​TGG-3′); and shVAPB (5′-ccg​gCG​GAA​GAC​CTT​ATG​GAT​TCt​tca​aga​gag​aAT​CCA​TAA​GGT​CTT​CCG​ttt​ttg-3′ and 5′-aat​tca​aaa​aCG​GAA​GAC​CTT​ATG​GAT​TCt​ctc​ttg​aaG​AAT​CCA​TAA​GGT​CTT​CCG-3′).

For lentivirus production, pMD2.G (Addgene plasmid 12259; RRID:Addgene_12259) and PSPAX2 (Addgene plasmid 12260; RRID:Addgene_12260) were used. Both were gifts from Didier Trono (École Polytechnique Fédérale de Lausanne, Lausanne, Switzerland).

All constructs were verified by sequencing.

### Antibodies and reagents

Purified rabbit polyclonal α-GRAF1 and α-GRAF2 antibodies were described previously (Lucken-Ardjomande Häsler et al., 2014), as well as α-GRAF1 (Ra83; Lundmark et al., 2008). The following commercial primary antibodies were used: GFP (mouse, clones 7.1 and 13.1, Cat# 11814460001, RRID:AB_390913; Roche); GFP (rabbit, Cat# ab290, RRID:AB_303395; Abcam); c-myc (mouse, clone 9E10, Cat# M4439, RRID:AB_439694; Sigma); myc (rabbit, Cat# 2272, RRID:AB_331667; Cell Signaling Technology); HA (mouse, clone 16B12, Cat# MMS-101P, RRID:AB_291259; Covance); WDR44 (rabbit, Cat# A301-440A, RRID:AB_961125; Bethyl Laboratories); MICAL1 (mouse, clone 4B1, Cat# TA501893, RRID:AB_11140106; OriGene); Rab8 (mouse, clone 4/Rab8, Cat# 610845, RRID:AB_398164; BD Biosciences); Rab10 (mouse, clone 4E2, Cat# ab104859, RRID:AB_10711207; Abcam); Rab11 (mouse, clone 47/Rab11, Cat# 610656, RRID:AB_397983; BD Biosciences); VAPA (rabbit, Cat# NBP1_31237, RRID:AB_2213101; Novus Biologicals); VAPB (rabbit, Cat# HPA013144, RRID:AB_1858717; Sigma); Calnexin (rabbit, Cat# ab22595, RRID:AB_2069006; Abcam); Calreticulin (rabbit, Cat# 208910, RRID:AB_564320; Merck Millipore); KDEL (rat, clone MAC256, Cat# ab50601, RRID:AB_880636; Abcam); GM130 (mouse, clone 35/GM130, Cat# 610822, RRID:AB_398141; BD Biosciences); TGN46 (sheep, Cat# AHP500, RRID:AB_324049; Bio-Rad); LAMP2 (mouse, Cat# H4B4, RRID:AB_528129; deposited to the Developmental Studies Hybridoma Bank by J.T. August and J.E.K. Hildreth); ERGIC53 (mouse, clone G1/93, Cat# 804–602, RRID:AB_2234610; Alexis Biochemicals); MICAL-L1 (mouse, Cat# H00085377-B01P, RRID:AB_2143767; Novus Biologicals); Grp78 (mouse, clone 40/BiP, Cat# 610978, RRID:AB_398291; BD Biosciences); E-cadherin (rat, clone ECCD-2, Cat# 13–1900, RRID:AB_2533005; Thermo Fisher Scientific); MMP14 (rabbit, Cat# ab3644, RRID:AB_303973; Abcam); VSVG (mouse, clone 8G5F11, Cat# EB0010, RRID:AB_2811223; Kerafast); β-tubulin (mouse, clone 2–28-33, Cat# T5293, RRID:AB_477580; Sigma); Actin (mouse, clone AC-15, Cat# ab6276, RRID:AB_2223210; Abcam); Integrin-β1 (mouse, clone TS2/16, Cat# LS-C106376, RRID:AB_10626118; LifeSpan Biosciences); Alexa Fluor 647 Integrin-β1 (mouse, clone TS2/16, Cat# 303018, RRID:AB_2130080; BioLegend); Alexa Fluor 647 GFP (rabbit, Cat# A-31852, RRID:AB_162553; Thermo Fisher Scientific); and DyLight 650 conjugated HA (mouse, clone 16B12, Cat# ab117515, RRID:AB_10999718; Abcam).

The secondary reagents for Western blotting were goat α−mouse and α−rabbit IgG-HRP conjugates (Bio-Rad). HRP-Protein A (Thermo Fisher Scientific) was used for analysis of beads when antibodies used for the immunoprecipitation and for Western blotting were raised in the same species.

The secondary antibodies used for immunofluorescence were Alexa Fluor 488, 514, 546, or 647 conjugates of goat α−mouse, α−rabbit, α−rat, and α−sheep IgG antibodies (all from Thermo Fisher Scientific).

The following reagents were used: Nocodazole, Cytochalasin D, BFA, D-biotin, methanol, and DMSO (Sigma); Alexa Fluor 546–Transferrin, Alexa Fluor 647–Transferrin, Alexa Fluor 546–dextran 10.000 MW fixable, and Alexa Fluor 546–phalloidin (Thermo Fisher Scientific); Puromycin and C4 (Corr-4a; Merck Millipore); VX-809 (Lumafactor; Sellekchem); and thapsigargin (Santa Cruz Biotechnology).

### Cell lines and transfection

HeLa (ECACC 93021013), 293T (gift from Ingo Greger, Laboratory of Molecular Biology, Cambridge, UK), HT 1080 (gift from Roger Williams, Laboratory of Molecular Biology, Cambridge, UK), U-87 MG (ECACC 89081402), COS-7 (ECACC 87021302), undifferentiated 3T3 L1 cells (ECACC 86052701), and BSC1 (ECACC 85011422) cells were grown in DMEM-GlutaMAX supplemented with 10% FBS. NIH 3T3 cells (ECACC 93061524) were grown in DMEM-GlutaMAX with 10% newborn calf serum. SH-SY5Y cells (ECACC 94030304) were grown in a 1:1 mixture of MEM and Ham’s F-12 supplemented with GlutaMAX, 1% nonessential amino acids, and 15% FBS. hTERT-RPE1 cells (gift from Jonathon Pines, Institute of Cancer Research, London, UK) were grown in a 1:1 mixture of DMEM and Ham’s F-12 with GlutaMAX, 0.25% sodium bicarbonate, and 10 µg/ml hygromycin. Unless otherwise stated, cell culture products were from Thermo Fisher Scientific. Cells were routinely checked for mycoplasma contamination. When export of MMP14 was tested, HT 1080 cells were grown on dishes coated with collagen from calf skin (Sigma). For biotinylation experiments using 293T cells, they were grown on dishes coated with Poly-L-Lysine (Sigma). HeLa, 293T, and HT 1080 cells were transfected with Genejuice (Merck Millipore) or polyethylenimine (PEI) Max Linear 40 kD (Generon). For PEI transfection of 35-mm dishes, in general, 1 µg of each DNA was diluted in 100 µl OptiMEM, then complexed with 3 µg PEI prediluted in 100 µl OptiMEM. The mix was vortexed, incubated at RT for 15–20 min, and added dropwise to cells. For colocalization purposes, cells were generally examined 16–24 h after transfection. For all transfections of CFTR ΔF508, C4 (5 µM) and VX-809 (5 µM) were added 12 h after transfection.

For knockdown of protein expression in HeLa cells, cells were transfected with an shRNA-encoding plasmid, selected 24 h later with puromycin (10 µg/ml, 16 h), and then split. Experiments were performed after 88–96 h of knockdown. If shRNA cells were to be transfected, transfection was performed 72 h after the transfection of the shRNA. For knockdown of protein expression in 293T cells, cells were cotransfected with an shRNA-encoding plasmid and pCI N-RFP DEST, pCI GRAF2_res_-RFP, pCI RFP-WDR44_res_, or pCI RFP–WDR44_res_ ΔPro. Cells were split, transfected after 52–56 h, and collected 36 h later. For knockdown of protein expression in HT 1080 cells, cells were infected with lentivirus, selected 24 h later with puromycin (1 µg/ml, 16 h), and split. Cells were transfected 72 h after the original infection with pCI MMP14-GFP, alone or together with pCI RFP-WDR44_res_ or pCI RFP–WDR44_res_ ΔPro, and analyzed 16 h later. To make lentivirus, 293T cells were grown on Poly-L-Lysine–coated plates and cotransfected with shRNA, pMD2.G, and PSPAX2. Medium was changed to DMEM supplemented with 4% FBS and 25 mM Hepes after 24 h, and lentivirus was harvested as tissue culture supernatant after a further 48–60 h.

### Immunofluorescence

Cells grown on glass coverslips were fixed in 3.2% PFA diluted in culture medium (20 min, 37°C). For immunostaining of cytoplasmic proteins, coverslips were then washed in PBS and blocked in 5% normal goat serum (NGS) and 0.1% saponin (Sigma) in PBS. All further incubations were done in 1% NGS and 0.1% saponin in PBS. Coverslips were mounted on slides in a buffered PVA glycerol mountant containing 2.5% DABCO (Sigma). For staining of F-actin, Alexa Fluor 546–labeled phalloidin was added to the primary antibody dilution (final concentration, 0.4 U/ml). A similar protocol was followed for immunostaining of surface proteins under nonpermeabilizing conditions, but saponin was omitted from the buffers.

### Confocal microscopy

For live imaging, cells were grown on glass-bottom culture dishes (MatTek). Immediately before imaging, medium was replaced with phenol red–free DMEM containing 5% FBS and placed in a temperature-controlled chamber on the microscope stage with air/CO_2_ 95:5 and 100% humidity.

Most imaging data were acquired using a fully motorized inverted microscope (Eclipse TE-2000; Nikon) equipped with a CSU-X1 spinning disk confocal head (UltraVIEW VoX; PerkinElmer) using a 60× oil immersion lens (Plan Apochromat V, 1.4 NA; Nikon) under control of Volocity 6.0 (PerkinElmer). 14-bit digital images were acquired with a cooled EMCCD camera (9100–02; Hamamatsu Photonics). Three 50-mW solid-state lasers (488, 561, and 647 nm; CVI Melles Griot) coupled to individual acoustic-optical tunable filters (AOTF) were used as a light source to excite fluorescent proteins and dyes, as appropriate. Imaging of RUSH proteins was performed with a Zeiss LSM 780 using standard photomultiplier tubes, a 63× oil immersion lens of NA 1.4, and ZEN acquisition software.

ImageJ 1.48s (National Institutes of Health) was used to process the images, adjusting only the brightness and contrast of the different channels. Due to the high dynamic range of the signals and the fact that we were particularly interested in the dim structures in the cell’s periphery, we adjusted the scaling of the images to bring this information into the dynamic range of a computer display. No filters were applied. We followed the principles of Color Universal Design. On the merged channel, images acquired in the red channel are colored in red; images acquired in the green channel are colored in cyan; and images acquired in the far red channel are colored in magenta. Representative images of live cells and fixed cells collected in a minimum of three independent experiments are shown. Unless otherwise mentioned, images are from a single focal plane. For confocal stacks, maximum intensity projections of images acquired at 0.3-µm intervals, in single channels and as a merged image, are shown.

For quantification of the proportion of HeLa cells with RFP–Rab8a/10 tubules, cells were fixed 20 h after transfection. The number of cells with at least one tubule was counted and expressed as percentage of the total number of cells examined (minimum 25 cells per sample per experiment). For quantification of the total skeletal length of RFP–Rab8a/10 structures and for quantification of the total volume of GRAF1b/2–GFP structures per cell, cells were fixed 20 h after transfection. Confocal stacks were acquired. Each cell was identified as a region of interest. Volocity was used to identify fluorescent objects and measure their combined skeletal length or volume. Manders colocalization coefficients were quantified using Volocity, setting thresholds for each cell using a region of cytosol. For quantification of the proportion of HeLa cells with endogenous WDR44 tubules, cells were fixed 24 h (overexpression) or 96 h (shRNA) after transfection. The number of cells with at least one WDR44 tubule was counted and expressed as a percentage of the total number of cells examined (minimum 100 cells per sample per experiment). For quantification of the total WDR44 length per cell, confocal stacks were acquired. Each cell was identified as a region of interest. WDR44 objects with a nonspherical shape (shape factor <0.5) were identified using the Volocity software, and their skeletal length was measured. The total skeletal length per cell was quantified. For displaying fluorescence intensity profiles, fluorescence intensities were measured on a 1-px line using ImageJ software. Values were then normalized using the minimum and maximum values measured along that line. For detailed numbers of independent experiments, see the figure legends.

### Stimulated emission depletion (STED)

For super-resolution imaging, cells were plated on high-precision coverslips (custom order, 13-mm diameter, 0.17 ± 0.01–mm thickness, Hect-Assistant Glaswarenfabrik; Karl Hecht). Coverslips were processed as for all other immunofluorescence experiments, but antibodies were used at a higher concentration: mouse α-myc (1:100), rabbit α-Calnexin (1:200), Alexa Fluor 488 conjugated α-mouse (1:100), and Alexa Fluor 514 α-rabbit (1:100). Coverslips were mounted in Prolong Diamond Antifade Mountant (hard-setting, P36961; Thermo Fisher Scientific) curing for more than 48 h to achieve the best possible refractive index for STED microscopy.

The microscope was a Leica TCS SP8 STED 3X equipped with a pulsed white light laser and HyD hybrid detectors enabling gating. Gating was adjusted to detect signal emitting between 1.7 and 6.8 ns after the excitation pulse. Excitation of Alexa Fluor 488 was performed at 488 nm and of Alexa Fluor 514 at 535 nm. Suitable clean-up filters were in place. The depletion laser was at 592 nm.

The primary lens was a 100×/1.46 NA (orange, HC PL APO 100×/1.40 oil), chromatically corrected across the used spectrum. Sampling frequency was set to 20 nm, and the depletion laser was operated at settings that allowed a lateral resolution below 60 nm. As the photon budget was limited, line averaging (16×) and frame accumulation (8×) were used to achieve a suitable signal-to-noise ratio. ImageJ was used to process the images and to measure fluorescence intensity profiles across tubules.

### In vitro pull-downs

pGex 4T2, pGex 4T2 GRAF1 SH3, pGex 4T2 GRAF2 SH3, pGex 4T2 WDR44 ΔC, pGex 4T2 WDR44 ΔN2, pGex 4T2 MICAL1, pGex 4T2 MICAL1 ΔN, and pGex 4T2 MICAL1 ΔMOΔCH were transformed in BL21(DE3) pLysS *Escherichia coli* cells (Bioline), and protein expression was induced with isopropyl-β-D-thiogalactoside (150 µM, 16 h, 18°C). Cells were resuspended in bacterial lysis buffer (20 mM Hepes, pH 7.4, 1 mM DTT, cOmplete Protease Inhibitor Cocktail [Roche] with 500 mM NaCl for the empty vector and GRAF1/2 SH3; 200 mM NaCl for the others) and lysed by freezing/thawing and sonication. Lysates were cleared by centrifugation (30 min, 100,000 *g*) and incubated with glutathione sepharose beads for 45 min at 4°C under rotation. Beads were pelleted and washed three times in bacterial lysis buffer. Purified recombinant proteins were analyzed by electrophoresis on 4%–12% NuPAGE Novex Bis-Tris gels (Thermo Fisher Scientific) and Coomassie staining.

For pull-downs of cell lysates, beads were washed three times in cell lysis buffer (20 mM Hepes, pH 7.4, 150 mM NaCl, 2 mM EDTA, 1% IGEPAL CA-630 [Sigma], 1 mM DTT, and cOmplete Protease Inhibitor Cocktail). In parallel, 15-cm dishes of HeLa cells or rat brains were lysed in the same buffer (30 min, 4°C). Lysates were cleared by centrifugation (30 min, 100,000 *g*), and protein concentration of the supernatants was determined with a Bradford assay (Bio-Rad). Equivalent protein amounts of GST, GST-GRAF1 SH3, or GST-GRAF2 SH3 bound to glutathione sepharose beads were incubated with a volume of lysate corresponding to ∼7 mg protein or with the same volume of cell lysis buffer for 30 min at 4°C under rotation. Beads were pelleted (5 min, 500 *g*) and washed five times in cell lysis buffer without protease inhibitors. For identification of bound proteins by Western blot, beads were directly incubated with sample buffer, heated at 95°C, and loaded on gels. For identification of bound proteins by mass spectrometry, beads were incubated with Thrombin (90 min, 4°C). Eluted proteins were collected in the supernatant after centrifugation (5 min, 500 *g*), sample buffer was added, and proteins were separated using 4%–12% NuPAGE Novex Bis-Tris gels. Protein bands were revealed by Coomassie staining. Bands were excised, and proteins were identified by liquid chromatography-tandem mass spectrometry (LC-MS/MS; Thermo Orbitrap). Results were analyzed with Scaffold 3 (Proteome Software).

For in vitro pull-downs, GRAF2 SH3 was released from GST-GRAF2 SH3 beads by incubation with Thrombin (90 min, 4°C) and collected in the supernatant after centrifugation. WDR44 and MICAL1 domains bound to glutathione sepharose beads were then incubated in the presence or absence of an equal amount of GRAF2 SH3 for 30 min at 4°C and washed three times in bacterial lysis buffer. Proteins bound to the beads were separated using 4%–12% NuPAGE Novex Bis-Tris gels and revealed by Coomassie staining.

### Protein lysates, immunoprecipitation of endogenous proteins, and Western blotting

Untreated cells or shRNA-expressing cells (96 h after transfection) were scraped in cold PBS and pelleted. They were resuspended in lysis buffer (20 mM Hepes, pH 7.4, 150 mM NaCl, 2 mM EDTA, 1% IGEPAL CA-630, and cOmplete Protease Inhibitor Cocktail) and incubated 30 min at 4°C. Lysates were cleared (5 min, 14,000 *g*), and protein concentration was determined using a Bradford assay. Rat brains were homogenized in the same buffer using a Dounce homogenizer, but lysates were cleared at 100,000 *g*, 30 min. When lysates were to be directly analyzed, sample buffer was added to equal protein amounts (75 µg protein per sample); in general, for immunoprecipitation of endogenous proteins, 150 µg protein was incubated with purified antibodies or serum in a total volume of 100 µl and incubated 3 h at 4°C under rotation. 12.5 µl of a 50% suspension of Protein G Sepharose 4 Fast Flow beads (GE Healthcare Life Sciences) was added for another 30 min before beads were spun down (5 min, 500 *g*), washed three times in lysis buffer, and finally resuspended in sample buffer. Samples were heated 3 min at 95°C, loaded on 4%–12% NuPAGE Novex Bis-Tris gels, and analyzed by Western blot. Proteins were transferred to polyvinylidene fluoride membranes; powder milk was used as blocking agent. Secondary antibodies were detected using Amersham ECL detection reagent (GE Healthcare Life Sciences), and results were developed on x-ray film. The same protocol was followed for the coimmunoprecipitation of endogenous proteins from SH-SY5Y cells but using a volume of lysate corresponding to 100 cm^2^ of a confluent layer of cells for each immunoprecipitation.

For the identification of the GRAF proteins expressed in HeLa cells, 500 µg protein were used for each immunoprecipitation. For the identification of WDR44-binding proteins, lysates corresponding to a 40 cm^2^ confluent layer of 293T cells were pooled. In these two cases, a similar protocol was followed, but proteins were revealed by Coomassie staining of the gels. Bands were excised, and proteins were identified by LC-MS/MS.

For coimmunoprecipitation of endogenous proteins from HeLa cells, cells were resuspended in Triton X-100–containing lysis buffer (20 mM Hepes, pH 7.4, 150 mM NaCl, 2 mM EDTA, 1% Triton X-100, and cOmplete Protease Inhibitor Cocktail) and incubated 30 min at 4°C. Lysates were centrifuged (5 min, 14,000 *g*). The pellets were resuspended in the same volume of lysis buffer and cleared at low speed (5 min, 1,000 *g*), and a volume equivalent to 125 cm^2^ of a confluent layer of cells was used for each immunoprecipitation.

### Coimmunoprecipitation after overexpression in 293T cells

On the first day, 293T cells were plated in six-well dishes and a few hours later were transfected with the plasmid coding for the prey. The next day, cells were transfected with plasmids encoding the baits. 20–24 h later, the medium was removed, and cells were detached by pipetting in 1 ml cold PBS. Cells were spun down, and pellets were resuspended in 100 µl lysis buffer (20 mM Hepes, pH 7.4, 150 mM NaCl, 2 mM EDTA, 1% IGEPAL CA-630, and cOmplete Protease Inhibitor Cocktail) and incubated on ice for 30 min. Lysates were cleared (5 min, 14,000 *g*). 10 µl of each supernatant was collected, and 5 µl sample buffer was added (lysates). The rest of the supernatants were incubated with 12.5 µl of a 50% suspension of Protein G Sepharose 4 Fast Flow beads for 30 min at 4°C under rotation. Beads were then spun down (5 min, 500 *g*) and washed three times with 500 µl lysis buffer. Beads were resuspended in 10 µl sample buffer. Beads and lysates were heated 3 min at 95°C, loaded on 4%–12% NuPAGE Novex Bis-Tris gels, and analyzed in parallel by Western blot. Membranes from immunoprecipitations done with rabbit α-GFP antibody were first probed with mouse α-myc antibody. Corresponding beads were then probed with mouse α-GFP antibody while lysates were probed with rabbit α-GFP antibody. Membranes from immunoprecipitations done with mouse α-myc antibody were first probed with rabbit α-GFP antibody. Corresponding beads were then probed with rabbit α-myc antibody while lysates were probed with mouse α-myc antibody. For coimmunoprecipitation of uGRAF2 with rabbit α-GFP, uGRAF2 was detected in the beads with rabbit α-GRAF2 and HRP–Protein A. In general, the membranes corresponding to the beads were chosen to show the level of expression of the baits, but when the background was too strong, the lysates were shown. For immunoprecipitations using α-myc–coated beads, a similar procedure was followed, but 100-mm dishes were used. Cells were resuspended in 200 µl lysis buffer, 15 µl of each lysate was loaded per well, and 5 µl of α-myc–coated magnetic beads (Cat# 5698, RRID:AB_10707161; Cell Signaling Technology) were used per sample. In all cases, results displayed are representative of a minimum of three independent experiments.

For quantification of coimmunoprecipitations, band intensities in the beads and in the lysates were measured in triplicate using ImageJ software; background was removed. Within each experiment, the intensity of the prey found in the beads was normalized by the value obtained for the corresponding lysate and expressed as a ratio to the chosen reference. The reference sample and the numbers of independent experiments used for the quantifications are mentioned in each figure legend.

### HA-GLUT4-GFP export in differentiated 3T3 L1 cells

To induce differentiation, 3T3 L1 cells were grown to confluence in DMEM-GlutaMAX with 10% newborn calf serum. They were then fed with DMEM-GlutaMAX with 10% FBS, 0.5 mM 3-isobutyl-1-methylxanthine (IBMX), 0.25 µM dexamethasone, 1 µg/ml bovine insulin (all from Sigma), and 5 µM troglitazone (Cayman Chemicals). 2–3 d later, medium was replaced with the above without IBMX for another 2–3 d. Cells were then maintained in DMEM-GlutaMAX with 10% FBS for 2–3 d. HA-GLUT4-GFP was used to examine export of GLUT4 (Dawson et al., 2001). For transfection, cells were detached with accutase at 37°C, spun down at low speed, washed in PBS, and microporated (1500 V, 3 pulses, 10 ms) using the Neon Transfection system (Thermo Fisher Scientific). Cells were plated on 35-mm dishes coated with Poly-L-Lysine and Collagen from calf skin. 16 h later, cells were serum starved in DMEM-GlutaMAX containing 0.1% BSA for 2 h and, where indicated, stimulated with insulin (1 µg/ml, 30 min). Cells were delicately scraped in cold PBS and fixed in 3.2% PFA (15 min, on ice). Cells were spun down and blocked in 5% NGS in PBS at RT under gentle shaking. After 30 min, cells were pelleted (5 min, 500 *g*) and resuspended in 1% NGS in PBS containing DyLight 650 α-HA antibody (5 µg/ml) or unconjugated α-HA (1 µg/ml) and incubated for 90 min at RT. Cells were spun down, washed three times in 1% NGS in PBS, and resuspended in Alexa Fluor 647–conjugated α-mouse antibody in 1% NGS in PBS. Cells were incubated for another hour, washed three times, fixed once more in 3.2% PFA, and resuspended in PBS. Cells were analyzed by FACS using an LSRFortessa analyzer (BD Biosciences), and results were analyzed with FlowLogic 7.2.1 (Inivai Technologies Pty. Ltd.). After comparison with undifferentiated cells, differentiated cells were selected based on their light-scattering properties. GFP or GFP/RFP–positive cells were selected. Background was subtracted from the GFP and Alexa Fluor 647 fluorescence intensities. The ratio of the geometric mean of the Alexa Fluor 647 fluorescence intensity to the geometric mean of the GFP fluorescence intensity was calculated and expressed as a ratio to the value obtained for the corresponding insulin-treated control. This was used as a measurement of HA-GLUT4-GFP export efficiency. For all cotransfections of nonfluorescent proteins, coexpression of myc-tagged proteins or of MICAL1 was verified in parallel following a similar protocol but using buffers containing 0.1% saponin and α-myc (2 µg/ml) or α-MICAL1 (1.9 µg/ml). 50–1,000 differentiated cells were analyzed in each independent experiment.

### Integrin-β1 internalization

shRNA-expressing HeLa cells (96 h after transfection) were incubated for 1 h with Alexa Fluor 647–conjugated α-Integrin-β1 antibody (1 µg/ml) at 37°C. Cells were then washed in PBS, acid stripped (0.5% acetic acid, 0.5 M NaCl) for 45 s (Reineke et al., 2015), and either directly scraped and fixed in 3.2% PFA (uptake) or chased for 4 h at 37°C in prewarmed medium before being washed and stripped once more, scraped, and fixed (chase). Cells were pelleted (5 min, 500 *g*) and incubated in blocking solution (5% NGS in PBS) for 30 min at RT. All further incubations and washes were done in PBS with 1% NGS. Cells were pelleted and incubated with unconjugated α-Integrin-β1 antibody (5 µg/ml) for 90 min and washed three times. They were then incubated with Alexa Fluor 546–conjugated α-mouse antibody (2 µg/ml) for 1 h, washed three times, and fixed once more in 3.2% PFA before being resuspended in PBS. Cells were analyzed by FACS using an LSRFortessa analyzer, and results were analyzed with FlowLogic 7.2.1. Within each experiment, the geometric means of the Alexa Fluor 647 and the Alexa Fluor 546 intensities were normalized by the values obtained for the shControl uptake sample. For each shRNA, the ratio of the normalized intensity of internalized Integrin-β1 (Alexa Fluor 647) to the one of surface Integrin-β1 (Alexa Fluor 546) was calculated as a measurement of the internalized Integrin-β1 ratio. Any defect in degradation of the internalized Integrin-β1 antibody or in the recycling of the receptor should lead to a higher internalized Integrin-β1 ratio. Within each experiment, between 1,000 and 30,000 cells were analyzed in each sample.

### Protein export by FACS analysis

This assay was used to quantify the export of E-cadherin–GFP, E-cadherin–RFP, HA–α_2B_-AR–GFP, HA–α_2B_-AR–RFP, GFP-GPI, SBP-GFP–E-cadherin, SBP-GFP-GPI, E-cadherin–SBP-GFP, and VSVG-SBP-GFP in HeLa cells, of MMP14-GFP in HT 1080 cells, and of GFP–Extope CFTR and GFP–Extope CFTR ΔF508 in 293T cells. HeLa and HT 1080 cells were grown in 35-mm dishes. Unless otherwise specified in the figure legend, they were delicately scraped in 1 ml culture medium 16 h after transfection. 293T cells were grown in 12-well dishes and collected 36 h after transfection by pipetting. In all cases, cells were fixed in PFA (3.2%, 20 min, 37°C). Following fixation, all further incubations and centrifugations were done at RT. Cells were spun down (5 min, 500 *g*), resuspended in blocking solution (5% NGS in PBS), and incubated 30 min. Cells were then pelleted and resuspended in 50 µl of buffer (1% NGS in PBS) containing the appropriate primary antibody (α–E-cadherin: 8 µg/ml; α-MMP14: 4 µg/ml; Alexa Fluor 647 α-GFP: 2 µg/ml; rabbit α-GFP: 2.5 µg/ml; α-HA: 1 µg/ml; and α-VSVG: 5 µg/ml) and incubated for 90 min under gentle rocking. Cells were washed three times in 250 µl wash buffer (0.1% BSA in PBS). When unlabeled primary antibodies were used, cells were then incubated in a dilution of Alexa Fluor 647–labeled secondary antibody (2 µg/ml) in buffer (1% NGS in PBS) and incubated for 1 h under gentle rocking; cells were then washed three times in 250 µl wash buffer. After the final washes, cells were fixed once more in 3.2% PFA in PBS (20 min), washed, and resuspended in PBS. Cells were analyzed by FACS using an LSRFortessa analyser, and results were analyzed with FlowLogic 7.2.1. For single transfections, GFP-positive or RFP-positive cells were selected; for cotransfections, GFP- and RFP-positive cells were selected. Background was subtracted from the GFP, RFP, and Alexa Fluor 647 fluorescence intensities. The ratio of the geometric mean of the Alexa Fluor 647 fluorescence intensity to the geometric mean of the GFP or RFP (depending on the tag of the export candidate) fluorescence intensity was calculated and expressed as a ratio to the value obtained for the control. This was used as a measurement of protein export efficiency. Within each experiment, between 1,000 and 20,000 cells were analyzed in each sample.

### Biotinylation of surface proteins

Cells were delicately washed three times in cold PBS. Fresh EZ-Link Sulfo-NHS-LC-Biotin (Thermo Fisher Scientific; 0.170 mg/ml in biotinylation buffer [10 mM triethanolamine, pH 9, 140 mM NaCl]) was added to each well, and cells were gently rocked for 30 min at 4°C. Reaction was stopped by adding 1 ml of 100 mM glycine + 0.1% BSA in PBS. Cells were scraped, pelleted, washed delicately in Tris buffer (50 mM Tris, pH 7.4, 140 mM NaCl), and lysed (100 µl of 50 mM Tris, pH 7.4, 140 mM NaCl, 2 mM EDTA, 1% IGEPAL CA-630, 0.25% deoxycholate, and cOmplete Protease Iinhibitor Cocktail) for 30 min on ice. Lysates were cleared (5 min, 14,000 *g*), and 5 µl of each supernatant was removed (lysate). 10 µl of a 50% suspension of Neutravidin agarose beads (Thermo Fisher Scientific) was added to each sample, and they were then incubated 30 min at 4°C under rotation. Beads were pelleted (5 min, 500 *g*), washed three times in lysis buffer, and resuspended in sample buffer. Lysates and beads were heated 3 min at 95°C and analyzed by Western blot. For quantification of protein export efficiency, band intensities in the beads and in the lysates were measured in triplicate using ImageJ software; background was removed. Within each experiment, the intensity of the band in the beads was normalized by the corresponding value in the lysates and expressed as a ratio to the chosen reference.

### Statistical data analysis

Statistical data analysis was performed using Prism version 8.0.0 for Mac, GraphPad Software. For all quantifications provided, the means and SEM are shown. Unless otherwise stated, statistical data analysis was performed with one-way ANOVA of unmatched data. Gaussian distribution was assumed. Homoscedasticity was tested using a Brown-Forsythe test. The number of independent experiments (*n*) is detailed in the figure legends. In all cases, significant adjusted P values are represented as *, P < 0.05; **, P < 0.01; ***, P < 0.001; and ****, P < 0.0001.

In Fig. 1 D and Fig. 3 F, results were analyzed together; for each Rab, Dunnett’s multiple comparisons test with corresponding shControl-transfected cells was used. In Fig. 1 E and Fig. 3 E, results were analyzed together; for each Rab, Dunnett’s T3 multiple comparisons test with corresponding shControl-transfected cells was used. In Fig. 1, I and J, in each case and for each GRAF, Dunnett’s T3 multiple comparisons test with Rab8a-transfected cells was used. In Fig. 2, C and D; Fig. S2, D and E; Fig. 3 A; Fig. 4 A; and Fig. 6, D and F), in each case, Dunnett’s multiple comparisons test with the reference was used. In Fig. 2 I, Dunnett’s multiple comparisons test between pairs of GRAF1b/2–expressing cells transfected with mutant and corresponding wild-type protein was used. In Fig. 2 M, Fig. 4 D, Fig. 5 F, and Fig. 7 G, results were analyzed together with Dunnett’s multiple comparisons test using GFP-transfected cells as control; *n* = 24 for GFP-transfected cells. In Fig. 2 N, Fig. 4 E, and Fig. 6 M, results were analyzed together with Dunnett’s multiple comparisons test using shControl-transfected cells as control; *n* = 24 for shControl-transfected cells. In Fig. 4 I, Dunnett’s multiple comparisons test using DMSO-treated cells as control was used. In Fig. 4 J, Dunnett’s T3 multiple comparisons test using DMSO-treated cells as control was used. In Fig. 4 M, Dunnett’s T3 multiple comparisons test between corresponding Cytochalasin D and DMSO-treated samples was used. In Fig. 5, C, I and L, for each mutant, Tukey’s multicomparisons tests between all combinations of transfected proteins were used. In Fig. 5 D, for testing the effects of mutants on insulin-induced GLUT4 translocation, analysis of paired insulin-treated samples with Dunnett’s multiple comparisons tests using pCI or RFP-transfected cells as controls was used. For analyzing insulin-induced GLUT4 translocation within each transfection, Sidak’s multiple comparisons tests between paired untreated and insulin-treated samples were used. In Fig. 5 F, Sidak’s multiple comparisons tests between corresponding control and Cytochalasin D-treated cells were used. In Fig. 7 A, Dunnett’s multiple comparisons tests using methanol-treated cells as control were used. In Fig. 7 C and Fig. S7 C, for each protein, Dunnett’s T3 multiple comparisons test with corresponding methanol-treated cells was used. In Fig. 7 I, Sidak’s multiple comparisons tests between corresponding paired uptake and chase samples were used. In Fig. 8 A and Fig. 9 A, results were analyzed together. For each cargo, Dunnett’s multiple comparisons test using GFP/RFP-transfected cells as control was used. In Fig. 8 C, for each cargo, Dunnett’s multiple comparisons test using shControl-transfected cells as control was used. In Fig. 8 G, Tukey’s multiple comparisons test between all combinations of transfections was used. In Fig. 8 I, for each cargo, Dunnett’s multiple comparisons test using RFP-transfected cells as control was used. In Fig. 8 J, for each cargo, Dunnett’s multiple comparisons test using shControl + RFP-transfected cells as control was used. To assess rescue, paired *t* tests between shGRAF2a cells cotransfected with RFP and RFP-GRAF2_res_ and between shWDR44a cells cotransfected with RFP and RFP-WDR44_res_ or RFP–WDR44_res_ ΔPro were used. In Fig. 9 B, for each cargo, Dunnett’s multiple comparisons test with untreated cells as control was used. For GFP–Extope CFTR ΔF508, Sidak’s multiple comparisons test between corresponding RFP and RFP-Rab10 T23N-transfected cells was used. In Fig. 9 C, in each case, Dunnett’s multiple comparisons test of matched data with the corresponding control was used. In Fig. 9 E, Sidak’s multiple comparisons tests between samples incubated with or without biotin was used. In Fig. S9 E, unpaired *t* test between untreated and BFA-treated GFP-transfected cells and Dunnett’s multiple comparisons tests between BFA-treated transfected cells using BFA-treated GFP-transfected cells as control were used.

### Online supplemental material

Fig. S1 shows that similar to GRAF2, GRAF1b colocalizes with Rab8a/b and Rab10 and that GRAF2 is the predominant GRAF of HeLa cells. Fig. S2 contains the complete results for the protein pull-downs by GRAF1 SH3 and GRAF2 SH3 and additional biochemical data on the interaction of GRAF1b/2 with MICAL1 and WDR44. Fig. S3 contains additional imaging of GRAF1b/2 together with MICAL1 and WDR44 proteins. In Fig. S4, the interactions of MICAL1 and WDR44 with Rabs and the response of WDR44, GRAF1b/2, and MICAL1 to cytoskeleton-perturbing drugs are further detailed. Fig. S5 contains supplementary data on the dominant negative mutants MICAL1 G3W, WDR44 ΔC, and GRAF1/2 BAR-PH. Fig. S6 contains additional data on the WDR44-mediated contacts of GRAF1b/2 with the ER proteins VAPA/B. Fig. S7 contains supplementary results detailing the response of endogenous WDR44 to BFA. Fig. S8 contains additional data on the interplay between GRAF/WDR44, other members of the MICAL family, and recycling endosomes. Results presented in Fig. S9 further support a role for GRAF2 and WDR44 in the export of E-cadherin but not Occludin; Western blots show that knocking down WDR44 and GRAF2 does not cause ER stress; and additional imaging of SBP-GFP–E-cadherin is presented. In Video 1, time-lapse movies of HeLa cells transfected with GRAF1b, GRAF2, WDR44, and MICAL1 are run in parallel. The following videos correspond to time-lapse imaging of binary protein combinations: GRAF2 and MICAL1 in Video 2; GRAF2 and WDR44 in Video 3; MICAL1 and WDR44 in Video 4; Rab10 and MICAL1 in Video 5; Rab8a and MICAL1 in Video 6; Rab10 and WDR44 in Video 7; Rab8a and WDR44 in Video 8; and GRAF2 BAR-PH and WDR44 in Video 9. Video 10 shows the BFA-induced response of TGN46 and WDR44.
